# Presence of Per- and Polyfluoroalkyl Substances (PFAS) in Domestic Wastewater from Community Septic Systems in New Hampshire

**DOI:** 10.1021/acsestwater.6c00198

**Published:** 2026-08-04

**Authors:** Jennifer L. Harfmann, Stephen J. Roy, Andrew T. Koff, Brandon M. Kernen, Alexis Eaton

**Affiliations:** † 190508 New Hampshire Department of Environmental Services, Concord, New Hampshire 03302, United States; ‡ Civil and Environmental Engineering, University of New Hampshire, Durham, New Hampshire 03824, United States

**Keywords:** PFAS, septic, effluent, discharge, domestic, residential, community

## Abstract

Domestic wastewater has the potential to contain per- and polyfluoroalkyl substances (PFAS) and may impact groundwater when discharged to on-site septic systems. This study quantified and characterized PFAS in domestic wastewater effluent discharging to leach fields from four community septic systems in New Hampshire. Median monthly PFAS concentrations in wastewater effluent (sum of 40 PFAS) ranged from 7 to 88 ng/L, corresponding to a PFAS mass loading of 3–14 μg/person/day. Perfluorocarboxylic acids constituted 70% of PFAS molar mass overall, largely attributed to perfluoropentanoic acid, perfluorohexanoic acid, and perfluorooctanoic acid. More comprehensive PFAS analysis (extended targeted list of 84 PFAS, total oxidizable precursor assay, and adsorbable organic fluorine) indicated that precursors, and potentially fluorinated pharmaceuticals, were present in domestic wastewater effluent that were unaccounted for in standard PFAS methods, especially for conventional septic systems without an innovative/alternative onsite wastewater treatment system (I/A OWTS). Where I/A OWTSs are present, microbially mediated precursor transformations may alter PFAS signals in wastewater effluent discharged to leach fields. While challenges remain in characterizing a typical domestic wastewater PFAS signature, this study demonstrates that community septic systems that aggregate wastewater from individual households can aid in understanding community-level contributions of PFAS to the environment.

## Graphical abstract

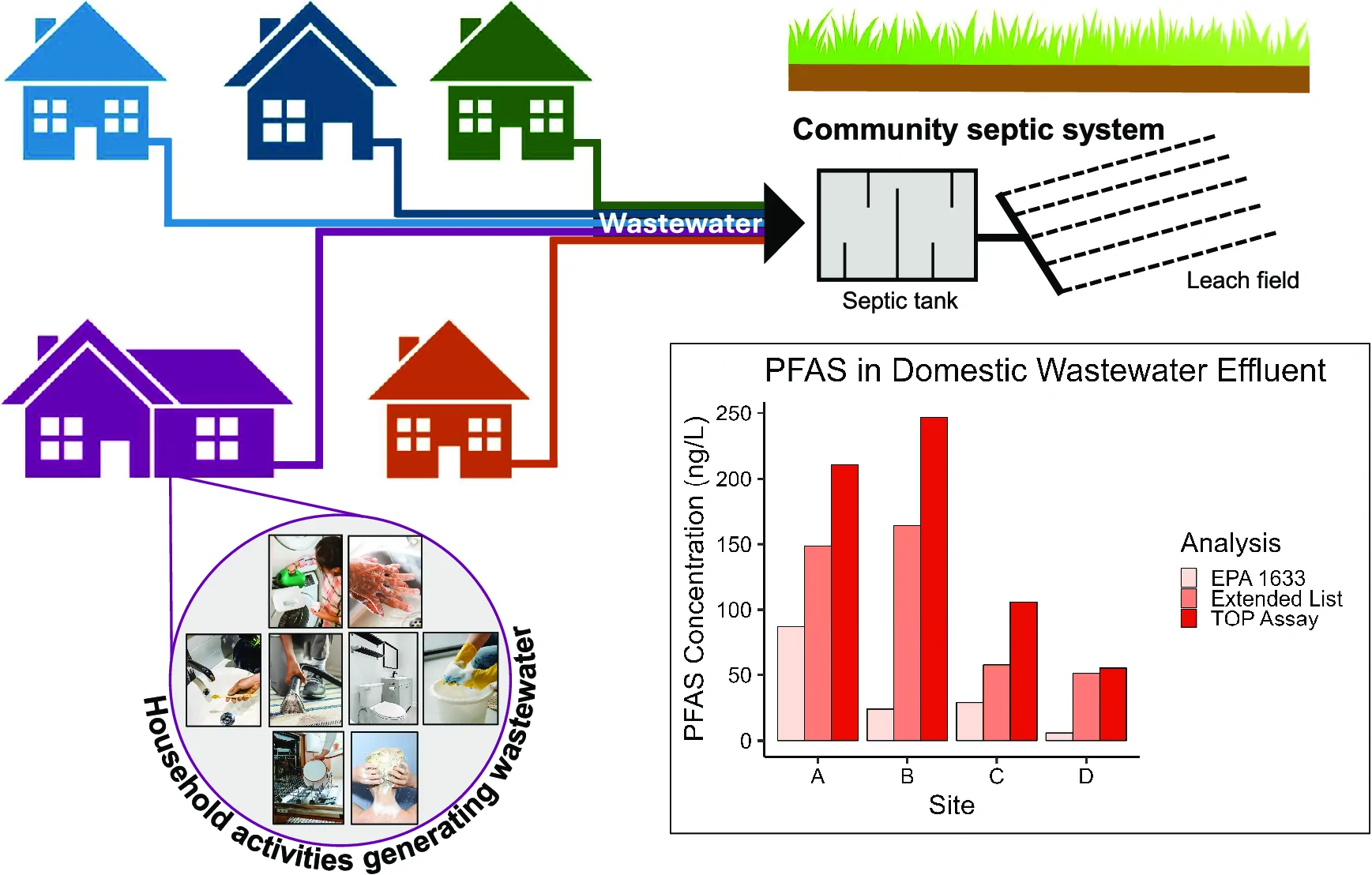

## 1 Introduction

Per- and polyfluoroalkyl substances (PFAS) are a class of synthetic fluorinated compounds present in a variety of household consumer products, including cosmetics, carpets, cleaning products, clothing, toilet paper, and children’s products.^1–8^ The prevalence of PFAS in the consumer product market, combined with an inherent resistance of these compounds to degrade in the environment, has led to widespread presence in not only industrial and commercial waste streams but also domestic waste streams. Household wastewater is generated from toilet-flushing and cleaning activities, such as hand-washing, showering, laundering, and dishwashing, and may contain PFAS depending on the products that are used. Sewershed studies at wastewater treatment facilities (WWTFs) indicate that residential customers can represent a large proportion (up to 98%) of the PFAS load discharged, particularly when accounting for precursor compounds.^9–12^ While most homes in the United States discharge wastewater to WWTFs, septic systems are relied upon in about 25% of homes, with that number doubling for homes located in rural areas.^13,14^ Septic system discharges to groundwater are of particular interest, given that they are often colocated on or near properties with private drinking water supply wells. Several studies have concluded that domestic septic systems impact the quality of groundwater sourced for drinking water using a multiple lines of evidence approach and/or septic system tracer studies in private wells.^15–17^ Characterizing the quantity and composition of PFAS in domestic wastewater effluent is therefore important in assessing impacts of septic systems relative to other point sources with regard to PFAS contamination in wells.

While there is a paucity of data on PFAS profiles of domestic wastewater discharged specifically from septic systems, a number of recent studies have quantified PFAS in domestic wastewater effluent from WWTF sewersheds. On average, using traditional targeted analytical methods, residential sewershed PFAS concentrations range between 10 and 50 ng/L with PFAS mass loadings (accounting for wastewater flow volumes) of up to 11 μg/person/day, primarily attributed to perfluorohexanoic acid (PFHxA) and perfluorooctanoic acid (PFOA).^9,11,12^ Targeted analytical methods can significantly underestimate PFAS when precursors present in the sample are not included in the method’s analyte list, and in such cases, contributions from precursor compounds can be approximated using analytical techniques such as the total oxidizable precursor (TOP) assay and bulk organofluorine. When using the TOP assay, average PFAS levels in residential sewershed discharges are elevated to between 80 ng/L and nearly 300 ng/L, demonstrating the important contribution of precursors to this waste stream.^11,18^ Identifying these precursor compounds is crucial in comprehensively characterizing PFAS in domestic wastewater. Integrated approaches leveraging the strengths of both targeted and nontargeted analytical methods can aid in PFAS profiling, as has been demonstrated in Penrose et al., whereby the TOP assay and an extended targeted analysis of 70 PFAS compounds were used to identify dialkyl perfluoroalkyl phosphates (diPAPs) as key precursors in domestic wastewater.^11^

Extending findings of residential sewershed studies at WWTFs to septic system discharges presents challenges when the goal is to characterize the PFAS concentrations and compositions. WWTFs are not passive conduits of PFAS and may promote precursor transformations through treatment processes within the plant that are not present in conventional septic systems.^19,20^ Given the substantial role that precursors play in contributing to the PFAS load in domestic wastewater, PFAS profiles in WWTF sewershed discharges may vary considerably from those of septic systems.

PFAS in residential septic system effluent has been assessed in a recent study of nine household septic tanks and confirms the prevalence of precursors, with high-resolution mass spectrometry screening resulting in tentative identifications of 40 additional PFAS not included in the study’s target list of 22 compounds.^21^ Targeted PFAS concentrations in septic tank effluent spanned 2 orders of magnitude, ranging from 42 to 9795 ng/L.^21^ High variability in PFAS concentrations underscores that, in contrast to WWTF sewershed studies that aggregate multiple input signatures, studies of individual household septic tanks provide household-specific data that can be quite diverse, and extrapolating to a wider scale is challenging.

Community septic systems are septic systems designed to aggregate wastewater discharges in a common geographic location and can be leveraged to understand domestic wastewater PFAS signatures discharging to septic system leach fields at a community level. Community septic systems may be conventional—consisting of a mixing chamber of multiple domestic wastewater streams discharging directly to leach fields—or they may include innovative/alternative onsite wastewater treatment systems (I/A OWTSs). I/A OWTSs optimize aerobic/anaerobic conditions to remove nitrogen from wastewater, in contrast to conventional septic systems that provide minimal passive biological treatment. Recent studies suggest that I/A OWTSs influence PFAS profiles in septic system wastewater effluent by transforming precursors to end products with greater fluorinated character, warranting further study on the impacts of I/A OWTS septic systems on groundwater relative to conventional septic systems.^21,22^

This study sought to quantify and characterize PFAS in domestic wastewater effluent discharging to leach fields from four community septic systems in New Hampshire. Secondary goals included (1) assessing the influence of I/A OWTSs on PFAS concentrations and composition in domestic wastewater effluent, (2) examining the utility of less traditional PFAS analytical techniques (e.g., TOP assay, bulk organofluorine, or expanded targeted PFAS lists) for these samples, and (3) estimating domestic-derived PFAS loadings to the environment from septic tank effluent and solids accrual.

## 2 Methods

### 2.1 Site Characterization

Sampling was conducted at four residential communities in New Hampshire that discharge domestic wastewater to community or shared septic systems (Table 1; Section S4). Sites were selected based on (1) absence of commercial or industrial inputs to the wastewater stream and (2) availability and ease of sampling access. Sites A and B are housing subdivisions, and Sites C and D are senior living facilities. Site A, located in Stratham, NH, consists of 24 two- to three-bedroom homes with year-round occupancy situated on approximately 5 acres of land. Wastewater from each home is directed to individual home septic tanks before flowing by gravity to a common holding tank and lift station on the property. From there, combined wastewater is pumped to an I/A OWTS (BioMicrobics SeptiTech) that uses a trickling filter technology under aerobic/anaerobic conditions for partial nitrification and then denitrification before discharging to one of three leach fields. Site B, located in Bartlett, NH, on approximately 50 acres of land, consists of 34 three-bedroom homes, approximately 20 of which have full-time occupancy. Wastewater from each home is directed to individual home septic tanks before being pumped to a holding tank on the property. Combined wastewater is pumped to one of four leach fields. Sites C and D are senior living facilities located on approximately 2 acres of land in Rye, NH, and approximately 30 acres of land in Benton, NH, respectively. Sites C and D house approximately 60–75 full-time residents and include common areas, a centralized kitchen and dining room, and laundry services. At Site C, wastewater flows from a single building to sequential septic tanks. Similar to Site A, wastewater is partially nitrified and denitrified using an I/A OWTS (AquaPoint Bioclere) before discharge to two onsite leach fields. At Site D, wastewater from 21 buildings is gravity-fed to a two-chambered septic tank designed for solids settling (Imhoff tank) and is directed to a holding tank. Wastewater then flows through four sand filter beds prior to discharging to three onsite leach fields. Drinking water is supplied to Sites B, C, and D by public water systems that require compliance with New Hampshire’s maximum contaminant levels (MCLs) for PFOA, perfluorooctanesulfonic acid (PFOS), perfluorononanoic acid (PFNA), and perfluorohexanesulfonic acid (PFHxS) of 12, 15, 11, and 18 ng/L, respectively. Site A is supplied by 24 individual bedrock wells. Six of these wells have been sampled for PFOA, PFOS, PFNA, and PFHxS, none of which had PFAS detections above the reporting limit.

**Table 1:** Characteristics of Community Septic System Facilities in This Study

site	A	B	C	D
facility type	housing subdivision	housing subdivision	senior living	senior living
seasonality	year-round	40% seasonal	year-round	year-round
population served (approx.)	70	60–100	60	70
wastewater flow (approx., GPD)	1500	3800	7500	7200
innovative/alternative onsite wastewater treatment system (I/A OWTS)?	yes	no	yes	no

### 2.2 Sampling Procedures

The primary focus of this work was to characterize domestic-derived PFAS released to the subsurface. As such, wastewater discharged to septic system leach fields (“effluent”) was sampled monthly at all four sites between October 2024 and September 2025. Samples were collected immediately prior to discharge to leach fields at Sites A, B, and C and prior to discharge to sand filter beds at Site D. Effluent grab samples were taken 6 in. from the bottom of each tank using a peristaltic pump connected to polyethylene tubing (3/8″ OD × 1/4″ ID) with a minimum purge time of 2 min.

Samples were collected in two 500 mL high-density polyethylene (HDPE) bottles. In months when additional PFAS analyses were planned (see below), larger volumes were collected per laboratory specifications (Table S1-B). Samples were stored at 4 °C and shipped overnight on ice to Onterris (formerly Enthalpy Analytical, LLC.) or SGS AXYS Analytical Services Ltd. (SGS AXYS) in accordance with laboratory requirements.

A secondary goal of this work was to understand potential influences of I/A OWTSs on PFAS in wastewater. At Sites A and C, additional samples were collected before treatment (“influent”) in March 2025 and June 2025. Influent grab samples were taken with single-use, cleaned (triple-rinsed with deionized water) HDPE buckets that were lowered into the holding tanks with nylon rope. Each bucket was rinsed three times with site influent water immediately before sample collection. As with effluent samples, influent samples were collected in 500 mL HDPE bottles and shipped overnight on ice to Onterris in accordance with laboratory requirements.

Drinking water was not sampled as part of this study. Because of the lack of contemporaneous data for drinking water and wastewater at these sites, PFAS contributions to wastewater from source water cannot be ruled out. However, the likelihood of such an influence is minimal, given that PFAS were nondetectable in well water in separate sampling efforts.

### 2.3 Supplemental Septic Tank Sampling

Additional sampling opportunities were afforded at Sites A and B to supplement the primary goals of this study. Septic tank pumping events at Sites A and B in September 2023 and July 2025, respectively, allowed for the collection of septic tank samples from three individual home septic tanks at each site. While wastewater influent and effluent sampling characterized domestic wastewater on an aggregate community-wide scale, individual septic tank sampling characterized the waste stream of discrete households within the community. A stainless steel sampling device was used to collect two samples from each tank: a liquid sample just below the surface before pumping (“supernatant”), and a solids sample after most of the tank had been pumped (“septage”). The sampling device was decontaminated between tanks using both deionized water and a 1% Liquinox solution. Samples were collected in two 500 mL HDPE bottles and shipped overnight on ice to Onterris in accordance with laboratory requirements.

### 2.4 Analytical Methods

All wastewater effluent and influent samples, as well as all septic tank supernatant and septage samples, were analyzed for 40 PFAS compounds using USEPA Method 1633A by Onterris or SGS AXYS.^23^ Per the method, samples were spiked with isotope-labeled surrogate standards, extracted via solid-phase extraction (SPE), and analyzed via liquid chromatography in tandem with mass spectrometry (LC-MS/MS). Method detection limits (MDLs) varied by analyte and matrix and can be found in Section S3 of the Supporting Information, with average MDLs of 1.9 ng/L in effluent, 1.2 ng/L in influent, 3.4 ng/L in septic tank supernatant, and 8.0 ng/g in septage samples.

In November 2024, February 2025, May 2025, and August 2025, additional PFAS analyses were conducted on wastewater effluent samples, including an extended targeted analysis of 84 PFAS compounds (14 perfluoroalkyl carboxylic acids (PFCAs), 11 perfluoroalkyl sulfonic acids (PFSAs), and 59 other precursor compounds), the total oxidizable precursor (TOP) assay (32 PFAS compounds), and adsorbable organic fluorine (AOF). The extended target analysis was composed of four analytical methods offered by SGS AXYS (MLA-110/USEPA 1633A, MLA-120/ASTM D8628-25, MLA-073, and MLA-121), each of which used isotope dilution followed by SPE and analysis by ultrahigh-performance liquid chromatography (UPLC-MS/MS). Samples analyzed for the TOP assay were oxidized via thermally activated persulfate at high pH before SPE and quantification by UPLC-MS/MS.^24^ AOF was analyzed following USEPA Method 1621, whereby samples were passed through a granular activated carbon column, rinsed with neutral nitrate, and quantified by combustion ion chromatography.^25^ Average MDLs in wastewater effluent samples analyzed for the extended targeted list, the TOP assay, and AOF were 3.8 ng/L, 12.7 ng/L, and 1.6 μg/L, respectively. See Section S1 of the Supporting Information for additional details, including the full list of PFAS analytes included in each method and MDLs.

Measures were taken to ensure quality control (QC) during the sample collection and laboratory analysis. A field blank (collected at Site A in December 2024) and two equipment blanks (collected during each home septic tank sampling event) were nondetect for 40 PFAS compounds (Table S3-A). Duplicate sampling and laboratory splits were incorporated into the sampling protocol at all four sites to assess data precision (Table S3-B).

Standard laboratory analytical QC procedures were followed, including calibration verification and routine quality-control batch analysis. Quality control batches included method blanks (MBs), laboratory control samples (LCSs), and laboratory control sample duplicates (LCSDs). Samples, LCSs, LCSDs, and MBs were re-extracted as needed (i.e., when recoveries of labeled isotopes in samples were outside of QC acceptance limits).

### 2.5 Data Analysis

PFAS detections above the MDL were included in data analysis for all samples and analytical methods in this study, given that 47% of detected data points fell below the reporting limit. All data qualifiers were evaluated on an individual basis before inclusion in the analysis.

The quantity of PFAS was compared across samples by summing PFAS concentrations in nanograms per liter (ng/L) for compounds included in each method (∑40 for USEPA Method 1633A and ∑84 for the extended list). The composition of PFAS was compared across samples by converting concentrations to mole fractions and assessing relative mole fractions of individual PFAS as well as mole fractions of PFCAs, PFSAs, and other precursor compounds.

PFAS mass loading from domestic wastewater effluent to leach fields was estimated at each site using monthly PFAS concentrations in effluent, wastewater flow data, and either the number of households (Sites A and B) or the number of residents (Sites C and D). PFAS mass in septic tank supernatant and solids was estimated using known septic tank dimensions, concentrations in sampled tanks, and the assumptions listed in Section S1 of the Supporting Information.

### 2.6 Statistical Analysis

Statistical analyses were conducted to evaluate differences in monthly PFAS concentrations (∑40) at each site. Statistical distributions were analyzed using a Shapiro–Wilk test. Since not all data were normal, medians were used to describe data, and Mann–Whitney *U* tests were employed to compare PFAS concentrations between sites. Statistical significance was defined as *p* < 0.05. The statistical analyses were performed using R Studio version 4.2.3.

## 3 Results and Discussion

### 3.1 Temporal Trends of PFAS in Domestic Wastewater Effluent

Median monthly PFAS concentrations (∑40) in wastewater effluent ranged from 7 to 88 ng/L across all four sites (Figure 1). These concentrations were comparable to those measured in other studies quantifying PFAS in domestic-derived wastewater from WWTF sewersheds, which ranged from 10 to 50 ng/L, and were generally lower than those found in domestic wastewater from individual home septic tanks, ranging from 42 to 9795 ng/L.^11,12,18,21^ Median PFAS concentrations were significantly higher at Site A (housing subdivision with I/A OWTS) relative to other sites (Mann–Whitney *U* test, *p* < 0.001), possibly due to a combination of household usage patterns and I/A OWTS precursor transformations as described below. In contrast to other sites, Site A exhibited a temporal trend of higher PFAS in effluent discharged in the fall relative to late winter and early spring. Previous studies at municipal WWTFs in New England found that seasonality impacted effluent concentrations through either temperature-induced microbial transformations within the WWTF or changes in influent PFAS composition.^19^ Since peak effluent PFAS concentrations at Site A did not correlate with peak ambient temperatures, variability in seasonal inputs of PFAS-containing household wastewater (due to, for example, changes in the type or quantity of PFAS-containing products used) was likely a driver in observed temporal patterns in addition to any temperature-induced changes to microbial activity in the I/A OWTS.

**Figure 1:**
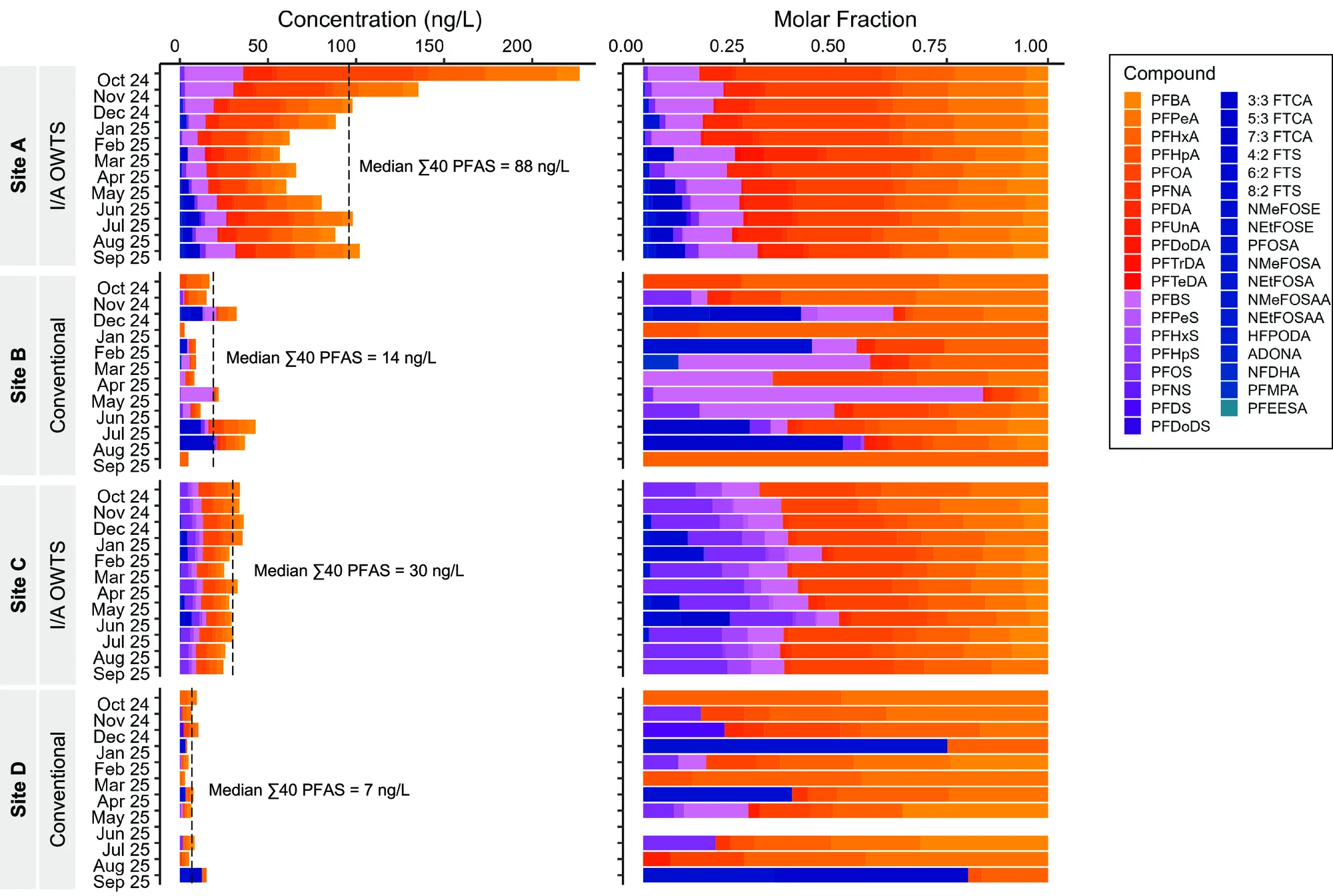
Monthly PFAS concentrations (ng/L) and molar fractions in wastewater effluent at Sites A through D from October 2024 through September 2025 as determined by USEPA Method 1633A. Dotted lines indicate the 12-month median total PFAS (∑40 PFAS) at each site. Shades of orange/red, purple, and blue indicate PFCAs, PFSAs, and precursor compounds, respectively.

On average, PFCAs constituted 70% of the PFAS molar mass in domestic wastewater effluent, primarily due to the prevalence of perfluoropentanoic acid (PFPeA), PFHxA, and PFOA, which together represented 55% of the median total PFAS molar mass. PFHxA and PFOA in particular are frequently reported as contributing disproportionately to PFAS detected in domestic wastewater effluent.^12,18^ Precursors (e.g., N-Methylperfluorooctanesulfonamidoethanol (NMeFOSE) and 5:3 fluorotelomer carboxylic acid (5:3 FTCA)) and PFSAs (e.g., perfluorobutane sulfonic acid (PFBS) and PFOS) were high during certain months, especially at locations with conventional septic systems (Sites B and D). FTCAs and PFOS have both been found consistently in domestic wastewater, but FOSEs and PFBS are less frequently detected.^12,18,21^ The PFAS composition was more variable month-to-month at sites with conventional septic systems (Table 2), demonstrating the potential that fluctuating consumer behavior (product type and usage) influences PFAS in wastewater discharges to conventional septic systems (and possibly systems with I/A OWTSs as well, although I/A OWTSs homogenize effluent signals; see discussion below). Variability at Site D, a senior living facility with onsite dining and laundry facilities, could reflect the timing of facility-wide laundering or cleaning operations, given that the samples were point-in-time grab samples. Site B, on the other hand, includes 30+ homes with partial seasonal occupancy, and variability observed in wastewater effluent at this site underscores the complexity of characterizing a single domestic-derived PFAS signature.

**Table 2:**
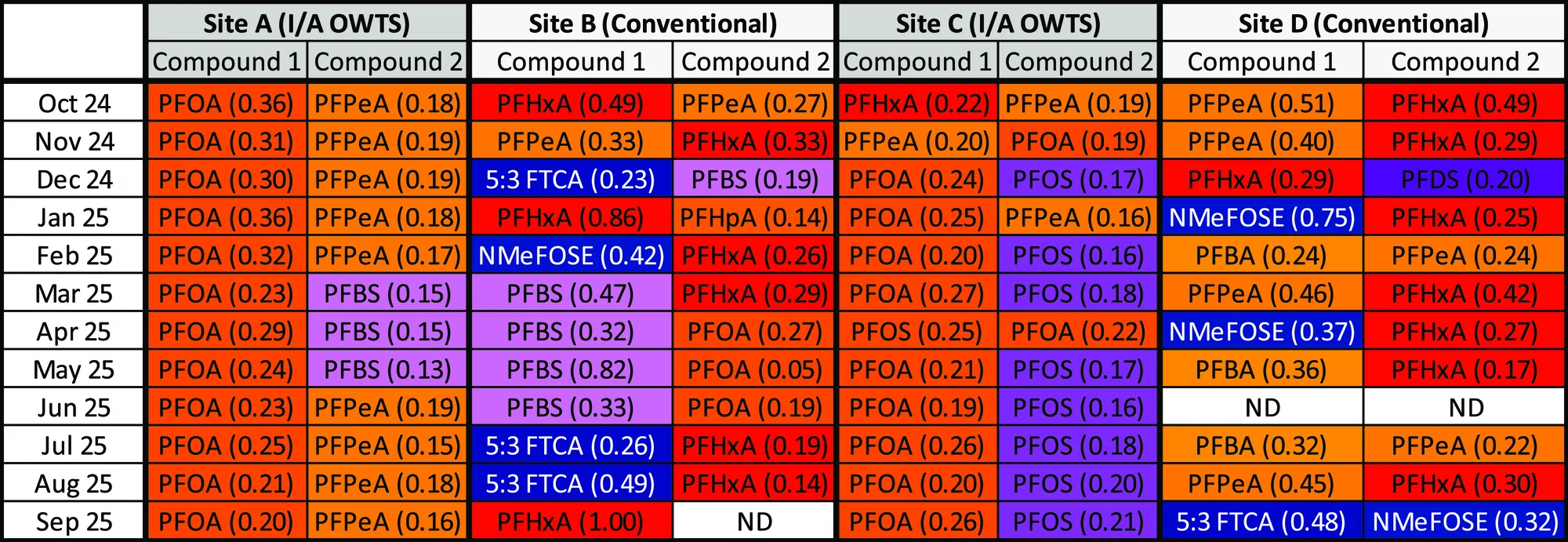
Monthly Highest Two PFAS Compounds By Molar Fraction For Each Site^a^

a ND indicates nondetect. Molar fractions for each PFAS compound are given in parentheses. Shades of orange/red, purple, and blue in each cell indicate PFCAs, PFSAs, and precursor compounds, respectively.

### 3.2 PFAS Mass Loadings to the Environment

Given that wastewater flow volumes varied considerably between sites, PFAS mass loadings were calculated from PFAS concentrations in order to evaluate per capita PFAS loads adjusted for wastewater flows at each site. Domestic PFAS mass loadings to leach fields ranged from 3 μg/person/day (Site D) to 14 μg/person/day (Site C) (Figure 2). Notably, PFAS mass loading at Site A was in the middle of this range (7 μg/person/day), despite having the highest PFAS concentrations, due to relatively low flows (see Table 1). PFAS mass loadings in this study were comparable to those estimated from a recent study of wastewater from residential neighborhood sewersheds in southern California (2.3–10.7 μg/person/day).^12^ Most other PFAS mass loadings reported in the literature were higher, and in some cases significantly higher (>100 μg/person/day).^12,18,26–34^ However, these values were primarily based on effluent from municipal WWTFs that comingle domestic, industrial, and commercial inputs and were therefore likely not representative of per capita mass loadings. PFAS mass loadings calculated both herein and in the literature were limited to the targeted PFAS compounds analyzed (40 compounds or fewer) and may have been underestimated when considering additional PFAS constituents as described below.

**Figure 2:**
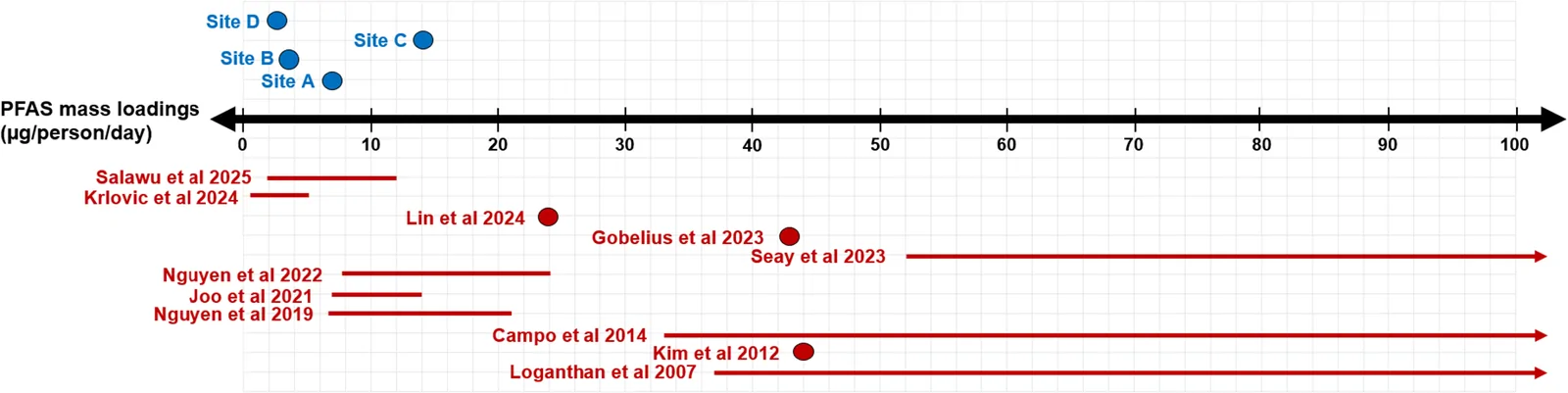
Per capita PFAS mass loadings (μg/person/day) approximated in this study from domestic wastewater effluent discharges to leach fields (top, in blue) compared to PFAS mass loadings reported in the literature from wastewater treatment plant and sewershed effluent (bottom, in red). Dots represent specific mass-loading values for studies that have reported values as such. For studies that report ranges of mass-loading values, lines represent ranges with arrows indicating that ranges exceeded 100 μg/person/day. Note that in most cases, literature values include a mix of industrial, commercial, and domestic sources.

### 3.3 Identifying Additional PFAS in Domestic Wastewater Effluent

Following its finalization in 2024, USEPA Method 1633A has quickly become the default method for PFAS analysis in wastewater samples, including those in this study. As with any targeted analytical method, USEPA Method 1633A is limited in scope to the PFAS compounds specified in the method. More comprehensive PFAS analysis of wastewater effluent—including an extended targeted list of 84 PFAS compounds, the TOP assay, and AOF—was conducted on a quarterly basis to quantify additional PFAS in domestic wastewater effluent not captured by USEPA Method 1633A. Compared to USEPA Method 1633A, the extended list detected 11 to >1000% more PFAS in wastewater effluent across all four sites (Figure 3), largely due to 6:2 fluorotelomer alcohol (6:2 FTOH) (63% detection frequency, average 19 ng/L), 6:2 diPAP (63% detection frequency, average 12 ng/L), 6:2 FTCA (75% detection frequency, average 7.4 ng/L), and 8:2 diPAP (56% detection frequency, average 6.2 ng/L). DiPAPs and FTCAs have previously been identified in residential septage and pump stations using a targeted list of 70 PFAS compounds.^11^ FTOHs are rarely included in targeted analyte lists, likely due to the volatility of these compounds necessitating specific bottle requirements and instrumental analysis. Their prevalence in domestic wastewater effluent at these sites suggests that inclusion may be warranted in future studies. The increase in PFAS concentrations using the extended list was greatest for sites with conventional septic systems (Sites B and D, with average increases of 621 and 797%, respectively) and lowest for those with I/A OWTSs (Sites A and C, with average increases of 76 and 103%, respectively). Observed differences were likely attributed to precursor transformations from the I/A OWTS, given that precursors present only in the extended list (e.g., FTOHs and diPAPs) are known to biotransform under certain environmental conditions to PFCAs detectable by USEPA Method 1633A (e.g., PFPeA and PFHxA).^21,35–37^

**Figure 3:**
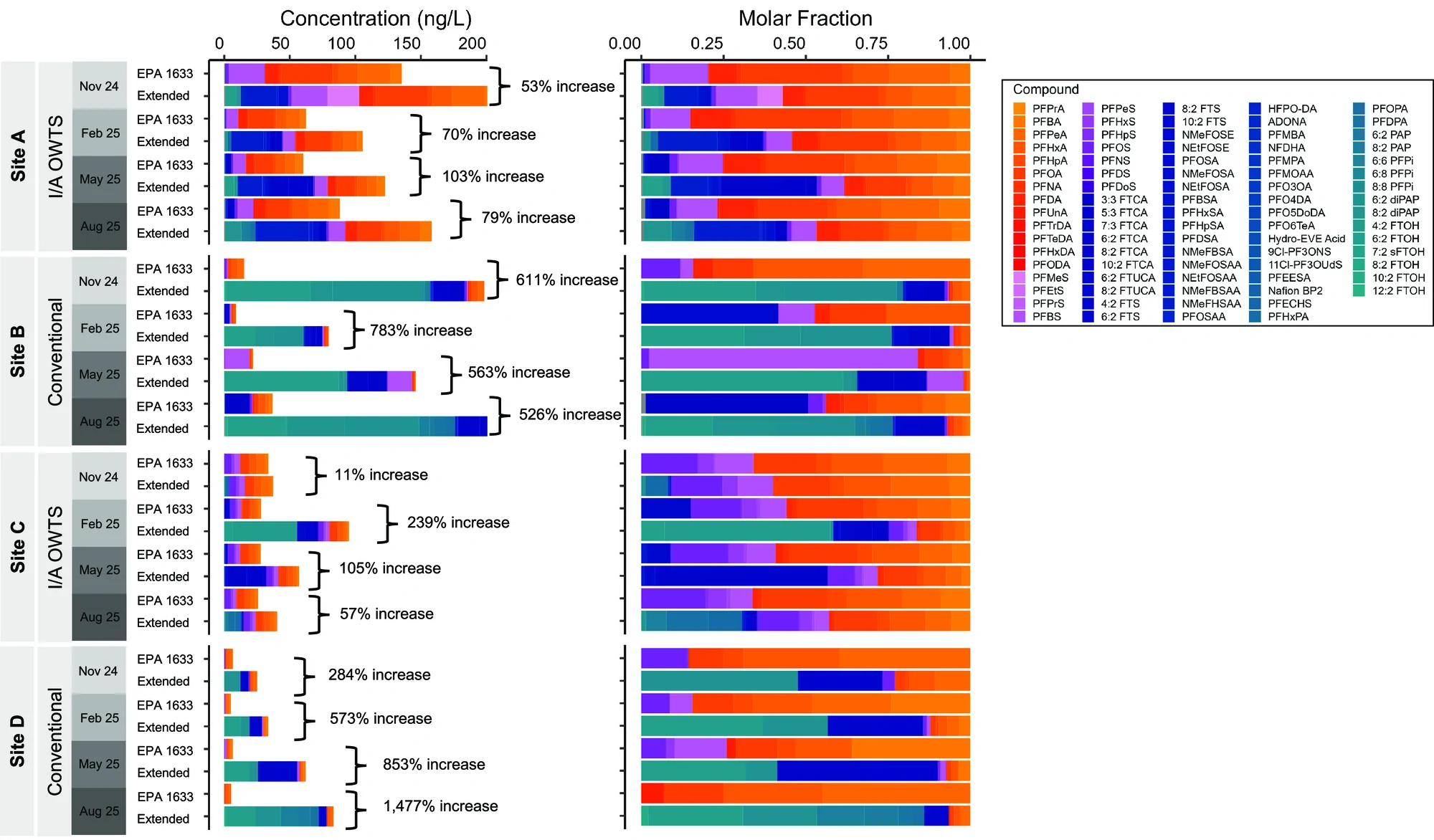
Quarterly PFAS concentrations (ng/L) and molar fractions in wastewater effluent at Sites A through D, comparing USEPA Method 1633A (EPA 1633) with an extended list of 84 PFAS compounds (extended). Percentage increases in total PFAS concentration from EPA 1633A to the extended list are annotated. Shades of orange/red, purple, and blue/green indicate PFCAs, PFSAs, and precursor compounds, respectively.

Both the TOP assay and AOF analysis provided further evidence that PFAS concentrations in domestic wastewater effluent were higher than the sum of 40 targeted PFAS compounds included in USEPA Method 1633A. Post-TOP concentrations in wastewater effluent were 2–30 times greater than pre-TOP concentrations across all sites (Figure 4), consistent with previous studies where post-TOP PFAS concentrations were 19–31 times greater than pre-TOP levels.^11,18^

**Figure 4:**
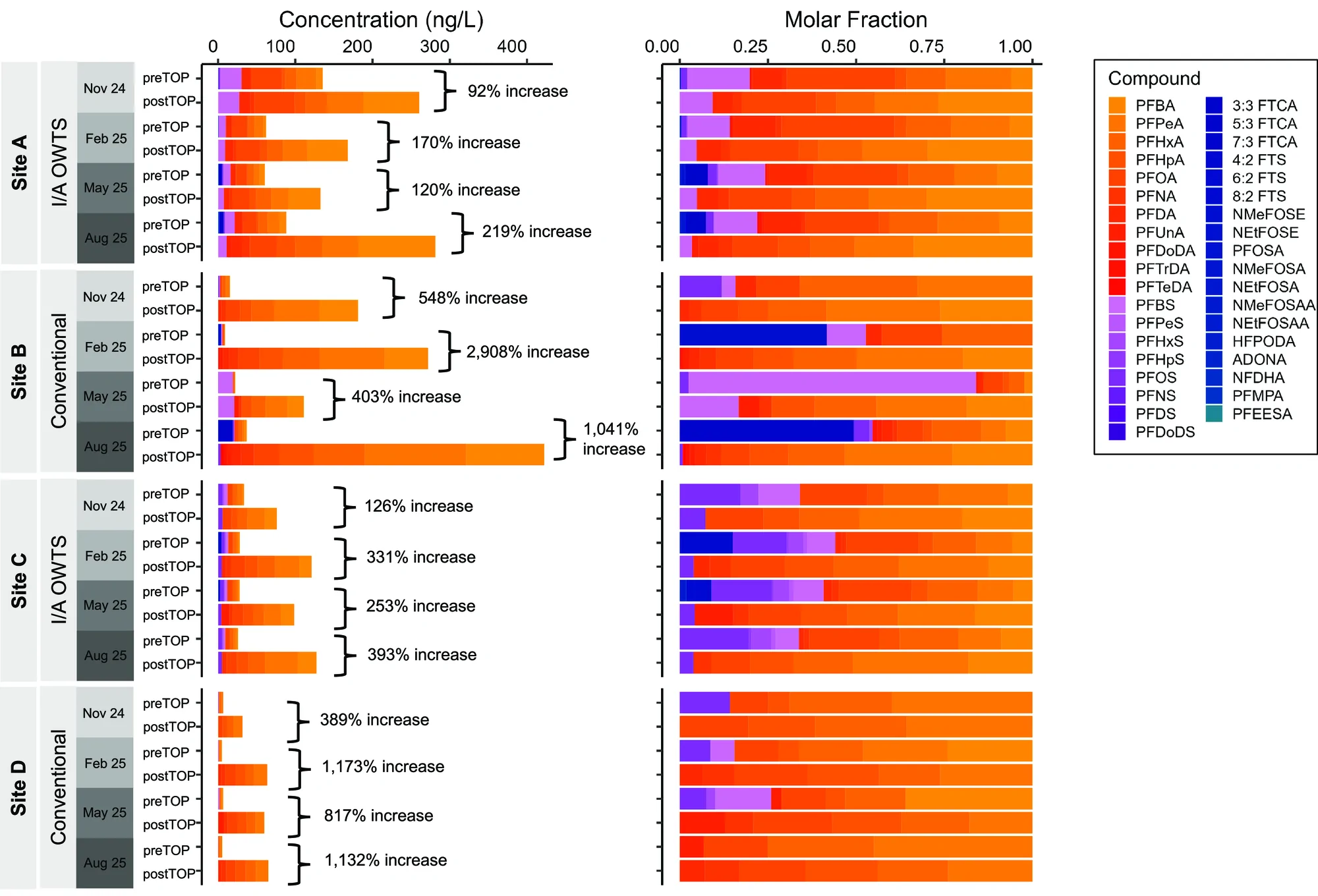
Quarterly PFAS concentrations (ng/L) and molar fractions in wastewater effluent at Sites A through D before (pre-TOP) and after (post-TOP) the total oxidizable precursor (TOP) assay. Percentage increases in total PFAS concentration from pre-TOP to post-TOP are annotated. Shades of orange/red, purple, and blue indicate PFCAs, PFSAs, and precursor compounds, respectively.

As with the extended targeted method, increases in concentration from pre-TOP to post-TOP were greatest for Sites B and D (average increases of 1225 and 878%, respectively) and lowest for Sites A and C (average increases of 150 and 276%, respectively). Sites B and D also had an average fluorine concentration around 4 μg/L (Figure 5)—several orders of magnitude higher than the average ∑40 PFAS concentration at these sites—in contrast to Sites A and C, where fluorine concentrations were at or below the MDL of 1.4–2 μg/L. Notably, Site D had both the lowest ∑40 PFAS and the highest AOF concentration detected of all four sites. Given that Site D is a senior living facility, the relatively high AOF concentration may reflect contribution from fluorinated pharmaceuticals, as has been observed in other studies.^38^

**Figure 5:**
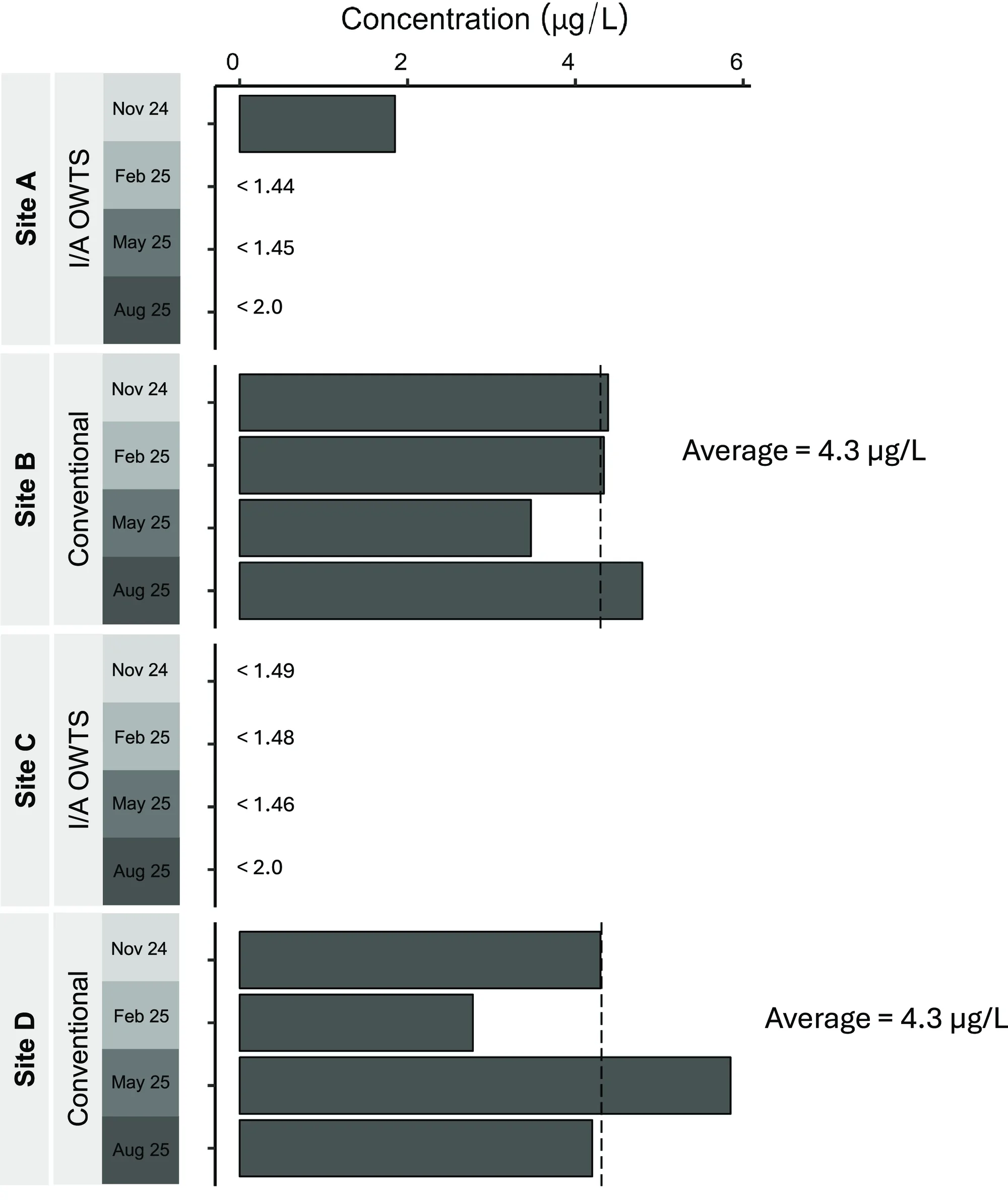
Quarterly adsorbable organic fluorine (AOF) concentrations (μg/L) in wastewater effluent as determined by USEPA Method 1621. Dotted lines indicate average AOF values at sites with three or more detections.

Overall, the results highlighted that precursors may be more prevalent in conventional septic system wastewater effluent than in effluent from WWTFs and I/A OWTS septic systems, given that WWTFs and I/A OWTS septic systems provide opportunities for many precursor compounds to be transformed before discharge. Consequently, USEPA Method 1633A may severely underestimate the total PFAS mass measured in wastewater effluent discharged from conventional septic systems.

### 3.4 Influence of I/A OWTSs on PFAS in Wastewater Effluent

The preceding discussion provides evidence that I/A OWTS septic systems likely allow for precursor transformations or other physicochemical mechanisms that affect PFAS signals detected in the wastewater effluent. To further investigate these impacts, PFAS detections in effluent were compared to corresponding influent samples at the two sites with I/A OWTSs in March and June 2025. At Site A, PFAS concentrations by USEPA Method 1633A were 3–19 times higher in effluent than influent (Figure 6). The comparatively large difference observed in spring (March 2025) relative to summer (June 2025) at Site A is counter to what would be expected if differences were attributed solely to temperature-driven microbial transformations and therefore supports earlier discussions that temporal variability in source inputs may have driven the extent of transformation in the I/A OTWS at Site A. Interestingly, in March, precursors (7:3 FTCA and perfluorooctanesulfonamide (PFOSA)) and PFSAs (PFBS) were detected in effluent that were not detected in influent. Concentrations of 7:3 FTCA and PFOSA in effluent were both near the detection limit (3.88 and 0.47 ng/L, respectively), such that the lack of detection in the influent did not preclude their presence. For PFBS, however, the concentration in the effluent (9.8 ng/L) was well above the detection limit (0.69 ng/L), which could indicate that PFBS may actively interact with the media in these treatment systems and may sorb/desorb depending on the biochemical environment in the system, an activity which, in turn, may vary based on ambient temperature.

**Figure 6:**
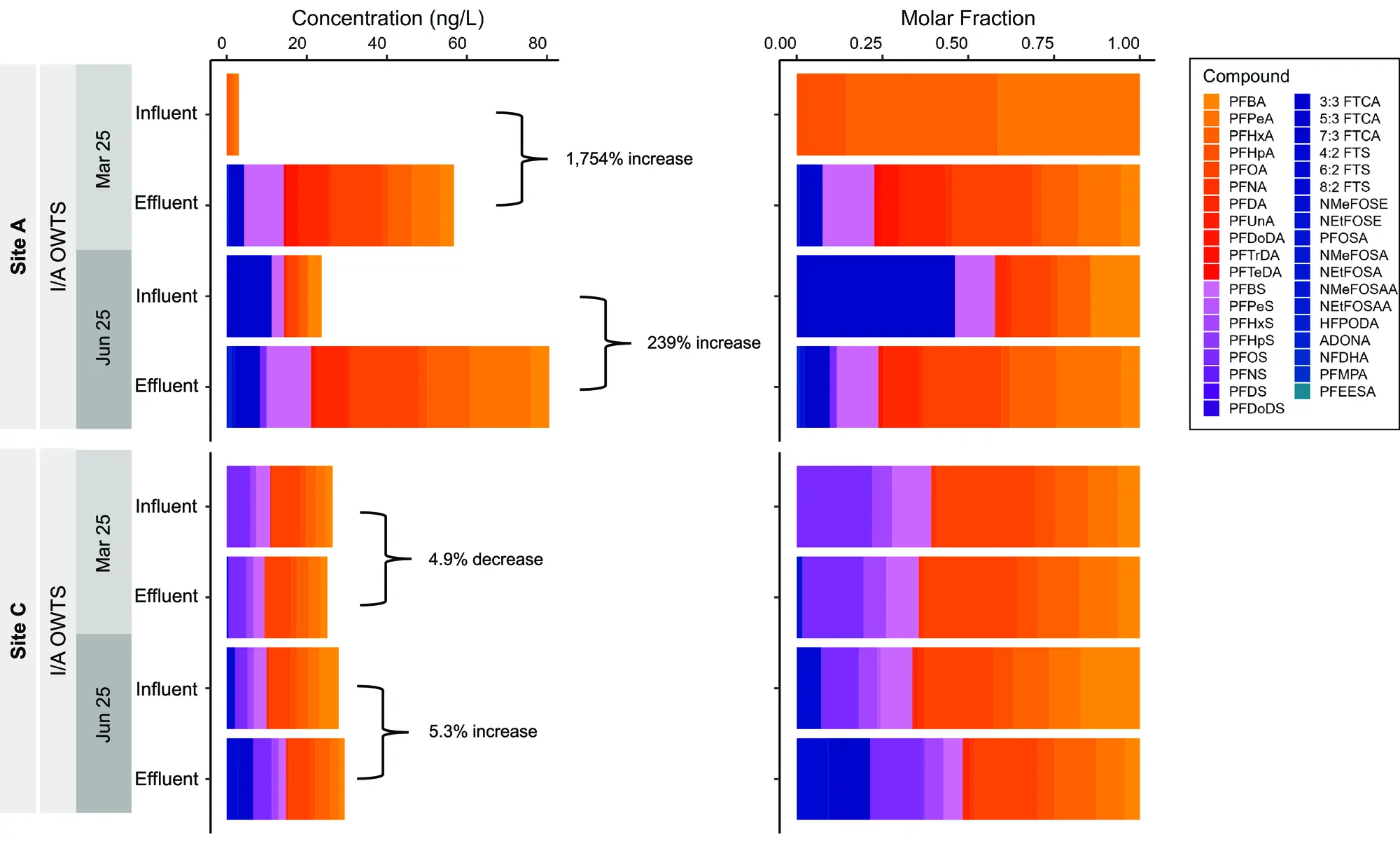
PFAS concentrations (ng/L) and molar fractions in wastewater influent and effluent at Sites A and C in March 2025 and June 2025 as determined by USEPA Method 1633A. The percentage change in total PFAS concentration from influent to effluent is annotated. Shades of orange/red, purple, and blue indicate PFCAs, PFSAs, and precursor compounds, respectively.

In contrast to Site A, PFAS at Site C did not change substantially between influent and effluent, with effluent concentrations within 5% of influent concentrations in both months (Figure 6). Both sites had similar treatment mechanisms (aerobic/anaerobic trickling filtration). However, sites differed in influent water chemistry (e.g., pH, dissolved oxygen, organic carbon, and PFAS concentrations) and system design/settings (e.g., type of media, recirculation rates, and pumping rates), both of which likely influenced microbial communities within each system capable of transforming precursors (measured or unmeasured). These trends are being explored in our additional studies.

### 3.5 PFAS in Domestic Septic Tank Supernatant and Septage

Considering that the domestic waste stream includes not only liquid wastewater discharged from the system to leach fields but also the solid fraction that settles out of solution, septic tank supernatant and septage (i.e., solids) were sampled for PFAS from each of three individual septic tanks at Sites A and B. With the exception of Site A Tank 1, PFAS concentrations (∑40) ranged from 12 to 33 ng/L in supernatant and <MDL to 4 ng/g in septage (Figure 7). Corresponding PFAS mass loadings, accounting for wastewater flow volumes and septage accrual rates, were estimated to be 45–120 μg/tank/year in supernatant and <MDL to 2200 μg/tank/year in septage. PFAS composition varied from one tank to the next, but longer-chain compounds, PFSAs, and certain precursors such as FTCAs were more prevalent in septage than supernatant. Observed partitioning patterns were consistent with other studies demonstrating that PFAS compounds with longer-chain lengths and/or sulfonate head groups often preferentially sorb to septage, sludge, and biosolids due to increased hydrophobicity.^19,39–41^ PFAS were detected in only two of the six septage samples; however, the elevated analytical detection limits for these samples (up to 72 ng/g; see Table S3-H) may have underestimated the presence of PFAS, and domestic septage may still be a significant reservoir for PFAS. In New Hampshire, 99% of septage is disposed of at WWTFs, increasing the PFAS burden at these facilities.^42^

**Figure 7:**
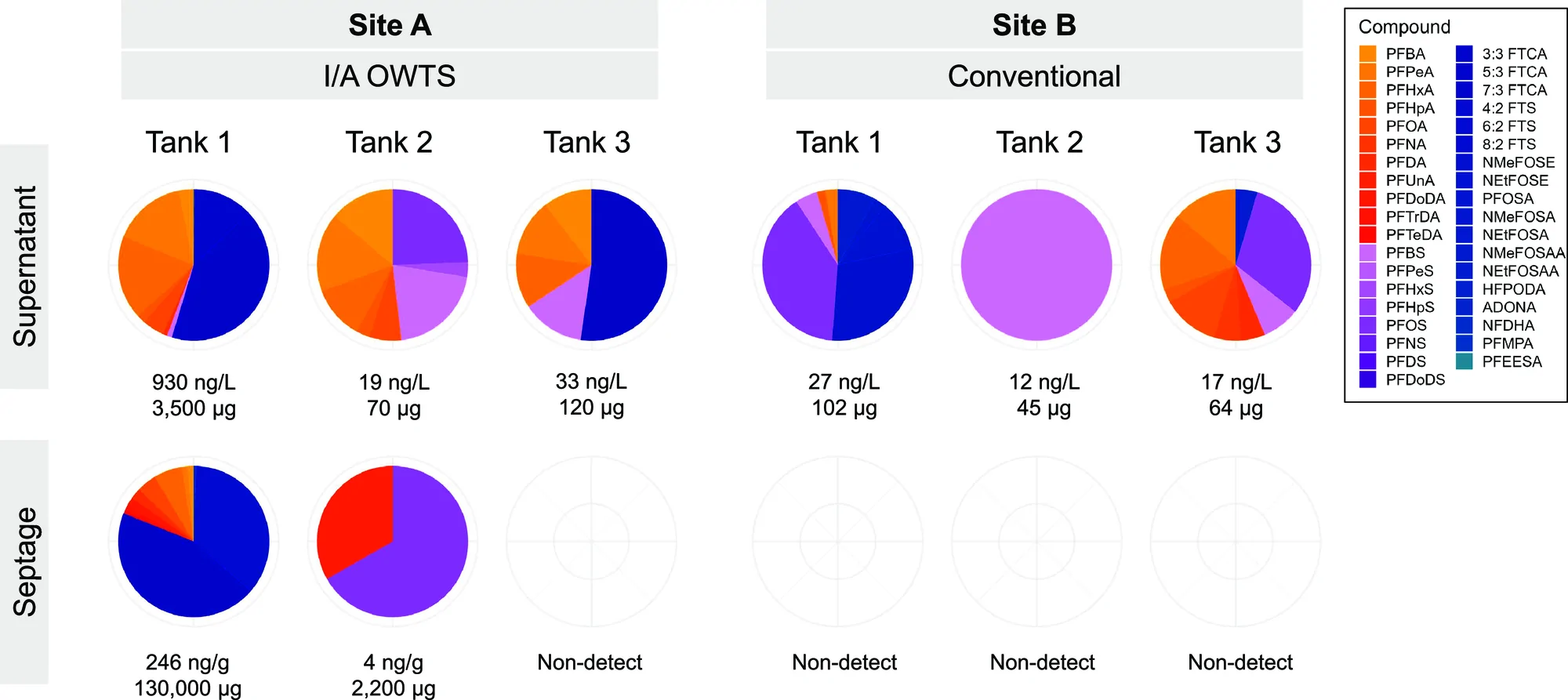
PFAS detected in three individual household septic tanks at Sites A and B as determined by USEPA Method 1633A. Pie plots represent PFAS molar fraction with corresponding concentrations (∑40 compounds in ng/L for supernatant or ng/g for septage) and estimated masses (∑40 compounds in μg) annotated. Detection limits ranged from 0.25 to 68 ng/L in supernatant and from 0.96 to 72 ng/g in septage. See Table S3-H for compound-specific detection limits. Shades of orange/red, purple, and blue indicate PFCAs, PFSAs, and precursor compounds, respectively.

Tank 1 at Site A was anomalous, containing much higher levels of PFAS relative to other tanks in both the supernatant (930 ng/L) and septage (246 ng/g), supporting findings of other studies demonstrating that PFAS concentrations can vary by orders of magnitude when sampling individual household septic tanks.^21^ While it is beyond the scope of this study to determine the specific factors leading to such high PFAS levels from this particular home, these results demonstrate that individual consumer behavior can have substantial impacts on PFAS in discrete domestic waste streams through a combination of product preferences (i.e., types of products used) and household practices that influence the quantity and quality of wastewater generated. While individual household septic tank sampling is valuable in providing granular information on PFAS household usage, aggregate community septic system sampling may be preferable if the goal is to characterize a more generalizable PFAS signature of domestic wastewater discharged to leach fields.

## 4 Conclusions

PFAS were detected in domestic wastewater effluent discharged to leach fields from four community septic systems in New Hampshire at levels between 7 and 88 ng/L (12-month median ∑40 PFAS), corresponding to a PFAS mass loading of 3–14 μg/person/day. PFCAs dominated the PFAS profile in domestic wastewater effluent at all sites, constituting 70% of the molar mass. At sites with conventional septic systems, precursors, such as 5:3 FTCA and NMeFOSE, were prevalent during certain months. Expanded PFAS sampling revealed significant presence of less frequently analyzed precursors such as 6:2 FTOH, 6:2 diPAP, 6:2 FTCA, and 8:2 diPAP. Results from both TOP and AOF analyses in wastewater effluent further supported that PFAS concentrations and estimated PFAS mass loadings—which were based on a sum of 40 targeted PFAS—were likely underestimated, particularly in conventional septic systems. At sites with I/A OWTSs, wastewater effluent had fewer measured and unmeasured precursor compounds (as determined by expanded targeted PFAS analysis and TOP assay, respectively) than those with conventional septic systems, indicating that I/A OWTSs may promote microbially mediated precursor transformations before discharge, depending on site-specific parameters. Septic tank solids may also be a reservoir for PFAS, though further study on the contributions of septic tank supernatant relative to solids is warranted. Altogether, this study supported increasing evidence that domestic septic systems contribute to PFAS in the subsurface through the discharge of wastewater effluent.

Characterizing a typical PFAS signature in domestic wastewater discharged to leach fields is a necessary step to distinguishing domestic sources of PFAS to groundwater from other industrial or commercial sources. Community septic systems offer a unique setting for exploring the bounds of an aggregate PFAS signature for domestic wastewater discharged to groundwater that is broad enough to characterize community-wide tendencies while also being granular enough to evaluate variability at the site level. For the four sites included in this study, domestic wastewater effluent was generally composed of greater than 50% PFCAs when analyzed by USEPA Method 1633A, greater than 90% PFCAs after the TOP assay, and greater than 50% precursors when FTOHs and diPAPs were included in the analytical suite. Identifying more specific trends in domestic wastewater discharged to leach fields is complicated by several factors highlighted in the study, namely, (1) compound-specific PFAS profiles vary considerably from one site to the next, particularly when comparing conventional septic systems with those that have I/A OWTSs, (2) PFAS profiles vary temporally within a single site, especially for sites with conventional septic systems, supporting that changes in consumer behavior will influence PFAS profiles even in community septic systems, and (3) routine PFAS analytical methods (e.g., USEPA Method 1633A) may lead to missed opportunities in PFAS profiling compared to expanded PFAS lists that are expensive and nonstandardized, but informative nonetheless. Still, work herein supported that PFAS are consistently detected in domestic wastewater effluent discharged to leach fields and warrants continued investigation of community septic system discharges to understand drivers of PFAS variability and impacts on groundwater quality.

## Supplementary Material



## Notes

PFAS analytical method details (Section S1), calculations and statistical tests (Section S2), data tables (Section S3), and site diagrams (Section S4) (PDF)
