# Supplementary material for: Presence of Per- and Polyfluoroalkyl Substances (PFAS) in Domestic Wastewater from Community Septic Systems in New Hampshire
Source: ACS ES T Water. 2026 Aug 4;6(8):4874–84. doi: 10.1021/acsestwater.6c00198 (PMC13488396; doi:10.1021/acsestwater.6c00198)

*Supporting Information for*

# Presence of Per- and Polyfluoroalkyl Substances (PFAS) in Domestic Wastewater from Community Septic Systems in New Hampshire

*Jennifer L. Harfmann,[*,†] Stephen J. Roy,[†] Andrew T. Koff,[†] Brandon M. Kernen,[†] and Alexis Eaton[‡]*

[†]New Hampshire Department of Environmental Services, Concord, New Hampshire 03302, United States

[‡]Civil and Environmental Engineering, University of New Hampshire, Durham, New Hampshire 03824, United States

[*]Email: Jennifer.L.Harfmann@des.nh.gov

This document contains the following sections:

**Section S1**: PFAS Analytical Method Details

**Section S2**: Calculations and Statistical Tests

**Section S3**: Data Tables

**Section S4**: Site Diagrams

# Section S1: PFAS Analytical Method Details

Section S1 provides details on analytical methods used in the study. Table S1-A lists all PFAS analytes with CAS numbers and indicates associated analytical methods. Table S1-B provides sampling information for each matrix and method. Method summaries are provided for each of SGS AXYS' Analytical Methods in the 'MLA' series.

# Table S1-A: PFAS Analytes

| Abbreviation | Chemical Name | CAS Number | Molecular Formula | Category | Regulated in NH | USEPA Method 1633 | Extended List SGS |
|---|---|---|---|---|---|---|---|
| PFPrA | Perfluoropropanoic acid | 422-64-0 | C3HF5O2 | PFCA | NO | NO | YES (MLA-120) |
| PFBA | Perfluorobutanoic acid | 375-22-4 | C4HF7O2 | PFCA | NO | YES | YES (MLA-110) |
| PFPeA | Perfluoropentanoic acid | 2706-90-3 | C5HF9O2 | PFCA | NO | YES | YES (MLA-110) |
| PFHxA | Perfluorohexanoic acid | 307-24-4 | C6HF11O2 | PFCA | NO | YES | YES (MLA-110) |
| PFHpA | Perfluoroheptanoic acid | 375-85-9 | C7HF13O2 | PFCA | NO | YES | YES (MLA-110) |
| PFOA | Perfluorooctanoic acid | 335-67-1 | C8HF15O2 | PFCA | YES | YES | YES (MLA-110) |
| PFNA | Perfluorononanoic acid | 375-95-1 | C9HF17O2 | PFCA | YES | YES | YES (MLA-110) |
| PFDA | Perfluorodecanoic acid | 335-76-2 | C10HF19O2 | PFCA | NO | YES | YES (MLA-110) |
| PFUnA | Perfluoroundecanoic acid | 2058-94-8 | C11HF21O2 | PFCA | NO | YES | YES (MLA-110) |
| PFDoDA | Perfluorododecanoic acid | 307-55-1 | C12HF23O2 | PFCA | NO | YES | YES (MLA-110) |
| PFTrDA | Perfluorotridecanoic acid | 72629-94-8 | C13HF25O2 | PFCA | NO | YES | YES (MLA-110) |
| PFTeDA | Perfluorotetradecanoic acid | 376-06-7 | C14HF27O2 | PFCA | NO | YES | YES (MLA-110) |
| PFHxDA | Perfluorohexadecanoic acid | 67905-19-5 | C16HF31O2 | PFCA | NO | NO | YES (MLA-121) |
| PFODA | Perfluorooctadecanoic acid | 16517-11-6 | C18HF35O2 | PFCA | NO | NO | YES (MLA-121) |
| PFMeS | Trifluoromethanesulfonate | 37181-39-8 | CF3O3S | PFSA | NO | NO | YES (MLA-120) |
| PFEtS | Perfluoroethanesulfonic acid | 354-88-1 | C2HF5O3S | PFSA | NO | NO | YES (MLA-120) |
| PFPrS | Perfluoropropanesulfonic acid | 423-41-6 | C3HF7O3S | PFSA | NO | NO | YES (MLA-120) |
| PFBS | Perfluorobutanesulfonic acid | 375-73-5 | C4HF9O3S | PFSA | NO | YES | YES (MLA-110) |
| PFPeS | Perfluoropentanesulfonic acid | 2706-91-4 | C5HF11O3S | PFSA | NO | YES | YES (MLA-110) |
| PFHxS | Perfluorohexanesulfonic acid | 355-46-4 | C6HF13O3S | PFSA | YES | YES | YES (MLA-110) |
| PFHpS | Perfluoroheptanesulfonic acid | 375-92-8 | C7HF15O3S | PFSA | NO | YES | YES (MLA-110) |
| PFOS | Perfluorooctanesulfonic acid | 1763-23-1 | C8HF17O3S | PFSA | YES | YES | YES (MLA-110) |
| PFNS | Perfluorononanesulfonic acid | 68259-12-1 | C9HF19O3S | PFSA | NO | YES | YES (MLA-110) |
| PFDS | Perfluorodecanesulfonic acid | 335-77-3 | C10HF21O3S | PFSA | NO | YES | YES (MLA-110) |
| PFDoS | Perfluorododecanesulfonic acid | 79780-39-5 | C12HF25O3S | PFSA | NO | YES | YES (MLA-110) |
| 3:3 FTCA | 3:3 Fluorotelomer carboxylic acid | 356-02-5 | C6H5F7O2 | Precursor | NO | YES | YES (MLA-110) |
| 5:3 FTCA | 2H,2H,3H,3H-Perfluorooctanoic acid | 914637-49-3 | C8H5F11O2 | Precursor | NO | YES | YES (MLA-110) |
| 7:3 FTCA | 3-(Perfluoroheptyl)propanoic acid | 812-70-4 | C10H5F15O2 | Precursor | NO | YES | YES (MLA-110) |
| 6:2 FTCA | 2-(Perfluorohexyl)ethanoic acid | 53826-12-3 | C8H3F13O2 | Precursor | NO | NO | YES (MLA-121) |
| 8:2 FTCA | 2-(Perfluorooctyl)ethanoic acid | 27854-31-5 | C10H3F17O2 | Precursor | NO | NO | YES (MLA-121) |
| 10:2 FTCA | 2-(Perfluorodecyl)ethanoic acid | 53826-13-4 | C12H3F21O2 | Precursor | NO | NO | YES (MLA-121) |
| 6:2 FTUCA | 2H-Perfluoro-2-octenoic acid | 70887-88-6 | C8H2F12O2 | Precursor | NO | NO | YES (MLA-121) |
| 8:2 FTUCA | 2H-Perfluoro-2-decenoic acid | 70887-84-2 | C10H2F16O2 | Precursor | NO | NO | YES (MLA-121) |
| 4:2 FTS | 4:2 Fluorotelomer sulfonic acid | 757124-72-4 | C6H5F9O3S | Precursor | NO | YES | YES (MLA-110) |
| 6:2 FTS | 6:2 Fluorotelomer sulfonic acid | 27619-97-2 | C8H5F13O3S | Precursor | NO | YES | YES (MLA-110) |
| 8:2 FTS | 8:2 Fluorotelomer sulfonic acid | 39108-34-4 | C10H5F17O3S | Precursor | NO | YES | YES (MLA-110) |
| 10:2 FTS | 10:2 Fluorotelomer sulfonic acid | 120226-60-0 | C12H5F21O3S | Precursor | NO | NO | YES (MLA-121) |
| NMeFOSE | N-Methyl-N-(2-hydroxyethyl)perfluorooctanesulfonamide | 24448-09-7 | C11H8F17NO3S | Precursor | NO | YES | YES (MLA-110) |
| NEtFOSE | N-Ethyl-N-(2-hydroxyethyl)perfluorooctane sulfonamide | 1691-99-2 | C12H10F17NO3S | Precursor | NO | YES | YES (MLA-110) |
| PFOSA | Perfluorooctanesulfonamide | 754-91-6 | C8H2F17NO2S | Precursor | NO | YES | YES (MLA-110) |
| NMeFOSA | N-Methylperfluorooctanesulfonamide | 31506-32-8 | C9H4F17NO2S | Precursor | NO | YES | YES (MLA-110) |
| NEtFOSA | N-Ethylperfluorooctanesulfonamide | 4151-50-2 | C10H6F17NO2S | Precursor | NO | YES | YES (MLA-110) |
| PFBSA | Perfluorobutanesulfonamide | 30334-69-1 | C4H2F9NO2S | Precursor | NO | NO | YES (MLA-121) |
| PFHxSA | Perfluorohexanesulfonamide | 41997-13-1 | C6H2F13NO2S | Precursor | NO | NO | YES (MLA-121) |
| PFHpSA | Perfluoroheptane sulfonamide | 82765-77-3 | C7H2F15NO2S | Precursor | NO | NO | YES (MLA-121) |

# Table S1-A: PFAS Analytes (cont'd)

| Abbreviation | Chemical Name | CAS Number | Molecular Formula | Category | Regulated in NH | USEPA Method 1633 | Extended List SGS |
|---|---|---|---|---|---|---|---|
| PFDSA | Perfluoro-1-decanesulfonamide | 4262-70-8 | C10H2F21NO2S | Precursor | NO | NO | YES (MLA-121) |
| NMeFBSA | N-(Methyl)nonafluorobutanesulfonamide | 68298-12-4 | C5H4F9NO2S | Precursor | NO | NO | YES (MLA-121) |
| NMeFOSAA | 2-(N-Methylperfluorooctanesulfonamido)acetic acid | 2355-31-9 | C11H6F17NO4S | Precursor | NO | YES | YES (MLA-110) |
| NEtFOSAA | 2-(N-Ethylperfluorooctanesulfonamido)acetic acid | 2991-50-6 | C12H8F17NO4S | Precursor | NO | YES | YES (MLA-110) |
| NMeFBSAA | N-Methyl perfluorobutane sulfonamidoacetic acid | 159381-10-9 | C7H6F9NO4S | Precursor | NO | NO | YES (MLA-121) |
| NMeFHSAA | 2-(N-Methyl perfluorohexanesulfonamido)acetic acid | 715646-50-7 | C9H6F13NO4S | Precursor | NO | NO | YES (MLA-121) |
| PFOSAA | Perfluorooctanesulfonamidoacetic acid | 2806-24-8 | C10H4F17NO4S | Precursor | NO | NO | YES (MLA-121) |
| HFPO-DA | Perfluoro-2-methyl-3-oxahexanoic acid | 13252-13-6 | C6HF11O3 | Precursor | NO | YES | YES (MLA-110) |
| ADONA | 4,8-Dioxa-3H-perfluorononanoic acid | 919005-14-4 | C7H2F12O4 | Precursor | NO | YES | YES (MLA-110) |
| PFMBA | Perfluoro(4-methoxybutanoic acid) | 863090-89-5 | C5HF9O3 | Precursor | NO | YES | YES (MLA-110) |
| NFDHA | Perfluoro-3,6-dioxaheptanoic acid | 151772-58-6 | C5HF9O4 | Precursor | NO | YES | YES (MLA-110) |
| PFMPA | Perfluoro-3-methoxypropanoic acid | 377-73-1 | C4HF7O3 | Precursor | NO | YES | YES (MLA-110) |
| PFMOAA | Perfluoro-2-methoxyaceticacid | 674-13-5 | C3HF5O3 | Precursor | NO | NO | YES (MLA-121) |
| PFO3OA | Perfluoro-3,5,7-trioxaoctanoic acid | 39492-89-2 | C5HF9O5 | Precursor | NO | NO | YES (MLA-121) |
| PFO4DA | Perfluoro-3,5,7,9-butaoxadecanoic acid | 39492-90-5 | C6HF11O6 | Precursor | NO | NO | YES (MLA-121) |
| PFO5DoDA | Perfluoro-3,5,7,9,11-pentaoxadodecanoic acid | 39492-91-6 | C7HF13O7 | Precursor | NO | NO | YES (MLA-121) |
| PFO6TeA | Perfluoro-3,5,7,9,11,13-hexaoxatetradecanoic acid | N/A | C8HF15O8 | Precursor | NO | NO | YES (MLA-121) |
| 9Cl-PF3ONS | Perfluoro(2-((6-chlorohexyl)oxy)ethanesulfonic acid) | 756426-58-1 | C8HClF16O4S | Precursor | NO | YES | YES (MLA-110) |
| 11Cl-PF3OUdS | 11-Chloroperfluoro-3-oxaundecanesulfonic acid | 763051-92-9 | C10ClF20O4S | Precursor | NO | YES | YES (MLA-110) |
| PFEESA | Perfluoro-2-ethoxyethanesulfonic acid | 113507-82-7 | C4HF9O4S | Precursor | NO | YES | YES (MLA-110) |
| Nafion BP 1 | Perfluoro-3,6-dioxa-4-methyl-7-octene-1-sulfonic acid | 29311-67-9 | C7HF13O5S | Precursor | NO | NO | YES (MLA-121) |
| Nafion BP2 | 5-(1,2,2,2-Tetrafluoro)ethoxy-perfluoro-3-oxa-4-methylpentanesulfonic acid | 749836-20-2 | C7H2F14O5S | Precursor | NO | NO | YES (MLA-121) |
| PFecHS | Perfluoro(perfluoroethyl)cyclohexanesulfonic acid | 133201-07-7 | C8HF15O3S | Precursor | NO | NO | YES (MLA-121) |
| PFHxPA | Perfluorohexyl phosphonic acid | 40143-76-8 | C6H2F13O3P | Precursor | NO | NO | YES (MLA-121) |
| PFOPA | Perfluorooctyl phosphonic acid | 40143-78-0 | C8H2F17O3P | Precursor | NO | NO | YES (MLA-121) |
| PFDPA | Perfluorodecyl phosphonic acid | 52299-26-0 | C10H2F21O3P | Precursor | NO | NO | YES (MLA-121) |
| 6:2 PAP | 6:2 Fluorotelomer phosphate monoester | 57678-01-0 | C8H6F13O4P | Precursor | NO | NO | YES (MLA-121) |
| 8:2 PAP | 8:2 Fluorotelomer phosphate monoester | 57678-03-2 | C10H6F17O4P | Precursor | NO | NO | YES (MLA-121) |
| 6:6 PFPi | Bis(tridecafluorohexyl)phosphinic acid | 40143-77-9 | C12HF26O2P | Precursor | NO | NO | YES (MLA-121) |
| 6:8 PFPi | Perfluorohexylperfluorooctyl phosphinic acid | 610800-34-5 | C14HF30O2P | Precursor | NO | NO | YES (MLA-121) |
| 8:8 PFPi | Bis(heptadecafluorooctyl)phosphinic acid | 40143-79-1 | C16HF34O2P | Precursor | NO | NO | YES (MLA-121) |
| 6:2 diPAP | 6:2 Fluorotelomer phosphate diester | 57677-95-9 | C16H9F26O4P | Precursor | NO | NO | YES (MLA-121) |
| 8:2 diPAP | 8:2 Fluorotelomer phosphate diester | 678-41-1 | C20H9F34O4P | Precursor | NO | NO | YES (MLA-121) |
| 4:2 FTOH | 4:2 Fluorotelomer alcohol | 2043-47-2 | C6H5F9O | Precursor | NO | NO | YES (MLA-073) |
| 6:2 FTOH | 6:2 Fluorotelomer alcohol | 647-42-7 | C8H5F13O | Precursor | NO | NO | YES (MLA-073) |
| 7:2 sFTOH | 7:2 sFluorotelomer alcohol | 24015-83-6 | C9H5F15O | Precursor | NO | NO | YES (MLA-073) |
| 8:2 FTOH | 8:2 Fluorotelomer alcohol | 678-39-7 | C10H5F17O | Precursor | NO | NO | YES (MLA-073) |
| 10:2 FTOH | 10:2 Fluorotelomer alcohol | 865-86-1 | C12H5F21O | Precursor | NO | NO | YES (MLA-073) |
| 12:2 FTOH | 12:2 Fluorotelomer alcohol | 39239-77-5 | C14H5F25O | Precursor | NO | NO | YES (MLA-073) |

**Table S1-B. Method-specific sample volume and bottle requirements**

| Sample Type | Method | Sample bottles/volume requirements |
|---|---|---|
| Effluent | USEPA Method 1633A (MLA-110) | 2-500 mL HDPE + 1 60 mL HDPE |
| | FTOHs (MLA-073) | 2-1L amber glass |
| | Ultra-Short via ASTM D8628-25 (MLA 120) | 1-60 mL HDPE |
| | Extended List (MLA-121) | 2-500 mL HDPE + 1-60 mL HDPE |
| | TOP Assay (MLA-111) | 2-60 mL HDPE |
| | AOF via USEPA 1621 (MLA-119) | 3-125 mL HDPE |
| Influent | USEPA Method 1633 (MLA-110) | 2-500 mL HDPE + 1 60 mL HDPE |
| Septic tank supernatant | USEPA Method 1633 (MLA-110) | 2500 mL HDPE |
| Septic tank septage | USEPA Method 1633 (MLA-110) | 2-500 mL HDPE |

**Method Summaries for MLA-Series Analytical Methods Provided by SGS AXYS**

**MLA-110**: Analysis of Per- and Polyfluoroalkyl Substances (PFAS) in Aqueous Samples, Solids, Tissues by LC-MS/MS. By EPA Method 1633A
Aqueous samples are extracted by solid phase extraction (SPE) using weak anion exchange cartridges; wash and elution procedures are chosen to meet various analysis requirements. Sample extracts are then treated with carbon powder and spiked with recovery standards. Analysis of the sample extract is performed on a UPLC-MS/MS (ultra-high-performance liquid chromatography) reversed phase C18 column using a solvent gradient. The column is coupled to a triple quadrupole mass spectrometer run at unit mass resolution in the Multiple Reaction Monitoring (MRM) mode, using negative electrospray ionization.

**MLA-073**: Analysis of Fluorotelomer alcohols (FTOH) in aqueous samples by UPLC-MS/MS
Aqueous samples are spiked with isotopically labeled surrogate standards, followed by liquid-liquid extraction with dichloromethane and dried with sodium sulfate. Extract cleanup is performed on SPE cartridges and/or powdered activated carbon. Analysis of the sample extract is performed on a UPLC-MS/MS (ultrahigh performance liquid chromatography) reversed phase C18 column using a solvent gradient. As in MLA-110, the column is coupled to a triple quadrupole mass spectrometer run at unit mass resolution in the Multiple Reaction Monitoring (MRM) in negative electrospray ionization mode.

**MLA-120**: Analytical Procedure for the Analysis of Ultrashort Chain Perfluoroalkyl Substances (Ultrashort chain PFAS) in Aqueous, Solid, Biosolid and Leachate Samples by UHPLC-MS/MS

After spiking with surrogate standards, aqueous samples are extracted by solid phase extraction (SPE) using weak anion exchange cartridges; wash and elution procedures are chosen to meet various analysis requirements. Analysis of the sample extract is performed on a UPLC-MS/MS (ultra-high-performance liquid chromatography) mixed mode column using a solvent gradient. The column is coupled to a triple quadrupole mass spectrometer run at unit mass resolution in the Multiple Reaction Monitoring (MRM) mode, using negative electrospray ionization.

**MLA-121**: Analytical Procedure for the Analysis of Extended list of PFAS in Aqueous, Solid, and Biosolid samples by UHPLC-MS/MS
Aqueous samples, typically 0.5 L, are spiked with isotopically labeled surrogate standards, extracted and cleaned up using weak anion exchanger SPE cartridge. Analysis of the sample extract is performed on a UPLC-MS/MS (ultrahigh performance liquid chromatography) reversed phase C18 column using a solvent gradient. The C18 column is coupled to a triple quadrupole mass spectrometer run at unit mass resolution in the Multiple Reaction Monitoring (MRM) mode, using negative electrospray ionization.

**MLA-111**: Analytical Procedure for the Analysis of Total Oxizidable Precursors (TOP) in Aqueous and Solid Matrices
Aqueous samples (up to 60 mL) are spiked with isotope labeled extraction surrogate standards before oxidation and oxidized using base and heat activated persulfate. After cooling and pH adjustment the reaction mixture is spiked with isotope labeled quantification standards and extracted and cleaned up using manual vacuum manifold or automated PromoChrom weak anion exchange SPE. Analysis of the sample extracts is performed on an ultrahigh performance liquid chromatography coupled to a triple quadrupole mass spectrometer, running manufacturer's MassLynx software. The mass spectrometer is run with unit mass resolution in the multiple reaction monitoring (MRM) mode.

**MLA-119**: Determination of Adsorbable Organic Fluorine (AOF) on Aqueous Samples by Combustion Ion Chromatography (CIC) by EPA Method 1621
The method targets organic compounds that are adsorbed onto at least 80 mg of granular activated carbon (GAC). After passing the sample through an activated carbon column, the carbon containing the adsorbed organic material is rinsed with neutral nitrate solution to remove inorganic salts. Analysis of sample extracts for AOF by CIC is performed on a Metrohm Profiler F Total Analyzer System, consisting of liquid chromatograph with ion suppression and coupled to an absorber unit and combustion furnace, running manufacturer's MagIC Net software. Two carbon cartridges are used in sequence, and each is analyzed separately to monitor breakthrough. Reported AOF results are the sum of the AOF on the two cartridges.

# Section S2: Calculations and Statistical Tests

Section S2 consists of details pertaining to PFAS mass loading calculations as well as Tables S2-A and S2-B with values from statistical tests used to assess differences in wastewater effluent concentrations across sites.

**PFAS Mass Loading Calculations**

PFAS mass loading from wastewater effluent was estimated at each site using monthly PFAS concentrations in effluent, wastewater flow data and either the number of households (Sites A and B) or the number of residents (Sites C and D) according to the following equation:

$$L_{Month\ X} = \left(\frac{(C_{PFAS\ 1} \times Q_{Month\ X} \times 3.78541\ L/gal)}{Z}\right) + \left(\frac{(C_{PFAS\ 2} \times Q_{Month\ X} \times 3.78541\ L/gal)}{Z}\right) + \cdots + \left(\frac{(C_{PFAS\ 40} \times Q_{Month\ X} \times 3.78541\ L/gal)}{Z}\right)$$

where, if Z = number of households, L (ng/house/day) is the per household PFAS mass loading in month X, C (ng/L) is the concentration of a given PFAS analyte, and Q (GPD) is the average daily wastewater flow in month X. If Z = number of residents, L (ng/person/day) is the per capita PFAS mass loading. Per capita PFAS mass loadings for Sites A and B were estimated assuming three residents per household. For Sites B, C, and D, water use data were used to estimate wastewater flow volumes.

PFAS mass in septic tank supernatant and solids was estimated using known septic tank dimensions, concentrations in sampled tanks, and the assumptions listed below. For supernatant,

$$M_{spn} = C_{spn,\sum 40} \times 3.78541 \frac{L}{gal} \times V_T$$

where $M_{spn}$ (ng) is the mass of PFAS in the supernatant, $C_{spn,\sum 40}$ (ng/L) is the total concentration of 40 PFAS analyzed in the supernatant, and $V_T$ is the volume of the tank. Supernatant was assumed to account for 100% of a 1,000 gallon tank.

For solids,

$$M_{sol} = C_{sol,\sum 40} \times A_T \times T_S \times 28{,}316.8 \frac{cm^3}{ft^3} \times \rho$$

where $M_{sol}$ (ng) is the mass of PFAS in the solids, $C_{spn,\sum 40}$ (ng/L) is the total concentration of 40 PFAS analyzed in the solids, $A_T$ ($ft^2$) is the surface area of the tank, $T_S$ (ft) is the thickness of the sludge layer, and ρ ($g/cm^3$) is the density of the sludge. Assumptions included a tank surface area of 43 $ft^2$, a sludge layer thickness of 1/3 ft, and a sludge density of 1.3 $g/cm^3$.

**Table S2-A. Shapiro-Wilk Test for Normality of Monthly Wastewater Effluent PFAS Concentrations**

| Site | N | W | p-value | Normally distributed? |
|---|---|---|---|---|
| A | 12 | 0.75 | 0.003 | No |
| B | 12 | 0.91 | 0.233 | Yes |
| C | 12 | 0.90 | 0.170 | Yes |
| D | 12 | 0.98 | 0.980 | Yes |

**Table S2-B. Mann-Whitney U Test of Monthly Wastewater Effluent PFAS Concentrations**

| Sites | U | p-value | Significant? |
|---|---|---|---|
| A / B | 0.00 | < 0.001 | Yes |
| A / C | 0.00 | < 0.001 | Yes |
| A / D | 0.00 | < 0.001 | Yes |
| B / C | 34.50 | 0.015 | Yes |
| B / D | 32.00 | 0.010 | Yes |
| C / D | 0.00 | < 0.001 | Yes |

## Section S3: Data Tables

Section S3 consists of Tables S3-A through S3-H, providing PFAS data (including method detection limits and data qualifiers), for all samples collected in this study. Tables S3-A through S3-C pertain to quality control/quality assurance sampling.

# Table S3-A: QC Data - Blanks

| Abbreviation | CAS Number | Field Blank | Equipment Blank | |
|---|---|---|---|---|
| | | Site A | Site A | Site B |
| PFPrA | 422-64-0 | | | |
| PFBA | 375-22-4 | < 1.60 | < 0.95 | < 1.68 |
| PFPeA | 2706-90-3 | < 0.42 | < 0.80 | < 0.44 |
| PFHxA | 307-24-4 | < 0.27 | < 0.44 | < 0.29 |
| PFHpA | 375-85-9 | < 0.27 | < 0.30 | < 0.28 |
| PFOA | 335-67-1 | < 1.78 | < 0.65 | < 1.86 |
| PFNA | 375-95-1 | < 0.24 | < 0.47 | < 0.25 |
| PFDA | 335-76-2 | < 0.43 | < 0.60 | < 0.45 |
| PFUnA | 2058-94-8 | < 0.41 | < 0.65 | < 0.43 |
| PFDoDA | 307-55-1 | < 0.22 | < 0.69 | < 0.23 |
| PFTrDA | 72629-94-8 | < 0.26 | < 0.56 | < 0.27 |
| PFTeDA | 376-06-7 | < 0.24 | < 0.40 | < 0.25 |
| PFHxDA | 67905-19-5 | | | |
| PFODA | 16517-11-6 | | | |
| PFMeS | 37181-39-8 | | | |
| PFEtS | 354-88-1 | | | |
| PFPrS | 423-41-6 | | | |
| PFBS | 375-73-5 | < 0.68 | < 0.50 | < 0.71 |
| PFPeS | 2706-91-4 | < 0.49 | < 0.26 | < 0.51 |
| PFHxS | 355-46-4 | < 0.51 | < 0.36 | < 0.53 |
| PFHpS | 375-92-8 | < 0.38 | < 0.40 | < 0.40 |
| PFOS | 1763-23-1 | < 1.17 | < 0.68 | < 1.23 |
| PFNS | 68259-12-1 | < 0.61 | < 0.46 | < 0.63 |
| PFDS | 335-77-3 | < 0.57 | < 0.34 | < 0.59 |
| PFDoS | 79780-39-5 | < 0.50 | < 0.57 | < 0.52 |
| 3:3 FTCA | 356-02-5 | < 1.58 | < 2.46 | < 1.66 |
| 5:3 FTCA | 914637-49-3 | < 6.78 | < 8.72 | < 7.09 |
| 7:3 FTCA | 812-70-4 | < 3.60 | < 5.88 | < 3.76 |
| 6:2 FTCA | 53826-12-3 | | | |
| 8:2 FTCA | 27854-31-5 | | | |

# Table S3-A: QC Data - Blanks (cont'd)

| Abbreviation | CAS Number | Field Blank | Equipment Blank | |
|---|---|---|---|---|
| | | Site A | Site A | Site B |
| 10:2 FTCA | 53826-13-4 | | | |
| 6:2 FTUCA | 70887-88-6 | | | |
| 8:2 FTUCA | 70887-84-2 | | | |
| 4:2 FTS | 757124-72-4 | < 1.27 | < 1.56 | < 1.33 |
| 6:2 FTS | 27619-97-2 | < 1.22 | < 2.01 | < 1.28 |
| 8:2 FTS | 39108-34-4 | < 1.87 | < 2.32 | < 1.96 |
| 10:2 FTS | 120226-60-0 | | | |
| NMeFOSE | 24448-09-7 | < 2.61 | < 3.50 | < 2.72 |
| NEtFOSE | 1691-99-2 | < 2.56 | < 1.82 | < 2.67 |
| PFOSA | 754-91-6 | < 0.40 | < 0.40 | < 0.42 |
| NMeFOSA | 31506-32-8 | < 1.01 | < 0.65 | < 1.06 |
| NEtFOSA | 4151-50-2 | < 0.97 | < 0.69 | < 1.02 |
| PFBSA | 30334-69-1 | | | |
| PFHxSA | 41997-13-1 | | | |
| PFHpSA | 82765-77-3 | | | |
| PFDSA | 4262-70-8 | | | |
| NMeFBSA | 68298-12-4 | | | |
| NMeFOSAA | 2355-31-9 | < 0.69 | < 0.81 | < 0.72 |
| NEtFOSAA | 2991-50-6 | < 0.69 | < 0.81 | < 0.72 |
| NMeFBSAA | 159381-10-9 | | | |
| NMeFHSAA | 715646-50-7 | | | |
| PFOSAA | 2806-24-8 | | | |
| HFPO-DA | 13252-13-6 | < 1.70 | < 0.83 | < 1.78 |
| ADONA | 919005-14-4 | < 1.59 | < 0.94 | < 1.67 |
| PFMBA | 863090-89-5 | < 0.45 | < 0.40 | < 0.47 |
| NFDHA | 151772-58-6 | < 1.55 | < 1.76 | < 1.62 |
| PFMPA | 377-73-1 | < 0.68 | < 0.43 | < 0.72 |
| PFMOAA | 674-13-5 | | | |
| PFO3OA | 39492-89-2 | | | |
| PFO4DA | 39492-90-5 | | | |

# Table S3-A: QC Data - Blanks (cont'd)

| Abbreviation | CAS Number | Field Blank | Equipment Blank | |
|---|---|---|---|---|
| | | Site A | Site A | Site B |
| PFO5DoDA | 39492-91-6 | | | |
| PFO6TeA | N/A | | | |
| 9Cl-PF3ONS | 756426-58-1 | < 1.92 | < 1.23 | < 2.01 |
| 11Cl-PF3OUdS | 763051-92-9 | < 1.93 | < 1.23 | < 2.02 |
| PFEESA | 113507-82-7 | < 0.37 | < 0.33 | < 0.39 |
| Nafion BP 1 | 29311-67-9 | | | |
| Nafion BP2 | 749836-20-2 | | | |
| PFecHS | 133201-07-7 | | | |
| PFHxPA | 40143-76-8 | | | |
| PFOPA | 40143-78-0 | | | |
| PFDPA | 52299-26-0 | | | |
| 6:2 PAP | 57678-01-0 | | | |
| 8:2 PAP | 57678-03-2 | | | |
| 6:6 PFPi | 40143-77-9 | | | |
| 6:8 PFPi | 610800-34-5 | | | |
| 8:8 PFPi | 40143-79-1 | | | |
| 6:2 diPAP | 57677-95-9 | | | |
| 8:2 diPAP | 678-41-1 | | | |
| 4:2 FTOH | 2043-47-2 | | | |
| 6:2 FTOH | 647-42-7 | | | |
| 7:2 sFTOH | 24015-83-6 | | | |
| 8:2 FTOH | 678-39-7 | | | |
| 10:2 FTOH | 865-86-1 | | | |
| 12:2 FTOH | 39239-77-5 | | | |

All values are in units of ng/L. CAS = Chemical Abstracts Service registry number.

## Table S3-B: QC Data - Duplicates

| Abbreviation | CAS Number | May-25 | | | | Aug-25 | | | |
|---|---|---|---|---|---|---|---|---|---|
| | | Site A | | Site D | | Site B | | Site C | |
| | | Dup 1 | Dup 2 | Dup 1 | Dup 2 | Dup 1 | Dup 2 | Dup 1 | Dup 2 |
| PFPrA | 422-64-0 | < 19.7 | < 20 | < 19.9 | < 19.6 | < 19.30 | < 19.10 | < 19.00 | < 19.40 |
| PFBA | 375-22-4 | 6.3 | 1.46 J | 2.39 J | 2.33 J | 2.84 J | 1.25 J | 2.39 J | 2.28 J |
| PFPeA | 2706-90-3 | 7.33 | 4.85 | < 1.17 | < 1.16 | 2.56 J | 2.71 J | 3.14 | 3.03 J |
| PFHxA | 307-24-4 | 7.63 | 6.27 | 1.06 J | 1.02 J | 4.91 | 5.45 | 4.21 | 4.69 |
| PFHpA | 375-85-9 | 1.8 | 1.7 | 0.333 J | 0.344 J | 0.876 J | 0.598 J | 1.44 J | 1.58 J |
| PFOA | 335-67-1 | 13.6 | 13.8 | 0.762 J | 0.734 J | 2.5 | 2.58 | 4.86 | 4.68 |
| PFNA | 375-95-1 | 1.06 J | 1.11 J | < 0.207 | < 0.206 | 0.429 K J | 0.427 J | 0.368 K J | 0.39 K J |
| PFDA | 335-76-2 | 6.05 | 5.8 | < 0.226 | 0.258 K J | 1.05 J | 0.956 J | 0.318 J | 0.3 J |
| PFUnA | 2058-94-8 | 0.216 K J | 0.165 J | 0.165 K J | 0.166 K J | 0.429 K J | 0.271 K J | < 0.14 | < 0.15 |
| PFDoDA | 307-55-1 | 0.319 J | 0.399 J | < 0.263 | < 0.263 | 0.456 K J | 0.498 K J | < 0.27 | < 0.28 |
| PFTrDA | 72629-94-8 | < 0.143 | < 0.14 | < 0.14 | < 0.14 | < 0.19 | < 0.18 | < 0.14 | < 0.15 |
| PFTeDA | 376-06-7 | < 0.21 | < 0.21 | < 0.21 | < 0.21 | < 0.275 | 0.314 K J | < 0.209 | < 0.22 |
| PFHxDA | 67905-19-5 | < 7.23 | < 7.33 | < 7.38 | < 7.22 | < 2.37 | < 2.38 | < 2.35 | < 2.36 |
| PFODA | 16517-11-6 | < 7.34 | < 7.45 | < 7.50 | < 7.34 | < 2.41 | < 2.42 | < 2.39 | < 2.39 |
| PFMeS | 37181-39-8 | < 9.83 | < 9.98 | < 9.96 | < 9.80 | < 9.66 | < 9.52 | < 9.49 | < 9.71 |
| PFEtS | 354-88-1 | < 4.94 | < 5.01 | < 5.01 | < 6.79 | < 4.97 | < 5.23 | < 4.89 | < 5.00 |
| PFPrS | 423-41-6 | < 4.93 | < 5.01 | < 5.00 | < 4.92 | < 4.92 | < 4.85 | < 4.83 | < 4.94 |
| PFBS | 375-73-5 | 9.2 | 6.71 | 1.15 K J | 1.19 K J | < 0.21 | < 0.20 | 1.96 | 2.14 |
| PFPeS | 2706-91-4 | < 0.143 | < 0.144 | < 0.141 | < 0.141 | < 0.19 | < 0.18 | 0.325 J | 0.332 J |
| PFHxS | 355-46-4 | 0.196 J | 0.231 J | 0.171 J | 0.135 J | 0.317 J | 0.181 J | 1.56 J | 1.34 J |
| PFHpS | 375-92-8 | < 0.13 | < 0.14 | < 0.13 | < 0.13 | < 0.18 | < 0.17 | 0.174 J | 0.176 J |
| PFOS | 1763-23-1 | 1.57 | 1.55 | 0.48 J | 0.585 J | 1.63 J | 1.59 J | 5.11 | 4.9 |
| PFNS | 68259-12-1 | < 0.12 | < 0.13 | < 0.12 | < 0.12 | < 0.16 | < 0.15 | < 0.12 | < 0.13 |
| PFDS | 335-77-3 | < 0.11 | < 0.11 | < 0.10 | < 0.10 | < 0.14 | < 0.13 | < 0.11 | < 0.11 |
| PFDoS | 79780-39-5 | < 0.16 | < 0.16 | < 0.16 | < 0.16 | < 0.21 | < 0.20 | < 0.16 | < 0.17 |
| 3:3 FTCA | 356-02-5 | < 0.69 | < 0.69 | < 0.68 | < 0.68 | < 0.90 | < 0.85 | < 0.68 | < 0.72 |
| 5:3 FTCA | 914637-49-3 | 4.16 J | 3.85 J | < 3.67 | < 3.67 | 19 J | 21.2 J | < 3.71 | 3.91 J |
| 7:3 FTCA | 812-70-4 | < 5.60 | < 5.64 | < 5.52 | < 5.51 | < 7.34 | < 6.91 | < 5.58 | < 5.86 |
| 6:2 FTCA | 53826-12-3 | < 5.40 | < 5.44 | < 5.32 | < 5.31 | 16.6 | 16.5 | < 4.74 | < 4.75 |
| 8:2 FTCA | 27854-31-5 | < 0.94 | < 0.95 | < 0.93 | < 0.93 | < 4.77 | < 4.81 | < 4.74 | < 4.75 |
| 10:2 FTCA | 53826-13-4 | < 14.60 | < 14.80 | < 14.90 | < 14.60 | < 4.77 | < 4.81 | < 4.74 | < 4.75 |
| 6:2 FTUCA | 70887-88-6 | < 2.89 | < 2.93 | < 2.95 | < 2.89 | < 0.95 | < 0.95 | < 0.94 | < 0.94 |
| 8:2 FTUCA | 70887-84-2 | < 1.45 | < 1.47 | < 1.48 | < 1.44 | < 0.47 | < 0.48 | < 0.47 | < 0.47 |
| 4:2 FTS | 757124-72-4 | < 0.97 | < 0.98 | < 0.96 | < 0.96 | < 1.27 | < 1.20 | < 0.97 | < 1.02 |
| 6:2 FTS | 27619-97-2 | < 5.40 | < 5.44 | < 5.32 | < 5.31 | < 7.07 | < 6.67 | < 5.38 | < 5.65 |
| 8:2 FTS | 39108-34-4 | < 0.94 | < 0.95 | < 0.93 | < 0.93 | < 1.24 | < 1.17 | < 0.94 | < 0.99 |
| 10:2 FTS | 120226-60-0 | < 2.89 | < 2.93 | < 2.95 | < 2.89 | < 0.947 | 1.47 | 1.43 | 1.71 |
| NMeFOSE | 24448-09-7 | < 2.15 | < 2.16 | < 2.11 | < 2.11 | < 2.81 | < 2.65 | < 2.14 | < 2.25 |
| NEtFOSE | 1691-99-2 | < 2.58 | < 2.60 | < 2.54 | < 2.53 | < 3.37 | < 3.18 | < 2.57 | < 2.69 |
| PFOSA | 754-91-6 | 0.648 J | 1.02 J | < 0.41 | < 0.41 | < 0.55 | < 0.52 | < 0.42 | < 0.44 |
| NMeFOSA | 31506-32-8 | < 0.19 | < 0.19 | < 0.19 | < 0.19 | < 0.25 | < 0.24 | < 0.19 | < 0.20 |
| NEtFOSA | 4151-50-2 | < 0.55 | < 0.56 | < 0.55 | < 0.54 | < 0.73 | < 0.68 | < 0.55 | < 0.58 |
| PFBSA | 30334-69-1 | 3.03 | 3.1 | < 0.30 | < 0.29 | 0.284 K | 0.34 K | 0.233 | 0.191 |
| PFHxSA | 41997-13-1 | < 0.29 | < 0.29 | < 0.30 | < 0.29 | < 0.09 | < 0.10 | < 0.09 | < 0.09 |

# Table S3-B: QC Data - Duplicates (cont'd)

| Abbreviation | CAS Number | May-25 | | | | Aug-25 | | | |
|---|---|---|---|---|---|---|---|---|---|
| | | Site A | | Site D | | Site B | | Site C | |
| | | Dup 1 | Dup 2 | Dup 1 | Dup 2 | Dup 1 | Dup 2 | Dup 1 | Dup 2 |
| PFHpSA | 82765-77-3 | < 0.29 | < 0.29 | < 0.30 | < 0.29 | < 0.09 | < 0.10 | < 0.09 | < 0.09 |
| PFDSA | 4262-70-8 | < 0.29 | < 0.29 | < 0.30 | < 0.29 | < 0.09 | < 0.10 | < 0.09 | < 0.09 |
| NMeFBSA | 68298-12-4 | 1.53 K | 1.17 K | < 0.59 | < 0.58 | < 0.20 | < 0.19 | < 0.19 | < 0.19 |
| NMeFOSAA | 2355-31-9 | < 0.33 | < 0.34 | < 0.33 | < 0.33 | < 0.437 | 0.428 K J | < 0.333 | < 0.349 |
| NEtFOSAA | 2991-50-6 | 0.286 K J | < 0.25 | < 0.24 | < 0.24 | < 0.33 | < 0.31 | < 0.25 | < 0.26 |
| NMeFBSAA | 159381-10-9 | 18.8 | 19.4 | < 0.59 | < 0.58 | 1.94 | 1.89 | 0.29 | 0.262 |
| NMeFHSAA | 715646-50-7 | < 0.58 | < 0.59 | < 0.59 | < 0.58 | < 0.19 | < 0.19 | < 0.19 | < 0.19 |
| PFOSAA | 2806-24-8 | < 0.58 | < 0.59 | < 0.59 | < 0.58 | < 0.19 | < 0.19 | < 0.19 | < 0.19 |
| HFPO-DA | 13252-13-6 | < 0.39 | < 0.39 | < 0.39 | < 0.39 | < 0.51 | < 0.48 | < 0.39 | < 0.41 |
| ADONA | 919005-14-4 | < 1.25 | < 1.26 | < 1.23 | < 1.23 | < 1.64 | < 1.54 | < 1.24 | < 1.31 |
| PFMBA | 863090-89-5 | < 0.11 | < 0.12 | < 0.11 | < 0.11 | < 0.15 | < 0.14 | < 0.11 | < 0.12 |
| NFDHA | 151772-58-6 | < 6.56 | < 6.61 | < 6.47 | < 6.45 | < 8.60 | < 8.10 | < 6.54 | < 6.87 |
| PFMPA | 377-73-1 | < 0.17 | < 0.17 | < 0.17 | < 0.17 | < 0.23 | < 0.21 | < 0.17 | < 0.18 |
| PFMOAA | 674-13-5 | < 5.84 | < 5.93 | < 5.96 | < 5.84 | < 2.28 | < 1.93 | < 1.90 | < 1.90 |
| PFO3OA | 39492-89-2 | < 5.84 | < 5.93 | < 5.96 | < 5.84 | < 1.91 | < 1.93 | < 1.90 | < 1.90 |
| PFO4DA | 39492-90-5 | < 5.84 | < 5.93 | < 5.96 | < 5.84 | < 1.91 | < 1.93 | < 1.90 | < 1.90 |
| PFO5DoDA | 39492-91-6 | < 5.78 | < 5.87 | < 5.91 | < 5.78 | < 2.08 | < 2.29 | < 1.88 | < 1.88 |
| PFO6TeA | N/A | < 5.84 | < 5.93 | < 5.96 | < 5.84 | < 1.91 | < 1.93 | < 1.90 | < 1.90 |
| 9Cl-PF3ONS | 756426-58-1 | < 0.89 | < 0.89 | < 0.87 | < 0.87 | < 1.16 | < 1.10 | < 0.88 | < 0.93 |
| 11Cl-PF3OUdS | 763051-92-9 | < 0.98 | < 0.99 | < 0.97 | < 0.97 | < 1.29 | < 1.21 | < 0.98 | < 1.03 |
| PFEESA | 113507-82-7 | < 0.13 | < 0.14 | < 0.13 | < 0.13 | < 0.18 | < 0.17 | < 0.13 | < 0.14 |
| Nafion BP 1 | 29311-67-9 | < 0.58 | < 0.59 | < 0.59 | < 0.58 | < 0.19 | < 0.19 | < 0.19 | < 0.19 |
| Nafion BP2 | 749836-20-2 | < 0.58 | < 0.59 | < 0.59 | < 0.58 | < 0.19 | < 0.19 | < 0.19 | < 0.19 |
| PFecHS | 133201-07-7 | < 0.75 | < 0.76 | < 0.77 | < 0.75 | < 0.25 | < 0.25 | < 0.24 | < 0.25 |
| PFHxPA | 40143-76-8 | < 7.23 | < 7.33 | < 7.38 | < 7.22 | < 2.37 | < 2.38 | 4.34 | < 2.36 |
| PFOPA | 40143-78-0 | < 7.23 | < 7.33 | < 7.38 | < 7.22 | 6.85 | 7.32 | 5.06 | 5.1 |
| PFDPA | 52299-26-0 | < 7.23 | < 7.33 | < 7.38 | < 7.22 | 8.85 | 7.37 | < 2.35 | < 2.36 |
| 6:2 PAP | 57678-01-0 | < 7.23 | < 7.33 | < 7.38 | < 7.22 | 3.75 | 3.36 | < 2.35 | < 2.36 |
| 8:2 PAP | 57678-03-2 | < 7.34 | < 7.45 | < 7.50 | < 7.34 | 7.86 K | 5.9 K | 2.71 K | 5.45 |
| 6:6 PFPi | 40143-77-9 | < 0.29 | < 0.29 | < 0.30 | < 0.29 | < 0.09 | < 0.10 | < 0.09 | < 0.09 |
| 6:8 PFPi | 610800-34-5 | < 0.29 | < 0.29 | < 0.30 | < 0.29 | < 0.09 | < 0.10 | < 0.09 | < 0.09 |
| 8:8 PFPi | 40143-79-1 | < 0.29 | < 0.29 | < 0.30 | < 0.29 | < 0.09 | < 0.10 | < 0.09 | < 0.09 |
| 6:2 diPAP | 57677-95-9 | < 1.45 | < 1.47 | 6.36 | 6.67 | 57 | 56.5 | < 0.47 | < 0.47 |
| 8:2 diPAP | 678-41-1 | 2.76 | < 1.47 | < 1.48 | < 1.44 | 44.4 K | 41 K | 0.575 K | < 1.09 |
| 4:2 FTOH | 2043-47-2 | 135 | 137 | 181 | 371 | < 170 | < 209 | < 61.6 | < 70.0 |
| 6:2 FTOH | 647-42-7 | 7.12 | < 3.08 | 19.4 | 18.1 | 44.9 | 52.6 | < 4.85 | < 3.48 |
| 7:2 sFTOH | 24015-83-6 | < 3.60 | < 2.48 | < 3.66 | < 13.70 | < 7.19 | < 30.30 | < 12.70 | < 6.69 |
| 8:2 FTOH | 678-39-7 | < 2.01 | < 2.00 | < 4.95 | < 4.28 | < 2.02 | 3.31 | < 2.01 | < 3.04 |
| 10:2 FTOH | 865-86-1 | < 1.00 | < 1.00 | < 1.01 | < 1.00 | 2.05 | 3.77 | < 1.00 | < 1.01 |
| 12:2 FTOH | 39239-77-5 | < 1.00 | < 1.00 | < 1.01 | < 1.00 | < 1.01 | < 1.00 | < 1.00 | < 1.01 |
| ∑84 RPD (%) | | 25.3 | | 2.3 | | 3.5 | | 4.7 | |

All values are in units of ng/L unless otherwise specified. CAS = Chemical Abstracts Service registry number. RPD = relative percent difference. J = The amount detected is below the Reporting Limit/Limit of Quantitation. K = Peak detected but did not meet quantification criteria. Result reported represents the estimated maximum possible concentration.

# Table S3-C: QC Data - Lab Splits

| Abbreviation | CAS Number | Apr-25 | | | | | | | | Sep-25 | | | | | | | |
|---|---|---|---|---|---|---|---|---|---|---|---|---|---|---|---|---|---|
| | | Site A | | Site B | | Site C | | Site D | | Site A | | Site B | | Site C | | Site D | |
| | | Lab 1 | Lab 2 | Lab 1 | Lab 2 | Lab 1 | Lab 2 | Lab 1 | Lab 2 | Lab 1 | Lab 2 | Lab 1 | Lab 2 | Lab 1 | Lab 2 | Lab 1 | Lab 2 |
| PFPrA | 422-64-0 | | | | | | | | | | | | | | | | |
| PFBA | 375-22-4 | 4.72 J | 4.54 | 0.821 J | < 1.63 | 2.13 J | 2.04 J | 1.5 J | < 1.64 | 9.96 | 9.13 | 1.99 J | < 7.63 | 2.14 J | < 1.63 | 1.99 J | < 1.66 |
| PFPeA | 2706-90-3 | 8.62 | 9.53 | < 0.122 | 1.19 I J | 3.4 | 4.25 | 1.21 J | 1.87 I J | 16 | 16.1 | 2.28 J | < 2.00 | 3.27 | 3.45 | 3.17 | < 0.436 |
| PFHxA | 307-24-4 | 8.12 | 8.12 | 0.908 J | 1.4 I J | 3.9 | 4.15 | 2.11 | 2.03 | 12.6 | 11.3 | 5.7 | 4.93 J | 4.36 | 4.04 | 2.59 | 2.28 |
| PFHpA | 375-85-9 | 2.29 | 3.25 | 0.436 J | 0.657 J | 1.35 J | 2.08 | 0.362 J | 0.539 J | 2.51 | 2.98 | 0.962 J | < 1.29 | 1.33 J | 1.76 | 0.655 J | 0.471 J |
| PFOA | 335-67-1 | 17.3 | 18.4 | 2.34 | 2.1 | 5.84 | 6.81 | 0.765 J | < 1.83 | 15.8 | 19.1 | 3.31 | < 8.48 | 4.41 | 5.99 | 0.909 J | < 1.85 |
| PFNA | 375-95-1 | 1.22 J | 1.43 J | < 0.216 | < 0.25 | 0.295 J | 0.414 J | < 0.207 | 0.271 I J | 1.04 J | 1.13 J | 0.554 J | < 1.15 | 0.343 K J | 0.372 J | 0.224 J | < 0.25 |
| PFDA | 335-76-2 | 5.53 | 5.26 | < 0.235 | < 0.43 | 0.367 K J | < 0.44 | < 0.225 | < 0.44 | 9.18 | 9.61 | 0.788 J | < 2.03 | 0.474 K J | < 0.43 | 0.284 J | < 0.44 |
| PFUnA | 2058-94-8 | < 0.140 | < 0.424 | < 0.147 | < 0.42 | 0.187 K J | < 0.42 | < 0.141 | < 0.42 | 0.37 K J | < 0.432 | < 0.136 | < 1.97 | < 0.138 | < 0.42 | < 0.139 | < 0.43 |
| PFDoDA | 307-55-1 | 0.295 K J | 0.265 J | < 0.275 | < 0.23 | < 0.265 | < 0.23 | < 0.263 | < 0.23 | 0.986 J | 1.21 J | 0.26 J | < 1.06 | < 0.257 | < 0.23 | < 0.26 | < 0.23 |
| PFTrDA | 72629-94-8 | < 0.140 | < 0.263 | < 0.147 | < 0.26 | < 0.142 | < 0.26 | < 0.141 | < 0.26 | < 0.141 | < 0.268 | < 0.136 | < 1.22 | < 0.138 | < 0.26 | < 0.139 | < 0.27 |
| PFTeDA | 376-06-7 | < 0.205 | < 0.245 | < 0.216 | < 0.24 | < 0.209 | < 0.25 | < 0.207 | < 0.25 | 0.424 K J | < 0.250 | 0.208 J | < 1.14 | < 0.202 | < 0.24 | < 0.204 | < 0.25 |
| PFHxDA | 67905-19-5 | | | | | | | | | | | | | | | | |
| PFODA | 16517-11-6 | | | | | | | | | | | | | | | | |
| PFMeS | 37181-39-8 | | | | | | | | | | | | | | | | |
| PFEtS | 354-88-1 | | | | | | | | | | | | | | | | |
| PFPrS | 423-41-6 | | | | | | | | | | | | | | | | |
| PFBS | 375-73-5 | 12 | 11.6 | 2.41 | 2.96 | 2.7 | 3.36 | < 0.160 | < 0.69 | 16 | 16.9 | 0.464 J | < 3.22 | 2.25 | 2.31 | < 0.158 | < 0.70 |
| PFPeS | 2706-91-4 | < 0.140 | < 0.497 | < 0.147 | < 0.49 | 0.428 K J | < 0.495 | < 0.141 | < 0.50 | < 0.141 | < 0.51 | 0.187 J | < 2.31 | 0.304 J | < 0.492 | < 0.139 | < 0.50 |
| PFHxS | 355-46-4 | 0.206 J | < 0.517 | < 0.137 | < 0.51 | 1.26 J | 1.46 J | < 0.132 | < 0.52 | 0.248 J | < 0.53 | < 0.127 | < 2.40 | 1.56 J | 1.55 | 0.226 J | < 0.52 |
| PFHpS | 375-92-8 | < 0.130 | < 0.387 | < 0.137 | < 0.39 | 0.181 J | < 0.386 | < 0.132 | < 0.39 | < 0.132 | < 0.39 | < 0.127 | < 1.80 | 0.144 J | < 0.383 | < 0.13 | < 0.39 |
| PFOS | 1763-23-1 | 1.3 J | 2.48 | 0.82 K J | < 1.19 | 5.68 | 8.23 I | 0.68 J | < 1.20 | 2.16 | 3.32 | 1.2 J | < 5.58 | 5.38 | 5.18 | 0.603 J | < 1.22 |
| PFNS | 68259-12-1 | < 0.121 | < 0.62 | < 0.127 | < 0.62 | < 0.123 | < 0.62 | < 0.122 | < 0.62 | < 0.122 | < 0.63 | < 0.118 | < 2.88 | < 0.119 | < 0.61 | < 0.12 | < 0.63 |
| PFDS | 335-77-3 | < 0.102 | < 0.58 | < 0.108 | < 0.58 | < 0.104 | < 0.58 | < 0.103 | < 0.58 | < 0.103 | < 0.59 | < 0.099 | < 2.69 | < 0.101 | < 0.57 | < 0.102 | < 0.59 |
| PFDoS | 79780-39-5 | < 0.158 | < 0.51 | < 0.167 | < 0.51 | < 0.161 | < 0.51 | < 0.160 | < 0.51 | < 0.160 | < 0.52 | < 0.154 | < 2.37 | < 0.156 | < 0.51 | < 0.158 | < 0.52 |
| 3:3 FTCA | 356-02-5 | < 0.67 | < 1.62 | < 0.706 | < 1.61 | < 0.683 | < 1.62 | < 0.676 | < 1.62 | < 0.677 | < 1.65 | < 0.654 | < 7.53 | < 0.661 | < 1.61 | < 0.667 | < 1.64 |
| 5:3 FTCA | 914637-49-3 | 3.76 J | < 6.95 | < 3.83 | < 6.91 | 5.81 J | < 6.93 | < 3.67 | < 6.96 | 5.93 J | 7.98 J | 17.3 J | < 32.30 | < 3.59 | < 6.89 | 7.75 J | 7.29 J |
| 7:3 FTCA | 812-70-4 | < 5.46 | < 3.69 | < 5.76 | < 3.66 | < 5.56 | < 3.67 | < 5.51 | < 3.69 | < 5.52 | < 3.76 | < 5.33 | < 17.10 | < 5.39 | < 3.65 | < 5.44 | < 3.73 |
| 6:2 FTCA | 53826-12-3 | | | | | | | | | | | | | | | | |
| 8:2 FTCA | 27854-31-5 | | | | | | | | | | | | | | | | |
| 10:2 FTCA | 53826-13-4 | | | | | | | | | | | | | | | | |
| 6:2 FTUCA | 70887-88-6 | | | | | | | | | | | | | | | | |
| 8:2 FTUCA | 70887-84-2 | | | | | | | | | | | | | | | | |
| 4:2 FTS | 757124-72-4 | < 0.949 | < 1.30 | < 1.00 | < 1.30 | < 0.967 | < 1.30 | < 0.958 | < 1.31 | < 0.959 | < 1.33 | < 0.927 | < 6.05 | < 0.936 | < 1.29 | < 0.945 | < 1.32 |
| 6:2 FTS | 27619-97-2 | < 5.27 | < 1.25 | < 5.55 | < 1.25 | < 5.37 | < 1.25 | < 5.32 | < 1.25 | < 5.32 | < 1.28 | < 5.14 | < 5.81 | < 5.19 | < 1.24 | < 5.25 | < 1.27 |
| 8:2 FTS | 39108-34-4 | < 0.921 | < 1.92 | < 0.971 | < 1.91 | 1.74 J | < 1.91 | < 0.93 | < 1.92 | < 0.931 | < 1.96 | 1.36 J | < 8.91 | < 0.909 | < 1.90 | < 0.918 | < 1.95 |
| 10:2 FTS | 120226-60-0 | | | | | | | | | | | | | | | | |
| NMeFOSE | 24448-09-7 | < 2.09 | < 2.67 | < 2.21 | < 2.65 | < 2.13 | < 2.66 | < 2.11 | 3.12 J | < 2.12 | < 2.72 | 4.32 J | < 12.40 | < 2.06 | < 2.64 | < 2.09 | 5.22 J |
| NEtFOSE | 1691-99-2 | < 2.05 | < 2.62 | < 2.65 | < 2.60 | < 2.56 | < 2.61 | < 2.54 | < 2.62 | < 2.54 | < 2.67 | < 2.45 | < 12.20 | < 2.48 | < 2.59 | < 2.5 | < 2.65 |
| PFOSA | 754-91-6 | 0.615 J | 1.04 J | < 0.432 | < 0.41 | < 0.417 | < 0.41 | < 0.413 | < 0.41 | < 0.414 | 0.869 J | < 0.40 | < 1.90 | < 0.404 | < 0.41 | < 0.408 | < 0.41 |
| NMeFOSA | 31506-32-8 | < 0.186 | < 1.04 | < 0.196 | < 1.03 | < 0.19 | < 1.03 | < 0.188 | < 1.04 | < 0.188 | < 1.06 | 0.2 K J | < 4.81 | < 0.184 | < 1.03 | < 0.185 | < 1.05 |
| NEtFOSA | 4151-50-2 | < 0.54 | < 1.00 | < 0.569 | < 0.99 | < 0.55 | < 1.00 | < 0.545 | < 1.00 | < 0.545 | < 1.02 | < 0.527 | < 4.63 | < 0.532 | < 0.99 | < 0.538 | < 1.01 |
| PFBSA | 30334-69-1 | | | | | | | | | | | | | | | | |
| PFHxSA | 41997-13-1 | | | | | | | | | | | | | | | | |
| PFHpSA | 82765-77-3 | | | | | | | | | | | | | | | | |
| PFDSA | 4262-70-8 | | | | | | | | | | | | | | | | |
| NMeFBSA | 68298-12-4 | | | | | | | | | | | | | | | | |
| NMeFOSAA | 2355-31-9 | < 0.326 | < 0.709 | < 0.343 | < 0.71 | < 0.332 | < 0.71 | < 0.329 | < 0.710 | 0.414 J | 1.16 J | 0.329 J | < 3.29 | < 0.321 | < 0.703 | < 0.324 | < 0.719 |
| NEtFOSAA | 2991-50-6 | < 0.242 | < 0.707 | < 0.255 | < 0.70 | < 0.246 | < 0.71 | < 0.244 | < 0.708 | 1.12 J | 1.33 J | < 0.236 | < 3.28 | < 0.239 | < 0.701 | < 0.241 | < 0.717 |
| NMeFBSAA | 159381-10-9 | | | | | | | | | | | | | | | | |
| NMeFHSAA | 715646-50-7 | | | | | | | | | | | | | | | | |
| PFOSAA | 2806-24-8 | | | | | | | | | | | | | | | | |
| HFPO-DA | 13252-13-6 | < 0.382 | < 1.75 | < 0.402 | < 1.74 | < 0.389 | < 1.74 | < 0.385 | < 1.75 | < 0.386 | < 1.78 | < 0.372 | < 8.10 | < 0.376 | < 1.73 | < 0.38 | < 1.77 |
| ADONA | 919005-14-4 | < 1.22 | < 1.63 | < 1.28 | < 1.62 | < 1.24 | < 1.63 | < 1.23 | < 1.63 | < 1.23 | < 1.66 | < 1.19 | < 7.58 | < 1.2 | < 1.62 | < 1.21 | < 1.65 |
| PFMBA | 863090-89-5 | < 0.112 | < 0.46 | < 0.118 | < 0.46 | < 0.114 | < 0.46 | < 0.113 | < 0.46 | < 0.113 | < 0.47 | < 0.109 | < 2.15 | < 0.11 | < 0.46 | < 0.11 | < 0.47 |
| NFDHA | 151772-58-6 | < 6.40 | < 1.59 | < 6.75 | < 1.58 | < 6.52 | < 1.59 | < 6.46 | < 1.59 | < 6.47 | < 1.62 | < 6.25 | < 7.39 | < 6.31 | < 1.58 | < 6.38 | < 1.61 |
| PFMPA | 377-73-1 | < 0.168 | < 0.70 | < 0.177 | < 0.70 | < 0.171 | < 0.70 | < 0.169 | < 0.70 | < 0.169 | < 0.71 | < 0.164 | < 3.26 | < 0.165 | < 0.70 | < 0.167 | < 0.71 |
| PFMOAA | 674-13-5 | | | | | | | | | | | | | | | | |

# Table S3-C: QC Data - Lab Splits (cont'd)

| Abbreviation | CAS Number | Apr-25 | | | | | | | | Sep-25 | | | | | | | |
|---|---|---|---|---|---|---|---|---|---|---|---|---|---|---|---|---|---|
| | | Site A | | Site B | | Site C | | Site D | | Site A | | Site B | | Site C | | Site D | |
| | | Lab 1 | Lab 2 | Lab 1 | Lab 2 | Lab 1 | Lab 2 | Lab 1 | Lab 2 | Lab 1 | Lab 2 | Lab 1 | Lab 2 | Lab 1 | Lab 2 | Lab 1 | Lab 2 |
| PFO3OA | 39492-89-2 | | | | | | | | | | | | | | | | |
| PFO4DA | 39492-90-5 | | | | | | | | | | | | | | | | |
| PFO5DoDA | 39492-91-6 | | | | | | | | | | | | | | | | |
| PFO6TeA | N/A | | | | | | | | | | | | | | | | |
| 9Cl-PF3ONS | 756426-58-1 | < 0.865 | < 1.97 | < 0.912 | < 1.96 | < 0.882 | < 1.96 | < 0.874 | < 1.97 | < 0.875 | < 2.01 | < 0.845 | < 9.15 | < 0.853 | < 1.95 | < 0.862 | < 2.00 |
| 11Cl-PF3OUdS | 763051-92-9 | < 0.959 | < 1.98 | < 1.01 | < 1.97 | < 0.976 | < 1.97 | < 0.968 | < 1.98 | < 0.969 | < 2.02 | < 0.936 | < 9.20 | < 0.945 | < 1.96 | < 0.955 | < 2.01 |
| PFEESA | 113507-82-7 | < 0.130 | < 0.38 | < 0.137 | < 0.38 | < 0.133 | < 0.38 | < 0.132 | < 0.38 | < 0.132 | < 0.389 | < 0.127 | < 1.77 | < 0.128 | < 0.38 | < 0.13 | < 0.387 |
| Nafion BP 1 | 29311-67-9 | | | | | | | | | | | | | | | | |
| Nafion BP2 | 749836-20-2 | | | | | | | | | | | | | | | | |
| PFecHS | 133201-07-7 | | | | | | | | | | | | | | | | |
| PFHxPA | 40143-76-8 | | | | | | | | | | | | | | | | |
| PFOPA | 40143-78-0 | | | | | | | | | | | | | | | | |
| PFDPA | 52299-26-0 | | | | | | | | | | | | | | | | |
| 6:2 PAP | 57678-01-0 | | | | | | | | | | | | | | | | |
| 8:2 PAP | 57678-03-2 | | | | | | | | | | | | | | | | |
| 6:6 PFPi | 40143-77-9 | | | | | | | | | | | | | | | | |
| 6:8 PFPi | 610800-34-5 | | | | | | | | | | | | | | | | |
| 8:8 PFPi | 40143-79-1 | | | | | | | | | | | | | | | | |
| 6:2 diPAP | 57677-95-9 | | | | | | | | | | | | | | | | |
| 8:2 diPAP | 678-41-1 | | | | | | | | | | | | | | | | |
| 4:2 FTOH | 2043-47-2 | | | | | | | | | | | | | | | | |
| 6:2 FTOH | 647-42-7 | | | | | | | | | | | | | | | | |
| 7:2 sFTOH | 24015-83-6 | | | | | | | | | | | | | | | | |
| 8:2 FTOH | 678-39-7 | | | | | | | | | | | | | | | | |
| 10:2 FTOH | 865-86-1 | | | | | | | | | | | | | | | | |
| 12:2 FTOH | 39239-77-5 | | | | | | | | | | | | | | | | |
| ∑40 RPD (%) | | 0.1 | | 7.1 | | 7.3 | | 16.6 | | 7.5 | | 157 | | 5.2 | | 18.7 | |

All values are in units of ng/L. CAS = Chemical Abstracts Service registry number. J = The amount detected is below the Reporting Limit/Limit of Quantitation. K = Peak detected but did not meet quantification criteria. Result reported represents the estimated maximum possible concentration. I = Ion transition ratio is outside of the acceptance criteria.

# Table S3-D: PFAS Data - Site A

| Abbreviation | CAS Number | Oct-24 | Nov-24 | | Dec-24 | Jan-25 | Feb-25 | | Mar-25 | | Apr-25 | May-25 | | Jun-25 | | Jul-25 | Aug-25 | | Sep-25 |
|---|---|---|---|---|---|---|---|---|---|---|---|---|---|---|---|---|---|---|---|---|
| | | Effluent | Effluent | Effluent (post-TOP) | Effluent | Effluent | Effluent | Effluent (post-TOP) | Influent | Effluent | Effluent | Effluent | Effluent (post-TOP) | Influent | Effluent | Effluent | Effluent | Effluent (post-TOP) | Effluent |
| PFPrA | 422-64-0 | | < 18.7 | | | | < 19.6 | | | | | < 19.7 | | | | | < 19.1 | | |
| PFBA | 375-22-4 | 12.7 | 8.49 | 72.5 | 5.84 J | 4.71 J | 4.2 J | 52.3 | < 1.64 | 3.32 J | 4.54 | 6.3 | 41.6 J | 3.44 K J | 4.59 J | 6.33 J | 8.59 | 99.4 | 9.13 |
| PFPeA | 2706-90-3 | 40.7 | 25.2 | 46.9 | 18.7 | 16.3 | 10.6 | 31.5 | 1.29 I J | 7.1 | 9.53 | 7.33 | 21.9 J | < 1.19 | 15.3 | 15.3 | 15.5 | 47.3 | 16.1 |
| PFHxA | 307-24-4 | 32.3 | 14.8 | 27.7 | 13 | 10.5 | 7.8 | 20.3 | 1.34 J | 5.9 | 8.12 | 7.63 | 19.7 | 2.07 | 10.5 | 11.5 | 11.8 | 34.7 | 11.3 |
| PFHpA | 375-85-9 | 8.2 | 4.16 | 14.1 | 3.75 | 3.4 | 2.01 | 9.07 J | 0.432 J | 1.68 | 3.25 | 1.8 | 8.48 J | 0.489 J | 2.03 | 2.77 | 2.53 | 24.4 | 2.98 |
| PFOA | 335-67-1 | 77.4 | 39.3 | 51.9 | 27.9 | 30.8 | 19.1 | 31 | < 1.83 | 12.5 | 18.4 | 13.6 | 22.4 | 2.46 | 17.2 | 23.4 | 17.3 | 34.5 | 19.1 |
| PFNA | 375-95-1 | 2.72 | 1.67 | 5.77 J | 1.35 J | 1.14 J | 0.941 J | 3.94 J | < 0.25 | 1.1 J | 1.43 J | 1.06 J | 3.73 J | 0.305 K J | 0.86 J | 1.4 J | 1.02 J | 10.6 J | 1.13 J |
| PFDA | 335-76-2 | 17 | 10.8 | 14.2 | 7.64 | 6.76 | 6.98 | 10.8 J | < 0.44 | 7.19 | 5.26 | 6.05 | 7.48 J | 0.724 J | 7.72 | 9.88 | 8.73 | 14.8 | 9.61 |
| PFUnA | 2058-94-8 | < 0.44 | < 0.14 | < 2.98 | < 0.426 | < 0.423 | 0.173 J | < 3.12 | < 0.42 | < 0.428 | < 0.424 | 0.216 K J | < 3.03 | < 0.144 | 0.338 K J | < 0.431 | 0.309 K J | 4.8 K J | < 0.432 |
| PFDoDA | 307-55-1 | < 0.24 | 0.682 J | < 2.38 | 0.582 J | 0.416 J | 0.422 J | < 2.49 | < 0.23 | 2.59 | 0.265 J | 0.319 J | < 2.43 | < 0.268 | 0.954 J | 1.09 J | 0.929 J | < 2.38 | 1.21 J |
| PFTrDA | 72629-94-8 | < 0.27 | < 0.14 | < 2.98 | < 0.264 | < 0.262 | < 0.14 | < 3.12 | < 0.26 | 0.296 J | < 0.263 | < 0.143 | < 3.03 | < 0.14 | < 0.14 | < 0.267 | < 0.14 | < 2.98 | < 0.268 |
| PFTeDA | 376-06-7 | < 0.25 | < 0.21 | < 2.98 | < 0.247 | < 0.245 | < 0.21 | < 3.12 | < 0.25 | 0.875 I J | < 0.245 | < 0.21 | < 3.03 | < 0.21 | < 0.21 | < 0.249 | 0.233 J | < 2.98 | < 0.250 |
| PFHxDA | 67905-19-5 | | < 2.53 | | | | < 2.38 | | | | | < 7.23 | | | | | < 2.36 | | |
| PFODA | 16517-11-6 | | < 2.57 | | | | < 2.42 | | | | | < 7.34 | | | | | < 2.40 | | |
| PFMeS | 37181-39-8 | | 24.4 | | | | < 9.79 | | | | | < 9.83 | | | | | < 9.54 | | |
| PFEtS | 354-88-1 | | < 4.81 | | | | < 4.92 | | | | | < 4.94 | | | | | < 4.91 | | |
| PFPrS | 423-41-6 | | < 4.76 | | | | < 4.91 | | | | | < 4.93 | | | | | < 4.86 | | |
| PFBS | 375-73-5 | 33.1 | 27.4 | 27.4 | 16.2 | 9.47 | 8.62 | 9.15 J | < 0.69 | 9.87 | 11.6 | 9.2 | 7.34 J | 3.04 | 10.9 | 12.2 | 12.2 | 11 J | 16.9 |
| PFPeS | 2706-91-4 | < 0.51 | < 0.14 | < 2.98 | < 0.50 | < 0.50 | < 0.144 | < 3.12 | < 0.50 | < 0.50 | < 0.497 | < 0.143 | < 3.03 | < 0.144 | < 0.143 | < 0.51 | < 0.14 | < 2.98 | < 0.51 |
| PFHxS | 355-46-4 | < 0.53 | < 0.13 | < 3.43 | < 0.52 | < 0.52 | 0.192 J | < 3.58 | < 0.52 | < 0.52 | < 0.517 | 0.196 J | < 3.49 | < 0.134 | 0.262 J | < 0.53 | 0.156 K J | < 3.42 | < 0.53 |
| PFHpS | 375-92-8 | < 0.40 | < 0.13 | < 2.98 | < 0.39 | < 0.39 | < 0.135 | < 3.12 | < 0.39 | < 0.39 | < 0.387 | < 0.134 | < 3.03 | < 0.134 | < 0.133 | < 0.39 | < 0.13 | < 2.98 | < 0.39 |
| PFOS | 1763-23-1 | 2.72 | 2.38 | < 2.98 | 1.49 J | 1.25 J | 0.92 J | < 3.12 | < 1.20 | < 1.21 | 2.48 | 1.57 | < 3.03 | < 0.32 | 1.59 | 1.98 | 1.98 | < 2.98 | 3.32 |
| PFNS | 68259-12-1 | < 0.64 | < 0.12 | < 2.98 | < 0.62 | < 0.62 | < 0.13 | < 3.12 | < 0.62 | < 0.63 | < 0.62 | < 0.12 | < 3.03 | < 0.12 | < 0.12 | < 0.63 | < 0.12 | < 2.98 | < 0.63 |
| PFDS | 335-77-3 | < 0.60 | < 0.10 | < 2.98 | < 0.58 | < 0.58 | < 0.11 | < 3.12 | < 0.58 | < 0.59 | < 0.58 | < 0.11 | < 3.03 | < 0.11 | < 0.11 | 1.06 I J | < 0.10 | < 2.98 | < 0.59 |
| PFDoS | 79780-39-5 | < 0.53 | < 0.16 | < 2.98 | < 0.51 | < 0.51 | < 0.16 | < 3.12 | < 0.51 | < 0.52 | < 0.51 | < 0.16 | < 3.03 | < 0.16 | < 0.16 | < 0.518 | < 0.16 | < 2.98 | < 0.52 |
| 3:3 FTCA | 356-02-5 | < 1.67 | < 0.67 | < 11.90 | < 1.63 | < 1.62 | < 0.69 | < 12.50 | < 1.62 | < 1.64 | < 1.62 | < 0.69 | < 12.10 | < 0.69 | < 0.69 | < 1.65 | < 0.67 | < 11.90 | < 1.65 |
| 5:3 FTCA | 914637-49-3 | < 7.15 | < 3.65 | < 74.50 | < 6.99 | < 6.93 | < 3.76 | < 77.90 | < 6.95 | < 7.02 | < 6.95 | 4.16 J | < 75.80 | 11.2 J | 6.33 J | 7.85 J | 5.34 J | < 74.40 | 7.98 J |
| 7:3 FTCA | 812-70-4 | < 3.79 | < 5.49 | < 74.50 | < 3.71 | < 3.68 | < 5.64 | < 77.90 | < 3.69 | 3.88 J | < 3.69 | < 5.6 | < 75.80 | < 5.62 | < 5.59 | < 3.74 | < 5.48 | < 74.40 | < 3.76 |
| 6:2 FTCA | 53826-12-3 | | < 5.10 | | | | < 4.8 | | | | | < 14.6 | | | | | < 4.76 | | |
| 8:2 FTCA | 27854-31-5 | | < 5.10 | | | | < 4.8 | | | | | < 14.6 | | | | | < 4.76 | | |
| 10:2 FTCA | 53826-13-4 | | < 5.10 | | | | < 4.80 | | | | | < 14.60 | | | | | < 4.76 | | |
| 6:2 FTUCA | 70887-88-6 | | < 1.01 | | | | < 0.95 | | | | | < 2.89 | | | | | < 0.95 | | |
| 8:2 FTUCA | 70887-84-2 | | < 0.51 | | | | < 0.48 | | | | | < 1.45 | | | | | < 0.47 | | |
| 4:2 FTS | 757124-72-4 | < 1.34 | < 0.95 | < 11.90 | < 1.31 | < 1.30 | < 0.98 | < 12.50 | < 1.30 | < 1.32 | < 1.30 | < 0.97 | 12.1 | < 0.98 | < 0.97 | < 1.32 | < 0.95 | < 11.90 | < 1.33 |
| 6:2 FTS | 27619-97-2 | < 1.29 | < 5.29 | < 10.70 | < 1.26 | < 1.25 | < 5.44 | < 11.20 | < 1.25 | < 1.27 | < 1.25 | < 5.40 | 10.9 | < 5.42 | < 5.39 | < 1.27 | < 5.29 | < 10.70 | < 1.28 |
| 8:2 FTS | 39108-34-4 | < 1.97 | < 0.93 | < 10.10 | < 1.93 | < 1.92 | < 0.95 | < 10.60 | < 1.92 | < 1.94 | < 1.92 | < 0.94 | 10.3 | < 0.95 | < 0.94 | < 1.95 | < 0.92 | < 10.10 | < 1.96 |
| 10:2 FTS | 120226-60-0 | | < 1.01 | | | | < 0.951 | | | | | < 2.89 | | | | | < 0.945 | | |
| NMeFOSE | 24448-09-7 | < 2.74 | < 2.10 | < 29.80 | < 2.68 | < 2.66 | < 2.16 | < 31.20 | < 2.67 | < 2.70 | < 2.67 | < 2.15 | 30.3 | < 2.15 | < 2.14 | < 2.71 | < 2.1 | < 29.80 | < 2.72 |
| NEtFOSE | 1691-99-2 | < 2.69 | < 2.52 | < 29.80 | < 2.63 | < 2.61 | < 2.60 | < 31.20 | < 2.62 | < 2.64 | < 2.62 | < 2.58 | 30.3 | < 2.59 | < 2.57 | < 2.66 | < 2.52 | < 29.80 | < 2.67 |
| PFOSA | 754-91-6 | < 0.42 | 0.474 J | < 2.98 | 0.764 I J | 2.83 | < 0.42 | < 3.12 | < 0.41 | 0.469 I J | 1.04 J | 0.648 J | < 3.03 | < 0.42 | 0.842 J | 2.26 | 0.455 J | < 2.98 | 0.869 J |
| NMeFOSA | 31506-32-8 | < 1.07 | < 0.19 | < 2.98 | < 1.04 | < 1.03 | < 0.19 | < 3.12 | < 1.04 | < 1.05 | < 1.04 | < 0.19 | < 3.03 | < 0.19 | < 0.19 | < 1.05 | < 0.19 | < 2.98 | < 1.06 |
| NEtFOSA | 4151-50-2 | < 1.03 | < 0.54 | < 8.34 | < 1.00 | < 1.00 | < 0.56 | < 8.73 | < 1.00 | < 1.01 | < 1.00 | < 0.55 | < 8.49 | < 0.56 | < 0.55 | < 1.01 | < 0.54 | < 8.33 | < 1.02 |
| PFBSA | 30334-69-1 | | 7.27 | | | | 2.71 | | | | | 3.03 | | | | | 4.6 K | | |
| PFHxSA | 41997-13-1 | | < 0.10 | | | | < 0.10 | | | | | < 0.29 | | | | | < 0.09 | | |
| PFHpSA | 82765-77-3 | | < 0.10 | | | | < 0.10 | | | | | < 0.29 | | | | | < 0.09 | | |
| PFDSA | 4262-70-8 | | < 0.10 | | | | < 0.10 | | | | | < 0.29 | | | | | < 0.09 | | |
| NMeFBSA | 68298-12-4 | | 1.3 | | | | 1.71 | | | | | 1.53 K | | | | | 1.75 | | |
| NMeFOSAA | 2355-31-9 | < 0.73 | < 0.33 | < 2.98 | 0.729 J | 0.956 J | 0.341 K J | < 3.12 | < 0.71 | < 0.72 | < 0.709 | < 0.334 | < 3.03 | < 0.335 | 0.502 J | 1.08 J | 0.877 J | < 2.98 | 1.16 J |
| NEtFOSAA | 2991-50-6 | < 0.73 | < 0.24 | < 2.98 | < 0.711 | < 0.706 | < 0.25 | < 3.12 | < 0.71 | < 0.72 | < 0.707 | 0.286 K J | < 3.03 | < 0.249 | 0.617 K J | < 0.718 | 0.331 J | < 2.98 | 1.33 J |
| NMeFBSAA | 159381-10-9 | | 27.3 | | | | 24.7 | | | | | 18.8 | | | | | 40.8 | | |
| NMeFHSAA | 715646-50-7 | | < 0.20 | | | | < 0.19 | | | | | < 0.58 | | | | | < 0.19 | | |
| PFOSAA | 2806-24-8 | | < 0.20 | | | | < 0.19 | | | | | < 0.58 | | | | | < 0.19 | | |
| HFPO-DA | 13252-13-6 | < 1.79 | < 0.38 | | < 1.76 | < 1.74 | < 0.39 | | < 1.75 | < 1.76 | < 1.75 | < 0.39 | | < 0.39 | < 0.39 | < 1.77 | < 0.38 | | < 1.78 |
| ADONA | 919005-14-4 | < 1.68 | < 1.22 | | < 1.64 | < 1.63 | < 1.26 | | < 1.63 | < 1.65 | < 1.63 | < 1.25 | | < 1.25 | < 1.25 | < 1.66 | < 1.22 | | < 1.66 |
| PFMBA | 863090-89-5 | < 0.48 | < 0.11 | | < 0.47 | < 0.46 | < 0.12 | | < 0.46 | < 0.47 | < 0.46 | < 0.11 | | < 0.12 | < 0.11 | < 0.47 | < 0.11 | | < 0.47 |
| NFDHA | 151772-58-6 | < 1.64 | < 6.43 | | < 1.60 | < 1.59 | < 6.61 | | < 1.59 | < 1.61 | < 1.59 | < 6.56 | | < 6.59 | < 6.55 | < 1.62 | < 6.42 | | < 1.62 |
| PFMPA | 377-73-1 | < 0.72 | < 0.17 | | < 0.71 | < 0.70 | < 0.17 | | < 0.70 | < 0.71 | < 0.70 | < 0.17 | | < 0.17 | < 0.17 | < 0.71 | < 0.17 | | < 0.71 |

# Table S3-D: PFAS Data - Site A (cont'd)

| Abbreviation | CAS Number | Oct-24 | Nov-24 | | Dec-24 | Jan-25 | Feb-25 | | Mar-25 | | Apr-25 | May-25 | | Jun-25 | | Jul-25 | Aug-25 | | Sep-25 |
|---|---|---|---|---|---|---|---|---|---|---|---|---|---|---|---|---|---|---|---|
| | | Effluent | Effluent | Effluent (post-TOP) | Effluent | Effluent | Effluent | Effluent (post-TOP) | Influent | Effluent | Effluent | Effluent | Effluent (post-TOP) | Influent | Effluent | Effluent | Effluent | Effluent (post-TOP) | Effluent |
| PFMOAA | 674-13-5 | | < 2.05 | | | | < 1.92 | | | | | < 5.84 | | | | | < 1.91 | | |
| PFO3OA | 39492-89-2 | | < 2.05 | | | | < 1.92 | | | | | < 5.84 | | | | | < 1.91 | | |
| PFO4DA | 39492-90-5 | | < 2.05 | | | | < 1.92 | | | | | < 5.84 | | | | | < 1.91 | | |
| PFO5DoDA | 39492-91-6 | | < 2.03 | | | | < 1.90 | | | | | < 5.78 | | | | | < 1.89 | | |
| PFO6TeA | N/A | | < 2.05 | | | | < 1.92 | | | | | < 5.84 | | | | | < 1.91 | | |
| 9Cl-PF3ONS | 756426-58-1 | < 2.03 | < 0.87 | | < 1.98 | < 1.97 | < 0.89 | | < 1.97 | < 1.99 | < 1.97 | < 0.89 | | < 0.89 | < 0.89 | < 2.00 | < 0.87 | | < 2.01 |
| 11Cl-PF3OUdS | 763051-92-9 | < 2.04 | < 0.96 | | < 1.99 | < 1.98 | < 0.99 | | < 1.98 | < 2.00 | < 1.98 | < 0.98 | | < 0.99 | < 0.98 | < 2.01 | < 0.96 | | < 2.02 |
| PFEESA | 113507-82-7 | < 0.39 | < 0.13 | | < 0.38 | < 0.38 | < 0.14 | | < 0.38 | < 0.39 | < 0.38 | < 0.13 | | < 0.13 | < 0.13 | < 0.39 | < 0.13 | | < 0.389 |
| Nafion BP 1 | 29311-67-9 | | < 0.20 | | | | < 0.19 | | | | | < 0.578 | | | | | < 0.189 | | |
| Nafion BP2 | 749836-20-2 | | < 0.20 | | | | < 0.19 | | | | | < 0.58 | | | | | < 0.189 | | |
| PFecHS | 133201-07-7 | | < 0.26 | | | | < 0.247 | | | | | < 0.75 | | | | | < 0.246 | | |
| PFHxPA | 40143-76-8 | | < 2.53 | | | | 2.49 | | | | | < 7.23 | | | | | < 2.36 | | |
| PFOPA | 40143-78-0 | | < 2.53 | | | | < 2.38 | | | | | < 7.23 | | | | | 4.01 | | |
| PFDPA | 52299-26-0 | | < 2.53 | | | | < 2.38 | | | | | < 7.23 | | | | | < 2.36 | | |
| 6:2 PAP | 57678-01-0 | | < 2.53 | | | | < 2.38 | | | | | < 7.23 | | | | | 2.76 | | |
| 8:2 PAP | 57678-03-2 | | < 2.57 | | | | < 2.42 | | | | | < 7.34 | | | | | 3.96 K | | |
| 6:6 PFPi | 40143-77-9 | | < 0.10 | | | | < 0.10 | | | | | < 0.29 | | | | | < 0.09 | | |
| 6:8 PFPi | 610800-34-5 | | < 0.10 | | | | < 0.10 | | | | | < 0.29 | | | | | < 0.09 | | |
| 8:8 PFPi | 40143-79-1 | | < 0.10 | | | | < 0.10 | | | | | < 0.29 | | | | | < 0.09 | | |
| 6:2 diPAP | 57677-95-9 | | < 0.51 | | | | < 0.48 | | | | | < 1.45 | | | | | < 0.47 | | |
| 8:2 diPAP | 678-41-1 | | 3.51 | | | | 2.18 K | | | | | 2.76 | | | | | 12.1 | | |
| 4:2 FTOH | 2043-47-2 | | < 75.7 | | | | < 26.9 | | | | | < 135 | | | | | < 157 | | |
| 6:2 FTOH | 647-42-7 | | 8.4 | | | | < 2.01 | | | | | 7.12 | | | | | < 2.14 | | |
| 7:2 sFTOH | 24015-83-6 | | < 17.5 | | | | < 2.01 | | | | | < 3.60 | | | | | < 3.94 | | |
| 8:2 FTOH | 678-39-7 | | < 3.63 | | | | < 2.01 | | | | | < 2.01 | | | | | < 2.00 | | |
| 10:2 FTOH | 865-86-1 | | < 1.28 | | | | < 1.00 | | | | | < 1.00 | | | | | < 1.00 | | |
| 12:2 FTOH | 39239-77-5 | | < 1.28 | | | | < 1.00 | | | | | < 1.00 | | | | | < 1.00 | | |
| AOF (µg/L) | N/A | | 1.85 | | | | < 1.44 | | | | | < 1.45 | | | | | < 2.0 A | | |

All values are in units of ng/L. CAS = Chemical Abstracts Service registry number. J = The amount detected is below the Reporting Limit/Limit of Quantitation. K = Peak detected but did not meet quantification criteria. Result reported represents the estimated maximum possible concentration. I = Ion transition ratio is outside of the acceptance criteria. A = Sample analyzed out of hold time.

# Table S3-E: PFAS Data - Site B

| Abbreviation | CAS Number | Oct-24 | Nov-24 | | Dec-24 | Jan-25 | Feb-25 | | Mar-25 | Apr-25 | May-25 | | Jun-25 | Jul-25 | Aug-25 | | Sep-25 |
|---|---|---|---|---|---|---|---|---|---|---|---|---|---|---|---|---|---|
| | | Effluent | Effluent | Effluent (post-TOP) | Effluent | Effluent | Effluent | Effluent (post-TOP) | Effluent | Effluent | Effluent | Effluent (post-TOP) | Effluent | Effluent | Effluent | Effluent (post-TOP) | Effluent |
| PFPrA | 422-64-0 | | < 19.6 | | | | < 19.4 | | | | < 19.5 | | | | < 19.3 | | |
| PFBA | 375-22-4 | < 1.67 | < 0.399 | 49.7 | < 1.64 | < 1.76 | < 0.41 | 56.4 | < 1.63 | < 1.63 | 0.467 J | 21.2 J | < 6.42 | 4.38 J | 2.84 J | 101 | < 7.63 |
| PFPeA | 2706-90-3 | 4.67 | 5.03 | 58.9 | 4.84 I | < 0.46 | < 1.17 | 83.7 | < 0.43 | 1.19 I J | < 1.17 | 28.1 | 1.06 I J | 5.37 | 2.56 J | 131 | < 2.00 |
| PFHxA | 307-24-4 | 8.23 | 5.01 | 29.3 | 5.19 | 2.34 | 2.16 | 48.1 | 2.45 | 1.4 I J | 0.841 J | 18.7 | 1.7 | 7.92 | 4.91 | 66.2 | 4.93 J |
| PFHpA | 375-85-9 | < 0.283 | < 0.25 | 14.8 | 0.571 J | 0.367 J | < 0.26 | 29.7 | 0.429 J | 0.657 J | 0.336 K J | 12.7 | 0.531 J | 1.46 J | 0.876 J | 44.6 | < 1.29 |
| PFOA | 335-67-1 | 3.95 | 1.8 | 18 | < 1.83 | < 1.96 | 1.39 J | 27.7 | < 1.81 | 2.1 | 1.03 J | < 2.98 | 2.02 | 6.26 | 2.5 | 32.7 | < 8.48 |
| PFNA | 375-95-1 | < 0.25 | < 0.204 | 5.94 J | 0.279 J | < 0.27 | < 0.21 | 12 J | 0.324 I J | < 0.25 | < 0.21 | 3.5 J | < 1.61 | 0.6 J | 0.429 K J | 17.1 | < 1.15 |
| PFDA | 335-76-2 | < 0.44 | 0.841 K J | 4.4 J | 0.579 J | < 0.47 | 0.367 J | 7.81 J | 0.448 J | < 0.43 | 0.263 J | < 2.98 | 0.497 J | 0.913 J | 1.05 J | 12.2 | < 2.03 |
| PFUnA | 2058-94-8 | < 0.43 | < 0.14 | < 2.83 | < 0.42 | < 0.45 | < 0.14 | 3.66 J | < 0.42 | < 0.42 | 0.142 K J | 5.95 K J | < 1.61 | < 0.48 | 0.429 K J | 5.75 J | < 1.97 |
| PFDoDA | 307-55-1 | < 0.23 | < 0.26 | < 2.27 | < 0.23 | < 0.25 | < 0.26 | 2.72 K J | < 0.23 | < 0.23 | < 0.26 | < 2.38 | < 1.61 | < 0.26 | 0.456 K J | 4.74 J | < 1.06 |
| PFTrDA | 72629-94-8 | < 0.27 | < 0.14 | < 2.83 | < 0.26 | < 0.28 | < 0.14 | < 3.06 | < 0.26 | < 0.26 | < 0.14 | < 2.98 | < 1.61 | < 0.30 | < 0.19 | < 3.00 | < 1.22 |
| PFTeDA | 376-06-7 | < 0.25 | < 0.20 | < 2.83 | < 0.25 | < 0.26 | < 0.21 | < 3.06 | < 0.24 | < 0.24 | < 0.21 | < 2.98 | < 1.61 | < 0.28 | < 0.28 | 3.52 J | < 1.14 |
| PFHxDA | 67905-19-5 | | < 2.32 | | | | < 2.35 | | | | < 7.23 | | | | < 2.37 | | |
| PFODA | 16517-11-6 | | < 2.36 | | | | < 2.39 | | | | < 7.35 | | | | < 2.41 | | |
| PFMeS | 37181-39-8 | | < 9.82 | | | | < 9.69 | | | | < 9.75 | | | | < 9.66 | | |
| PFEtS | 354-88-1 | | < 5.05 | | | | < 4.87 | | | | < 4.90 | | | | < 4.97 | | |
| PFPrS | 423-41-6 | | < 5.00 | | | | < 4.86 | | | | < 4.89 | | | | < 4.92 | | |
| PFBS | 375-73-5 | < 0.71 | 0.682 J | < 2.83 | 6.47 I | < 0.74 | 1.09 K J | < 3.06 | 4.66 I | 2.96 | 18.5 | 20.8 | 4.33 I | 1.99 I | < 0.21 | < 3.00 | < 3.22 |
| PFPeS | 2706-91-4 | < 0.51 | < 0.14 | < 2.83 | 1.39 I J | < 0.53 | < 0.14 | < 3.06 | < 0.49 | < 0.49 | < 0.14 | < 2.98 | < 1.51 | < 0.56 | < 0.19 | < 3.00 | < 2.31 |
| PFHxS | 355-46-4 | < 0.53 | < 0.13 | < 3.26 | < 0.52 | < 0.55 | < 0.13 | < 3.52 | < 0.51 | < 0.51 | < 0.13 | < 3.42 | < 1.47 | < 0.58 | 0.317 J | < 3.45 | < 2.40 |
| PFHpS | 375-92-8 | < 0.39 | < 0.13 | < 2.83 | < 0.39 | < 0.41 | < 0.13 | < 3.06 | < 0.38 | < 0.39 | < 0.13 | < 2.98 | < 1.53 | < 0.43 | < 0.18 | < 3.00 | < 1.80 |
| PFOS | 1763-23-1 | < 1.22 | 1.84 K | < 2.83 | < 1.20 | < 1.29 | < 0.31 | < 3.06 | < 1.19 | < 1.19 | 0.492 J | < 2.98 | 1.6 I | 2.23 I | 1.63 J | 3.35 J | < 5.58 |
| PFNS | 68259-12-1 | < 0.63 | < 0.12 | < 2.83 | < 0.62 | < 0.66 | < 0.12 | < 3.06 | < 0.62 | < 0.62 | < 0.12 | < 2.98 | < 1.55 | < 0.70 | < 0.16 | < 3.00 | < 2.88 |
| PFDS | 335-77-3 | < 0.59 | < 0.10 | < 2.83 | < 0.58 | < 0.62 | < 0.10 | < 3.06 | < 0.58 | < 0.58 | < 0.10 | < 2.98 | < 1.55 | < 0.65 | < 0.14 | < 3.00 | < 2.69 |
| PFDoS | 79780-39-5 | < 0.52 | < 0.16 | < 2.83 | < 0.51 | < 0.55 | < 0.16 | < 3.06 | < 0.51 | < 0.51 | < 0.16 | < 2.98 | < 1.56 | < 0.57 | < 0.21 | < 3.00 | < 2.37 |
| 3:3 FTCA | 356-02-5 | < 1.65 | < 0.67 | < 11.30 | < 1.62 | < 1.74 | < 0.68 | < 12.20 | < 1.61 | < 1.61 | < 0.68 | < 11.90 | < 8.03 | < 1.82 | < 0.90 | < 12.00 | < 7.53 |
| 5:3 FTCA | 914637-49-3 | < 7.08 | 11.9 J | < 70.80 | 7.31 J | < 7.44 | < 3.69 | < 76.50 | < 6.90 | < 6.91 | < 3.67 | < 74.40 | < 40.10 | 11.9 J | 19 J | < 75.00 | < 32.30 |
| 7:3 FTCA | 812-70-4 | < 3.75 | < 5.44 | < 70.80 | < 3.69 | < 3.95 | < 5.54 | < 76.50 | < 3.66 | < 3.66 | < 5.52 | < 74.40 | < 40.10 | < 4.14 | < 7.34 | < 75.00 | < 17.10 |
| 6:2 FTCA | 53826-12-3 | | 11.9 | | | | < 4.74 | | | | < 14.6 | | | | 16.6 H | | |
| 8:2 FTCA | 27854-31-5 | | < 4.68 | | | | < 4.74 | | | | < 14.6 | | | | < 4.77 | | |
| 10:2 FTCA | 53826-13-4 | | < 4.68 | | | | < 4.74 | | | | < 14.60 | | | | < 4.77 | | |
| 6:2 FTUCA | 70887-88-6 | | < 0.93 | | | | < 0.94 | | | | < 2.89 | | | | < 0.95 | | |
| 8:2 FTUCA | 70887-84-2 | | < 0.46 | | | | < 0.47 | | | | < 1.45 | | | | < 0.47 | | |
| 4:2 FTS | 757124-72-4 | < 1.33 | < 0.95 | < 11.30 | < 1.30 | < 1.40 | < 0.96 | < 12.20 | < 1.29 | < 1.30 | < 0.96 | < 11.90 | < 6.02 | < 1.46 | < 1.27 | < 12.00 | < 6.05 |
| 6:2 FTS | 27619-97-2 | < 1.28 | < 5.25 | < 10.20 | < 1.25 | < 1.34 | < 5.35 | < 11.00 | < 1.24 | < 1.25 | < 5.32 | < 10.70 | < 6.09 | < 1.41 | < 7.07 | < 10.80 | < 5.81 |
| 8:2 FTS | 39108-34-4 | < 1.96 | < 0.92 | < 9.63 | < 1.92 | < 2.06 | < 0.94 | < 10.40 | < 1.91 | < 1.91 | < 0.93 | < 10.10 | < 6.16 | < 2.15 | < 1.24 | < 10.20 | < 8.91 |
| 10:2 FTS | 120226-60-0 | | < 0.929 | | | | < 0.94 | | | | < 2.89 | | | | < 0.95 | | |
| NMeFOSE | 24448-09-7 | < 2.72 | < 2.09 | < 28.30 | 4.88 J | < 2.86 | 4.03 J | < 30.60 | < 2.65 | < 2.65 | < 2.11 | < 29.80 | < 16.10 | < 3.00 | < 2.81 | < 30.00 | < 12.40 |
| NEtFOSE | 1691-99-2 | < 2.67 | < 2.50 | < 28.30 | < 2.62 | < 2.80 | < 2.55 | < 30.60 | < 2.60 | < 2.60 | < 2.54 | < 29.80 | < 16.10 | < 2.94 | < 3.37 | < 30.00 | < 12.20 |
| PFOSA | 754-91-6 | < 0.42 | < 0.41 | < 2.83 | < 0.41 | < 0.44 | < 0.42 | < 3.06 | < 0.41 | < 0.41 | < 0.41 | < 2.98 | < 1.61 | < 0.46 | < 0.55 | < 3.00 | < 1.90 |
| NMeFOSA | 31506-32-8 | < 1.06 | < 0.19 | < 2.83 | < 1.04 | < 1.11 | < 0.19 | < 3.06 | < 1.03 | < 1.03 | < 0.19 | < 2.98 | < 1.61 | < 1.16 | < 0.25 | < 3.00 | < 4.81 |
| NEtFOSA | 4151-50-2 | < 1.02 | < 0.54 | < 7.93 | < 1.00 | < 1.07 | < 0.55 | < 8.57 | < 0.99 | < 0.99 | < 0.55 | < 8.33 | < 1.61 | < 1.12 | < 0.73 | < 8.40 | < 4.63 |
| PFBSA | 30334-69-1 | | 0.298 | | | | < 0.09 | | | | 1.06 K | | | | 0.284 K | | |
| PFHxSA | 41997-13-1 | | < 0.09 | | | | < 0.09 | | | | < 0.29 | | | | < 0.09 | | |
| PFHpSA | 82765-77-3 | | < 0.09 | | | | < 0.09 | | | | < 0.29 | | | | < 0.09 | | |
| PFDSA | 4262-70-8 | | < 0.09 | | | | < 0.09 | | | | < 0.29 | | | | < 0.09 | | |
| NMeFBSA | 68298-12-4 | | < 0.19 | | | | < 0.19 | | | | < 0.58 | | | | < 0.20 | | |
| NMeFOSAA | 2355-31-9 | < 0.72 | 0.822 K J | < 2.83 | 0.751 J | < 0.76 | < 0.33 | < 3.06 | < 0.70 | < 0.71 | < 0.33 | < 2.98 | < 1.61 | < 0.80 | < 0.44 | < 3.00 | < 3.29 |
| NEtFOSAA | 2991-50-6 | < 0.72 | < 0.241 | < 2.83 | < 0.708 | < 0.76 | < 0.25 | < 3.06 | < 0.70 | < 0.70 | < 0.24 | < 2.98 | < 1.61 | < 0.79 | < 0.33 | < 3.00 | < 3.28 |
| NMeFBSAA | 159381-10-9 | | 1.2 | | | | 0.812 | | | | < 0.58 | | | | 1.94 | | |
| NMeFHSAA | 715646-50-7 | | < 0.19 | | | | < 0.19 | | | | < 0.58 | | | | < 0.19 | | |
| PFOSAA | 2806-24-8 | | < 0.19 | | | | < 0.19 | | | | < 0.58 | | | | < 0.19 | | |
| HFPO-DA | 13252-13-6 | < 1.78 | < 0.38 | | < 1.75 | < 1.87 | < 0.39 | | < 1.73 | < 1.74 | < 0.39 | | < 6.70 | < 1.96 | < 0.51 | | < 8.10 |
| ADONA | 919005-14-4 | < 1.66 | < 1.21 | | < 1.63 | < 1.75 | < 1.24 | | < 1.62 | < 1.62 | < 1.23 | | < 6.34 | < 1.83 | < 1.64 | | < 7.58 |
| PFMBA | 863090-89-5 | < 0.47 | < 0.11 | | < 0.46 | < 0.50 | < 0.11 | | < 0.46 | < 0.46 | < 0.11 | | < 3.21 | < 0.52 | < 0.15 | | < 2.15 |
| NFDHA | 151772-58-6 | < 1.62 | < 6.38 | | < 1.59 | < 1.70 | < 6.50 | | < 1.58 | < 1.58 | < 6.46 | | < 3.21 | < 1.79 | < 8.60 | | < 7.39 |
| PFMPA | 377-73-1 | < 0.71 | < 0.17 | | < 0.70 | < 0.75 | < 0.17 | | 0.841 J | < 0.70 | < 0.17 | | < 3.21 | < 0.79 | < 0.23 | | < 3.26 |
| PFMOAA | 674-13-5 | | < 1.88 | | | | < 1.90 | | | | < 5.84 | | | | < 2.28 | | |
| PFO3OA | 39492-89-2 | | < 1.88 | | | | < 1.90 | | | | < 5.84 | | | | < 1.91 | | |
| PFO4DA | 39492-90-5 | | < 1.88 | | | | < 1.90 | | | | < 5.84 | | | | < 1.91 | | |
| PFO5DoDA | 39492-91-6 | | < 1.86 | | | | < 1.88 | | | | < 5.78 | | | | < 2.08 | | |
| PFO6TeA | N/A | | < 2.39 | | | | < 1.90 | | | | < 5.84 | | | | < 1.91 | | |
| 9Cl-PF3ONS | 756426-58-1 | < 2.01 | < 0.86 | | < 1.97 | < 2.11 | < 0.88 | | < 1.96 | < 1.96 | < 0.87 | | < 6.26 | < 2.21 | < 1.16 | | < 9.15 |
| 11Cl-PF3OUdS | 763051-92-9 | < 2.02 | < 0.96 | | < 1.98 | < 2.12 | < 0.97 | | < 1.97 | < 1.97 | < 0.97 | | < 6.02 | < 2.22 | < 1.29 | | < 9.20 |
| PFEESA | 113507-82-7 | < 0.39 | < 0.13 | | < 0.38 | < 0.41 | < 0.13 | | < 0.38 | < 0.38 | < 0.13 | | < 2.86 | < 0.43 | < 0.18 | | < 1.77 |

# Table S3-E: PFAS Data - Site B (cont'd)

| Abbreviation | CAS Number | Oct-24 | Nov-24 | | Dec-24 | Jan-25 | Feb-25 | | Mar-25 | Apr-25 | May-25 | | Jun-25 | Jul-25 | Aug-25 | | Sep-25 |
|---|---|---|---|---|---|---|---|---|---|---|---|---|---|---|---|---|---|
| | | Effluent | Effluent | Effluent (post-TOP) | Effluent | Effluent | Effluent | Effluent (post-TOP) | Effluent | Effluent | Effluent | Effluent (post-TOP) | Effluent | Effluent | Effluent | Effluent (post-TOP) | Effluent |
| Nafion BP 1 | 29311-67-9 | | < 0.186 | | | | < 0.19 | | | | < 0.58 | | | | < 0.19 | | |
| Nafion BP2 | 749836-20-2 | | < 0.186 | | | | < 0.19 | | | | < 0.58 | | | | < 0.19 | | |
| PFecHS | 133201-07-7 | | < 0.241 | | | | < 0.25 | | | | < 0.75 | | | | < 0.25 | | |
| PFHxPA | 40143-76-8 | | < 2.32 | | | | < 2.35 | | | | < 7.23 | | | | < 2.37 | | |
| PFOPA | 40143-78-0 | | < 2.32 | | | | < 2.35 | | | | < 7.23 | | | | 6.85 | | |
| PFDPA | 52299-26-0 | | < 2.32 | | | | < 2.35 | | | | < 7.23 | | | | 8.85 | | |
| 6:2 PAP | 57678-01-0 | | 4.56 | | | | < 2.35 | | | | < 7.23 | | | | 3.75 | | |
| 8:2 PAP | 57678-03-2 | | < 2.36 | | | | < 2.39 | | | | < 7.35 | | | | 7.86 K | | |
| 6:6 PFPi | 40143-77-9 | | < 0.09 | | | | < 0.09 | | | | < 0.29 | | | | < 0.09 | | |
| 6:8 PFPi | 610800-34-5 | | < 0.09 | | | | < 0.09 | | | | < 0.29 | | | | < 0.09 | | |
| 8:8 PFPi | 40143-79-1 | | < 0.09 | | | | < 0.09 | | | | < 0.29 | | | | < 0.09 | | |
| 6:2 diPAP | 57677-95-9 | | 69.9 | | | | 23 | | | | 2.92 | | | | 57 | | |
| 8:2 diPAP | 678-41-1 | | 17 | | | | 13.6 K | | | | 3.61 | | | | 44.4 K | | |
| 4:2 FTOH | 2043-47-2 | | < 69.5 | | | | < 31.2 | | | | < 148 | | | | < 170 | | |
| 6:2 FTOH | 647-42-7 | | 64.1 | | | | 23.9 | | | | 87.4 | | | | 44.9 | | |
| 7:2 sFTOH | 24015-83-6 | | < 13.3 | | | | < 4.56 | | | | < 8.72 | | | | < 7.19 | | |
| 8:2 FTOH | 678-39-7 | | < 3.08 | | | | < 4.21 | | | | < 5.30 | | | | < 2.02 | | |
| 10:2 FTOH | 865-86-1 | | 1.57 | | | | < 1.01 | | | | < 1.01 | | | | 2.05 | | |
| 12:2 FTOH | 39239-77-5 | | < 0.898 | | | | < 1.01 | | | | < 1.01 | | | | < 1.01 | | |
| AOF (µg/L) | N/A | | 4.39 | | | | 4.34 | | | | 3.47 | | | | 5.0 A | | |

All values are in units of ng/L. CAS = Chemical Abstracts Service registry number. J = The amount detected is below the Reporting Limit/Limit of Quantitation. K = Peak detected but did not meet quantification criteria. Result reported represents the estimated maximum possible concentration. I = Ion transition ratio is outside of the acceptance criteria. A = Sample analyzed out of hold time. H = Concentration is estimated.

# Table S3-F: PFAS Data - Site C

| Abbreviation | CAS Number | Oct-24 | Nov-24 | | Dec-24 | Jan-25 | Feb-25 | | Mar-25 | | Apr-25 | May-25 | | Jun-25 | | Jul-25 | Aug-25 | | Sep-25 |
|---|---|---|---|---|---|---|---|---|---|---|---|---|---|---|---|---|---|---|---|
| | | Effluent | Effluent | Effluent (post-TOP) | Effluent | Effluent | Effluent | Effluent (post-TOP) | Influent | Effluent | Effluent | Effluent | Effluent (post-TOP) | Influent | Effluent | Effluent | Effluent | Effluent (post-TOP) | Effluent |
| PFPrA | 422-64-0 | | < 17.6 | | | | < 19.3 | | | | | < 20.0 | | | | | < 19.0 | | |
| PFBA | 375-22-4 | < 1.67 | 2.42 J | 16 J | 2.23 | < 1.62 | 1.67 J | 16.1 J | 1.75 J | 1.67 J | 2.04 J | 1.56 J | 17.2 J | 4.96 J | 1.35 J | 2.96 J | 2.39 J | 24.3 | < 1.63 |
| PFPeA | 2706-90-3 | 6.6 | 6.57 | 22.5 J | 5.43 | 5.58 | 2.86 J | 31.5 | 2.35 J | 2.87 J | 4.25 | 2.81 J | 22 J | 2.55 J | 2.44 J | 3 I J | 3.14 | 42.3 | 3.45 |
| PFHxA | 307-24-4 | 7.33 | 5.13 | 12.7 | 4.9 | 5.43 | 3.32 | 22.2 | 2.47 | 2.97 | 4.15 | 3.85 | 14.1 | 2.89 | 3.43 | 3.94 | 4.21 | 21.2 | 4.04 |
| PFHpA | 375-85-9 | 2.11 | 1.5 | 7.56 J | 2 | 2.07 | 1.1 J | 16.4 | 1.45 J | 1.42 J | 2.08 | 1.23 J | 12.7 | 1.44 J | 1.35 J | 1.69 J | 1.44 J | 15.1 | 1.76 |
| PFOA | 335-67-1 | 7.69 | 6.04 | 11.9 | 8.07 | 8.43 | 5.36 | 17.9 | 7.16 | 6.5 | 6.81 | 5.4 | 14 | 5.35 | 5.17 | 7.33 | 4.86 | 13.4 | 5.99 |
| PFNA | 375-95-1 | < 0.25 | < 0.20 | < 2.96 | 0.486 J | 0.705 I J | 0.392 J | 6.9 J | 0.386 J | 0.311 J | 0.414 J | 0.502 J | 4.21 J | 0.431 J | 0.292 J | 0.394 I J | 0.368 K J | 5.99 J | 0.372 J |
| PFDA | 335-76-2 | < 0.44 | < 0.22 | < 2.96 | < 0.43 | < 0.43 | 0.433 J | 5.22 J | < 0.44 | < 0.44 | < 0.44 | 0.605 K J | < 3.06 | 0.415 K J | 0.583 J | < 0.46 | 0.318 J | < 3.02 | < 0.43 |
| PFUnA | 2058-94-8 | < 0.43 | < 0.14 | < 2.96 | < 0.42 | < 0.42 | < 0.14 | < 3.08 | < 0.43 | < 0.43 | < 0.42 | < 0.14 | 10.1 K J | < 0.14 | < 0.14 | < 0.45 | < 0.14 | < 3.02 | < 0.42 |
| PFDoDA | 307-55-1 | < 0.23 | < 0.26 | < 2.36 | < 0.23 | < 0.23 | < 0.26 | < 2.47 | < 0.23 | < 0.23 | < 0.23 | < 0.26 | < 2.44 | < 0.27 | < 0.26 | < 0.24 | < 0.27 | < 2.42 | < 0.23 |
| PFTrDA | 72629-94-8 | < 0.27 | < 0.14 | < 2.96 | < 0.26 | < 0.26 | < 0.14 | < 3.08 | < 0.26 | < 0.27 | < 0.26 | < 0.14 | < 3.06 | < 0.14 | < 0.14 | < 0.28 | < 0.14 | < 3.02 | < 0.26 |
| PFTeDA | 376-06-7 | < 0.25 | < 0.20 | < 2.96 | < 0.24 | < 0.24 | < 0.21 | < 3.08 | < 0.25 | < 0.25 | < 0.25 | < 0.21 | < 3.06 | < 0.21 | < 0.21 | < 0.26 | < 0.21 | < 3.02 | < 0.24 |
| PFHxDA | 67905-19-5 | | < 2.34 | | | | < 2.37 | | | | | < 7.26 | | | | | < 2.35 | | |
| PFODA | 16517-11-6 | | < 2.38 | | | | < 2.40 | | | | | < 7.37 | | | | | < 2.39 | | |
| PFMeS | 37181-39-8 | | < 8.78 | | | | < 9.66 | | | | | < 10.00 | | | | | < 9.49 | | |
| PFEtS | 354-88-1 | | < 5.75 | | | | < 4.85 | | | | | < 5.02 | | | | | < 4.89 | | |
| PFPrS | 423-41-6 | | < 4.47 | | | | < 4.85 | | | | | < 5.02 | | | | | < 4.83 | | |
| PFBS | 375-73-5 | 3.64 | 4.5 | < 2.96 | 3.49 | 2.32 | 2.53 | < 3.08 | 3.42 | 2.69 | 3.36 | 2.71 | < 3.06 | 2.92 | 1.88 | 3.01 | 1.96 | < 3.02 | 2.31 |
| PFPeS | 2706-91-4 | < 0.50 | < 0.14 | < 2.96 | 0.502 J | < 0.49 | 0.337 J | < 3.08 | < 0.50 | < 0.50 | < 0.495 | 0.331 J | < 3.06 | 0.321 J | < 0.14 | < 0.53 | 0.325 J | < 3.02 | < 0.492 |
| PFHxS | 355-46-4 | 2.31 | 1.81 | < 3.40 | 2.23 | 2.05 | 1.24 J | < 3.54 | 1.59 | 1.77 | 1.46 J | 1.39 J | < 3.51 | 1.54 J | 1.55 J | 2.05 I | 1.56 J | < 3.48 | 1.55 |
| PFHpS | 375-92-8 | < 0.39 | < 0.13 | < 2.96 | < 0.38 | < 0.38 | 0.134 J | < 3.08 | < 0.39 | < 0.39 | < 0.386 | < 0.13 | < 3.06 | < 0.13 | 0.19 J | < 0.41 | 0.174 J | < 3.02 | < 0.383 |
| PFOS | 1763-23-1 | 4.48 | 5.77 | 5.64 J | 6.17 | 4.87 | 4.29 | 4.89 J | 5.85 | 4.5 | 8.23 I | 4.86 | 4.27 J | 3.04 | 4.59 | 5.49 | 5.11 | 5.04 J | 5.18 |
| PFNS | 68259-12-1 | < 0.63 | < 0.12 | < 2.96 | < 0.61 | < 0.61 | < 0.12 | < 3.08 | < 0.62 | < 0.63 | < 0.62 | < 0.12 | < 3.06 | < 0.12 | < 0.12 | < 0.66 | < 0.12 | < 3.02 | < 0.61 |
| PFDS | 335-77-3 | < 0.59 | < 0.10 | < 2.96 | < 0.57 | < 0.57 | < 0.10 | < 3.08 | < 0.58 | < 0.58 | < 0.58 | < 0.10 | < 3.06 | < 0.11 | < 0.10 | < 0.61 | < 0.11 | < 3.02 | < 0.57 |
| PFDoS | 79780-39-5 | < 0.52 | < 0.16 | < 2.96 | < 0.50 | < 0.50 | < 0.16 | < 3.08 | < 0.51 | < 0.51 | < 0.51 | < 0.16 | < 3.06 | < 0.16 | < 0.16 | < 0.54 | < 0.16 | < 3.02 | < 0.51 |
| 3:3 FTCA | 356-02-5 | < 1.65 | < 0.66 | < 11.80 | < 1.60 | < 1.60 | < 0.67 | < 12.30 | < 1.63 | < 1.63 | < 1.62 | < 0.67 | < 12.20 | < 0.69 | < 0.68 | < 1.72 | < 0.68 | < 12.10 | < 1.61 |
| 5:3 FTCA | 914637-49-3 | < 7.05 | < 3.57 | < 73.90 | < 6.86 | < 6.84 | < 3.66 | < 77.00 | < 6.99 | < 7.00 | < 6.93 | < 3.65 | < 76.40 | < 3.72 | 3.76 J | < 7.36 | < 3.71 | < 75.60 | < 6.89 |
| 7:3 FTCA | 812-70-4 | < 3.74 | < 5.36 | < 73.90 | < 3.64 | < 3.63 | < 5.50 | < 77.00 | < 3.71 | < 3.71 | < 3.67 | < 5.48 | < 76.40 | < 5.59 | < 5.54 | < 3.91 | < 5.58 | < 75.60 | < 3.65 |
| 6:2 FTCA | 53826-12-3 | | < 4.73 | | | | < 4.77 | | | | | < 14.6 | | | | | < 4.74 | | |
| 8:2 FTCA | 27854-31-5 | | < 4.73 | | | | < 4.77 | | | | | < 14.6 | | | | | < 4.74 | | |
| 10:2 FTCA | 53826-13-4 | | < 4.73 | | | | < 4.77 | | | | | < 14.60 | | | | | < 4.74 | | |
| 6:2 FTUCA | 70887-88-6 | | < 0.94 | | | | < 0.95 | | | | | < 2.90 | | | | | < 0.94 | | |
| 8:2 FTUCA | 70887-84-2 | | < 0.47 | | | | < 0.47 | | | | | < 1.45 | | | | | < 0.47 | | |
| 4:2 FTS | 757124-72-4 | < 1.32 | < 0.93 | < 11.80 | < 1.29 | < 1.28 | < 0.96 | < 12.30 | < 1.31 | < 1.31 | < 1.30 | < 0.95 | < 12.20 | < 0.97 | < 0.96 | < 1.38 | < 0.97 | < 12.10 | < 1.29 |
| 6:2 FTS | 27619-97-2 | < 1.27 | < 5.17 | < 10.60 | < 1.24 | < 1.23 | < 5.30 | < 11.10 | < 1.26 | < 1.26 | < 1.25 | < 5.28 | < 11.00 | < 5.39 | < 5.35 | < 1.33 | < 5.38 | < 10.90 | < 1.24 |
| 8:2 FTS | 39108-34-4 | < 1.95 | < 0.90 | < 10.00 | < 1.89 | 3.59 J | 4.44 J | < 10.50 | < 1.93 | < 1.93 | < 1.91 | 2.1 J | < 10.40 | 2.12 J | 2.87 J | < 2.03 | < 0.94 | < 10.30 | < 1.90 |
| 10:2 FTS | 120226-60-0 | | < 0.94 | | | | 1.92 | | | | | < 2.90 | | | | | 1.43 | | |
| NMeFOSE | 24448-09-7 | < 2.71 | < 2.06 | < 29.60 | < 2.63 | < 2.63 | < 2.11 | < 30.80 | < 2.68 | < 2.69 | < 2.66 | < 2.10 | < 30.60 | < 2.14 | < 2.13 | < 2.83 | < 2.14 | < 30.20 | < 2.64 |
| NEtFOSE | 1691-99-2 | < 2.66 | < 2.47 | < 29.60 | < 2.58 | < 2.58 | < 2.53 | < 30.80 | < 2.63 | < 2.64 | < 2.61 | < 2.52 | < 30.60 | < 2.57 | < 2.55 | < 2.77 | < 2.57 | < 30.20 | < 2.59 |
| PFOSA | 754-91-6 | < 0.42 | < 0.40 | < 2.96 | 0.708 J | 0.547 J | < 0.41 | < 3.08 | < 0.41 | 0.421 I J | < 0.41 | < 0.41 | < 3.06 | < 0.42 | < 0.42 | 0.435 I J | < 0.42 | < 3.02 | < 0.41 |
| NMeFOSA | 31506-32-8 | < 1.05 | < 0.18 | < 2.96 | < 1.02 | < 1.02 | < 0.19 | < 3.08 | < 1.04 | < 1.04 | < 1.03 | < 0.19 | < 3.06 | < 0.19 | < 0.19 | < 1.10 | < 0.19 | < 3.02 | < 1.03 |
| NEtFOSA | 4151-50-2 | < 1.01 | < 0.53 | < 8.27 | < 0.99 | < 0.98 | < 0.54 | < 8.63 | < 1.00 | < 1.01 | < 1.00 | < 0.54 | < 8.55 | < 0.55 | < 0.55 | < 1.06 | < 0.55 | < 8.47 | < 0.99 |
| PFBSA | 30334-69-1 | | 0.259 | | | | 0.124 K | | | | | < 0.29 | | | | | 0.233 | | |
| PFHxSA | 41997-13-1 | | < 0.09 | | | | < 0.09 | | | | | < 0.29 | | | | | < 0.09 | | |
| PFHpSA | 82765-77-3 | | < 0.09 | | | | < 0.09 | | | | | < 0.29 | | | | | < 0.09 | | |
| PFDSA | 4262-70-8 | | < 0.09 | | | | < 0.09 | | | | | < 0.29 | | | | | < 0.09 | | |
| NMeFBSA | 68298-12-4 | | < 0.19 | | | | < 0.19 | | | | | < 0.58 | | | | | < 0.19 | | |
| NMeFOSAA | 2355-31-9 | < 0.72 | < 0.32 | < 2.96 | < 0.70 | < 0.70 | < 0.33 | < 3.08 | < 0.71 | < 0.72 | < 0.71 | 0.577 K J | < 3.06 | < 0.33 | < 0.33 | < 0.75 | < 0.33 | < 3.02 | < 0.703 |
| NEtFOSAA | 2991-50-6 | < 0.72 | < 0.24 | < 2.96 | < 0.70 | < 0.70 | < 0.24 | < 3.08 | < 0.71 | < 0.71 | < 0.71 | < 0.24 | < 3.06 | < 0.25 | < 0.25 | < 0.75 | < 0.25 | < 3.02 | < 0.701 |
| NMeFBSAA | 159381-10-9 | | 0.223 | | | | < 0.19 | | | | | < 0.58 | | | | | 0.29 | | |
| NMeFHSAA | 715646-50-7 | | < 0.19 | | | | < 0.19 | | | | | < 0.58 | | | | | < 0.19 | | |
| PFOSAA | 2806-24-8 | | < 0.19 | | | | < 0.19 | | | | | < 0.58 | | | | | < 0.19 | | |
| HFPO-DA | 13252-13-6 | < 1.77 | < 0.38 | | < 1.72 | < 1.72 | < 0.38 | | < 1.76 | < 1.76 | < 1.74 | < 0.38 | | < 0.39 | < 0.39 | < 1.85 | < 0.39 | | < 1.73 |
| ADONA | 919005-14-4 | < 1.66 | < 1.20 | | < 1.61 | < 1.61 | < 1.23 | | < 1.64 | < 1.64 | < 1.63 | < 1.22 | | < 1.25 | < 1.24 | < 1.73 | < 1.24 | | < 1.62 |
| PFMBA | 863090-89-5 | < 0.47 | < 0.11 | | < 0.46 | < 0.46 | < 0.11 | | < 0.47 | < 0.47 | < 0.46 | < 0.11 | | < 0.11 | < 0.11 | < 0.49 | < 0.11 | | < 0.46 |
| NFDHA | 151772-58-6 | < 1.61 | < 6.29 | | < 1.57 | < 1.57 | < 6.44 | | < 1.60 | < 1.60 | < 1.59 | < 6.42 | | < 6.55 | < 6.50 | < 1.69 | < 6.54 | | < 1.58 |
| PFMPA | 377-73-1 | < 0.71 | < 0.16 | | < 0.69 | < 0.69 | < 0.17 | | < 0.71 | < 0.71 | < 0.70 | < 0.17 | | < 0.17 | < 0.17 | < 0.74 | < 0.17 | | < 0.70 |

# Table S3-F: PFAS Data - Site C (cont'd)

| Abbreviation | CAS Number | Oct-24 | Nov-24 | | Dec-24 | Jan-25 | Feb-25 | | Mar-25 | | Apr-25 | May-25 | | Jun-25 | | Jul-25 | Aug-25 | | Sep-25 |
|---|---|---|---|---|---|---|---|---|---|---|---|---|---|---|---|---|---|---|---|
| | | Effluent | Effluent | Effluent (post-TOP) | Effluent | Effluent | Effluent | Effluent (post-TOP) | Influent | Effluent | Effluent | Effluent | Effluent (post-TOP) | Influent | Effluent | Effluent | Effluent | Effluent (post-TOP) | Effluent |
| PFMOAA | 674-13-5 | | < 1.89 | | | | < 1.91 | | | | | < 5.86 | | | | | < 1.90 | | |
| PFO3OA | 39492-89-2 | | < 1.89 | | | | < 1.91 | | | | | < 5.86 | | | | | < 1.90 | | |
| PFO4DA | 39492-90-5 | | < 1.89 | | | | < 1.91 | | | | | < 5.86 | | | | | < 1.90 | | |
| PFO5DoDA | 39492-91-6 | | < 1.88 | | | | < 1.89 | | | | | < 5.81 | | | | | < 1.88 | | |
| PFO6TeA | N/A | | < 1.89 | | | | < 1.91 | | | | | < 5.86 | | | | | < 1.90 | | |
| 9Cl-PF3ONS | 756426-58-1 | < 2.00 | < 0.85 | | < 1.95 | < 1.94 | < 0.23 | | < 1.98 | < 1.99 | < 1.96 | < 0.87 | | < 0.89 | < 0.88 | < 2.09 | < 0.88 | | < 1.95 |
| 11Cl-PF3OUdS | 763051-92-9 | < 2.01 | < 0.94 | | < 1.96 | < 1.95 | < 0.87 | | < 1.99 | < 2.00 | < 1.97 | < 0.96 | | < 0.98 | < 0.97 | < 2.10 | < 0.98 | | < 1.96 |
| PFEESA | 113507-82-7 | < 0.39 | < 0.13 | | < 0.38 | < 0.38 | < 0.96 | | < 0.38 | < 0.39 | < 0.38 | < 0.13 | | < 0.13 | < 0.13 | < 0.41 | < 0.13 | | < 0.38 |
| Nafion BP 1 | 29311-67-9 | | < 0.19 | | | | < 0.13 | | | | | < 0.58 | | | | | < 0.19 | | |
| Nafion BP2 | 749836-20-2 | | < 0.19 | | | | < 0.19 | | | | | < 0.58 | | | | | < 0.19 | | |
| PFecHS | 133201-07-7 | | < 0.24 | | | | < 0.25 | | | | | < 0.76 | | | | | < 0.24 | | |
| PFHxPA | 40143-76-8 | | 2.61 | | | | < 2.37 | | | | | < 7.26 | | | | | 4.34 | | |
| PFOPA | 40143-78-0 | | < 2.34 | | | | < 2.37 | | | | | < 7.26 | | | | | 5.06 | | |
| PFDPA | 52299-26-0 | | < 2.34 | | | | < 2.37 | | | | | < 7.26 | | | | | < 2.35 | | |
| 6:2 PAP | 57678-01-0 | | < 2.34 | | | | < 2.37 | | | | | < 7.26 | | | | | < 2.35 | | |
| 8:2 PAP | 57678-03-2 | | < 2.38 | | | | < 2.40 | | | | | < 7.37 | | | | | 2.71 K | | |
| 6:6 PFPi | 40143-77-9 | | < 0.09 | | | | < 0.09 | | | | | < 0.29 | | | | | < 0.09 | | |
| 6:8 PFPi | 610800-34-5 | | < 0.09 | | | | < 0.09 | | | | | < 0.29 | | | | | < 0.09 | | |
| 8:8 PFPi | 40143-79-1 | | < 0.09 | | | | < 0.09 | | | | | < 0.29 | | | | | < 0.09 | | |
| 6:2 diPAP | 57677-95-9 | | 0.504 | | | | 0.815 | | | | | < 1.45 | | | | | < 0.47 | | |
| 8:2 diPAP | 678-41-1 | | < 0.75 | | | | < 0.51 | | | | | < 1.45 | | | | | 0.575 K | | |
| 4:2 FTOH | 2043-47-2 | | < 46.40 | | | | 48.3 | | | | | < 226.00 | | | | | < 61.60 | | |
| 6:2 FTOH | 647-42-7 | | < 4.07 | | | | 6.44 | | | | | < 9.21 | | | | | < 4.85 | | |
| 7:2 sFTOH | 24015-83-6 | | < 11.20 | | | | < 4.42 | | | | | < 33.10 | | | | | < 12.70 | | |
| 8:2 FTOH | 678-39-7 | | < 1.86 | | | | < 2.65 | | | | | < 2.01 | | | | | < 2.01 | | |
| 10:2 FTOH | 865-86-1 | | < 0.93 | | | | < 1.00 | | | | | < 1.01 | | | | | < 1.00 | | |
| 12:2 FTOH | 39239-77-5 | | < 0.93 | | | | < 1.00 | | | | | < 1.01 | | | | | < 1.00 | | |
| AOF (µg/L) | N/A | | < 1.49 | | | | < 1.48 | | | | | < 1.46 | | | | | 2.0 A | | |

All values are in units of ng/L. CAS = Chemical Abstracts Service registry number. J = The amount detected is below the Reporting Limit/Limit of Quantitation. K = Peak detected but did not meet quantification criteria. Result reported represents the estimated maximum possible concentration. I = Ion transition ratio is outside of the acceptance criteria. A = Sample analyzed out of hold time.

# Table S3-G: PFAS Data - Site D

| Abbreviation | CAS Number | Oct-24 | Nov-24 | | Dec-24 | Jan-25 | Feb-25 | | Mar-25 | Apr-25 | May-25 | | Jun-25 | Jul-25 | Aug-25 | | Sep-25 |
|---|---|---|---|---|---|---|---|---|---|---|---|---|---|---|---|---|---|
| | | Effluent | Effluent | Effluent (post-TOP) | Effluent | Effluent | Effluent | Effluent (post-TOP) | Effluent | Effluent | Effluent | Effluent (post-TOP) | Effluent | Effluent | Effluent | Effluent (post-TOP) | Effluent |
| PFPrA | 422-64-0 | | < 18.8 | | | | < 19.9 | | | | < 19.9 | | | | < 20.0 | | |
| PFBA | 375-22-4 | < 1.67 | < 0.40 | < 11.9 | < 1.68 | < 1.72 | 1.25 J | < 11.9 | < 1.63 | < 1.64 | 2.39 J | < 11.8 | < 16.0 | 2.75 J | < 0.75 | < 12.8 | < 1.66 |
| PFPeA | 2706-90-3 | 4.94 | 2.64 J | 11.7 J | 1.83 I J | < 0.45 | 1.19 J | 17.3 J | 1.37 I J | 1.87 I J | < 1.17 | 11.2 J | < 4.19 | 1.84 I J | 2.43 J | 16.2 J | < 0.436 |
| PFHxA | 307-24-4 | 4.62 | 1.87 | 8.23 J | 3.12 | 0.912 J | 0.883 J | 11.2 J | 1.21 J | 2.03 | 1.06 J | 11.7 J | < 2.73 | 1.55 J | 1.59 J | 13.1 | 2.28 |
| PFHpA | 375-85-9 | < 0.28 | 0.386 J | 5.91 J | 0.547 J | < 0.29 | 0.278 J | 12.8 | 0.346 J | 0.539 J | 0.333 J | 11.7 J | < 2.71 | 0.471 J | < 0.47 | 13.2 | 0.471 J |
| PFOA | 335-67-1 | < 1.85 | 0.673 J | 5.99 J | 1.94 J | < 1.91 | 0.585 J | 12.9 | < 1.82 | < 1.83 | 0.762 J | 13.1 | < 17.8 | < 1.98 | 0.923 J | 12 J | < 1.85 |
| PFNA | 375-95-1 | < 0.25 | < 0.20 | < 2.98 | 0.508 J | < 0.26 | < 0.21 | 5.58 J | < 0.25 | 0.271 I J | < 0.21 | 4.71 J | < 2.42 | 0.289 J | < 0.38 | 6.16 J | < 0.25 |
| PFDA | 335-76-2 | < 0.44 | < 0.22 | < 2.98 | 0.481 J | < 0.46 | < 0.23 | 3.93 J | < 0.43 | < 0.44 | < 0.23 | < 2.96 | < 4.25 | < 0.47 | < 0.42 | 4.41 J | < 0.44 |
| PFUnA | 2058-94-8 | < 0.43 | < 0.14 | < 2.98 | < 0.44 | < 0.44 | < 0.14 | < 2.98 | < 0.42 | < 0.42 | 0.165 K J | 7.32 K J | < 4.13 | < 0.46 | 0.338 K J | < 3.21 | < 0.43 |
| PFDoDA | 307-55-1 | < 0.23 | < 0.26 | < 2.38 | < 0.24 | < 0.24 | < 0.26 | < 2.39 | < 0.23 | < 0.23 | < 0.26 | < 2.37 | < 2.23 | < 0.25 | < 0.49 | < 2.57 | < 0.23 |
| PFTrDA | 72629-94-8 | < 0.27 | < 0.14 | < 2.98 | < 0.27 | < 0.28 | < 0.14 | < 2.98 | < 0.26 | < 0.26 | < 0.14 | < 2.96 | < 2.56 | < 0.29 | < 0.26 | < 3.21 | < 0.27 |
| PFTeDA | 376-06-7 | < 0.25 | < 0.20 | < 2.98 | < 0.25 | < 0.26 | < 0.21 | < 2.98 | < 0.24 | < 0.25 | < 0.21 | < 2.96 | < 2.39 | < 0.27 | < 0.38 | < 3.79 | < 0.25 |
| PFHxDA | 67905-19-5 | | < 2.35 | | | | < 2.35 | | | | < 7.38 | | | | < 2.39 | | |
| PFODA | 16517-11-6 | | < 2.39 | | | | < 2.39 | | | | < 7.50 | | | | < 2.43 | | |
| PFMeS | 37181-39-8 | | < 9.41 | | | | < 9.96 | | | | < 9.96 | | | | < 9.99 | | |
| PFEtS | 354-88-1 | | < 4.84 | | | | < 7.05 | | | | < 5.01 | | | | < 5.14 | | |
| PFPrS | 423-41-6 | | < 4.79 | | | | < 5.00 | | | | < 5.00 | | | | < 5.09 | | |
| PFBS | 375-73-5 | < 0.70 | < 0.16 | < 2.98 | < 0.71 | < 0.73 | 0.387 K J | < 2.98 | < 0.69 | < 0.69 | 1.15 K J | < 2.96 | < 6.75 | < 0.75 | < 0.29 | < 3.21 | < 0.70 |
| PFPeS | 2706-91-4 | < 0.50 | < 0.14 | < 2.98 | < 0.51 | < 0.52 | < 0.14 | < 2.98 | < 0.49 | < 0.50 | < 0.14 | < 2.96 | < 4.84 | < 0.54 | < 0.26 | < 3.21 | < 0.50 |
| PFHxS | 355-46-4 | < 0.53 | < 0.13 | < 3.42 | < 0.53 | < 0.54 | < 0.13 | < 3.43 | < 0.51 | < 0.52 | 0.171 J | < 3.40 | < 5.04 | < 0.56 | < 0.24 | < 3.69 | < 0.52 |
| PFHpS | 375-92-8 | < 0.39 | < 0.13 | < 2.98 | < 0.40 | < 0.41 | < 0.13 | < 2.98 | < 0.39 | < 0.39 | < 0.13 | < 2.96 | < 3.77 | < 0.42 | < 0.24 | < 3.21 | < 0.39 |
| PFOS | 1763-23-1 | < 1.22 | 0.947 K J | < 2.98 | < 1.23 | < 1.26 | 0.433 J | < 2.98 | < 1.19 | < 1.20 | 0.48 J | < 2.96 | < 11.7 | 1.5 I J | < 0.57 | < 3.21 | < 1.22 |
| PFNS | 68259-12-1 | < 0.63 | < 0.12 | < 2.98 | < 0.64 | < 0.65 | < 0.12 | < 2.98 | < 0.62 | < 0.62 | < 0.12 | < 2.96 | < 6.04 | < 0.67 | < 0.23 | < 3.21 | < 0.63 |
| PFDS | 335-77-3 | < 0.59 | < 0.10 | < 2.98 | 2.14 | < 0.61 | < 0.10 | < 2.98 | < 0.58 | < 0.58 | < 0.10 | < 2.96 | < 5.64 | < 0.63 | < 0.19 | < 3.21 | < 0.59 |
| PFDoS | 79780-39-5 | < 0.52 | < 0.16 | < 2.98 | < 0.52 | < 0.53 | < 0.16 | < 2.98 | < 0.51 | < 0.51 | < 0.16 | < 2.96 | < 4.97 | < 0.55 | < 0.29 | < 3.21 | < 0.52 |
| 3:3 FTCA | 356-02-5 | < 1.65 | < 0.67 | < 11.90 | < 1.66 | < 1.70 | < 0.68 | < 11.90 | < 1.61 | < 1.62 | < 0.68 | < 11.80 | < 15.8 | < 1.76 | < 1.25 | < 12.80 | < 1.64 |
| 5:3 FTCA | 914637-49-3 | < 7.05 | < 3.63 | < 74.40 | < 7.13 | < 7.27 | < 3.66 | < 74.60 | < 6.91 | < 6.96 | < 3.67 | < 74.00 | < 67.7 | < 7.54 | < 6.77 | < 80.30 | 7.29 J |
| 7:3 FTCA | 812-70-4 | < 3.74 | < 5.45 | < 74.40 | < 3.78 | < 3.86 | < 5.50 | < 74.60 | < 3.66 | < 3.69 | < 5.52 | < 74.00 | < 35.9 | < 4.00 | < 10.20 | < 80.30 | < 3.73 |
| 6:2 FTCA | 53826-12-3 | | 6.32 | | | | < 4.74 | | | | < 14.9 | | | | 5.97 H | | |
| 8:2 FTCA | 27854-31-5 | | < 4.74 | | | | < 4.74 | | | | < 14.9 | | | | < 4.81 | | |
| 10:2 FTCA | 53826-13-4 | | < 4.74 | | | | < 4.74 | | | | < 14.90 | | | | < 4.81 | | |
| 6:2 FTUCA | 70887-88-6 | | < 0.94 | | | | < 0.94 | | | | < 2.95 | | | | < 0.96 | | |
| 8:2 FTUCA | 70887-84-2 | | < 0.47 | | | | < 0.47 | | | | < 1.48 | | | | < 0.48 | | |
| 4:2 FTS | 757124-72-4 | < 1.32 | < 0.95 | < 11.90 | < 1.34 | < 1.36 | < 0.96 | < 11.90 | < 1.30 | < 1.31 | < 0.96 | < 11.80 | < 12.7 | < 1.41 | < 1.77 | < 12.80 | < 1.32 |
| 6:2 FTS | 27619-97-2 | < 1.27 | < 5.25 | < 10.70 | < 1.28 | < 1.31 | < 5.30 | < 10.70 | < 1.24 | < 1.25 | < 5.32 | < 10.70 | < 12.2 | < 1.36 | < 9.80 | < 11.60 | < 1.27 |
| 8:2 FTS | 39108-34-4 | < 1.95 | < 0.92 | < 10.10 | < 1.97 | < 2.01 | < 0.93 | < 10.10 | < 1.91 | < 1.92 | < 0.93 | < 10.10 | < 18.7 | < 2.08 | < 1.71 | < 10.90 | < 1.95 |
| 10:2 FTS | 120226-60-0 | | < 0.97 | | | | < 0.94 | | | | < 2.95 | | | | < 0.96 | | |
| NMeFOSE | 24448-09-7 | < 2.71 | < 2.09 | < 29.80 | < 2.74 | 3.16 J | < 2.11 | < 29.80 | < 2.65 | 3.12 J | < 2.11 | < 29.60 | < 26.0 | < 2.90 | < 3.90 | < 32.10 | 5.22 J |
| NEtFOSE | 1691-99-2 | < 2.66 | < 2.50 | < 29.80 | < 2.68 | < 2.74 | < 2.53 | < 29.80 | < 2.60 | < 2.62 | < 2.54 | < 29.60 | < 25.5 | < 2.84 | < 4.68 | < 32.10 | < 2.65 |
| PFOSA | 754-91-6 | < 0.42 | < 0.41 | < 2.98 | < 0.42 | < 0.43 | < 0.41 | < 2.98 | < 0.41 | < 0.41 | < 0.41 | < 2.96 | < 3.98 | < 0.44 | < 0.76 | < 3.21 | < 0.41 |
| NMeFOSA | 31506-32-8 | < 1.05 | < 0.19 | < 2.98 | < 1.06 | < 1.09 | < 0.19 | < 2.98 | < 1.03 | < 1.04 | < 0.19 | < 2.96 | < 10.1 | < 1.12 | < 0.35 | < 3.21 | < 1.05 |
| NEtFOSA | 4151-50-2 | < 1.01 | < 0.54 | < 8.33 | < 1.02 | < 1.04 | < 0.54 | < 8.35 | < 0.99 | < 1.00 | < 0.55 | < 8.29 | < 9.72 | < 1.08 | < 1.00 | < 8.99 | < 1.01 |
| PFBSA | 30334-69-1 | | < 0.09 | | | | < 0.09 | | | | < 0.30 | | | | < 0.10 | | |
| PFHxSA | 41997-13-1 | | < 0.09 | | | | < 0.09 | | | | < 0.30 | | | | < 0.10 | | |
| PFHpSA | 82765-77-3 | | < 0.09 | | | | < 0.09 | | | | < 0.30 | | | | < 0.10 | | |
| PFDSA | 4262-70-8 | | < 0.09 | | | | < 0.09 | | | | < 0.30 | | | | < 0.10 | | |
| NMeFBSA | 68298-12-4 | | < 0.19 | | | | < 0.19 | | | | < 0.59 | | | | < 0.19 | | |
| NMeFOSAA | 2355-31-9 | < 0.72 | < 0.33 | < 2.98 | < 0.73 | < 0.74 | < 0.33 | < 2.98 | < 0.71 | < 0.710 | < 0.33 | < 2.96 | < 6.91 | < 0.77 | < 0.61 | < 3.21 | < 0.719 |
| NEtFOSAA | 2991-50-6 | < 0.72 | < 0.24 | < 2.98 | < 0.73 | < 0.74 | < 0.24 | < 2.98 | < 0.70 | < 0.708 | < 0.24 | < 2.96 | < 6.89 | < 0.77 | < 0.45 | < 3.21 | < 0.717 |
| NMeFBSAA | 159381-10-9 | | < 0.19 | | | | < 0.19 | | | | < 0.59 | | | | < 0.19 | | |
| NMeFHSAA | 715646-50-7 | | < 0.19 | | | | < 0.19 | | | | < 0.59 | | | | < 0.19 | | |
| PFOSAA | 2806-24-8 | | < 0.19 | | | | < 0.19 | | | | < 0.59 | | | | < 0.19 | | |
| HFPO-DA | 13252-13-6 | < 1.77 | < 0.38 | | < 1.79 | < 1.83 | < 0.38 | | < 1.73 | < 1.75 | < 0.39 | | < 17.0 | < 1.89 | < 0.71 | | < 1.77 |
| ADONA | 919005-14-4 | < 1.66 | < 1.22 | | < 1.67 | < 1.71 | < 1.23 | | < 1.62 | < 1.63 | < 1.23 | | < 15.9 | < 1.77 | < 2.27 | | < 1.65 |
| PFMBA | 863090-89-5 | < 0.47 | < 0.11 | | < 0.48 | < 0.49 | < 0.11 | | < 0.46 | < 0.46 | < 0.11 | | < 4.51 | < 0.50 | < 0.21 | | < 0.47 |
| NFDHA | 151772-58-6 | < 1.61 | < 6.38 | | < 1.63 | < 1.67 | < 6.45 | | < 1.58 | < 1.59 | < 6.47 | | < 15.5 | < 1.73 | < 11.90 | | < 1.61 |
| PFMPA | 377-73-1 | < 0.71 | < 0.17 | | < 0.72 | < 0.73 | < 0.17 | | < 0.70 | < 0.70 | < 0.17 | | < 6.83 | < 0.76 | < 0.31 | | < 0.71 |
| PFMOAA | 674-13-5 | | < 1.90 | | | | < 1.90 | | | | < 5.96 | | | | < 1.93 | | |
| PFO3OA | 39492-89-2 | | < 1.90 | | | | < 1.90 | | | | < 5.96 | | | | < 1.93 | | |
| PFO4DA | 39492-90-5 | | < 1.90 | | | | < 1.90 | | | | < 5.96 | | | | < 1.93 | | |
| PFO5DoDA | 39492-91-6 | | < 1.88 | | | | < 1.88 | | | | < 5.91 | | | | < 1.91 | | |
| PFO6TeA | N/A | | < 1.90 | | | | < 1.90 | | | | < 5.96 | | | | < 1.93 | | |
| 9Cl-PF3ONS | 756426-58-1 | < 2.00 | < 1.49 | | < 2.02 | < 2.06 | < 0.87 | | < 1.96 | < 1.97 | < 0.87 | | < 19.2 | < 2.14 | < 1.61 | | < 2.00 |
| 11Cl-PF3OUdS | 763051-92-9 | < 2.01 | < 1.49 | | < 2.03 | < 2.07 | < 0.97 | | < 1.97 | < 1.98 | < 0.97 | | < 19.3 | < 2.15 | < 1.78 | | < 2.01 |
| PFEESA | 113507-82-7 | < 0.39 | < 0.37 | | < 0.39 | < 0.40 | < 0.13 | | < 0.38 | < 0.38 | < 0.13 | | < 3.72 | < 0.41 | < 0.24 | | < 0.387 |

# Table S3-G: PFAS Data - Site D (cont'd)

| Abbreviation | CAS Number | Oct-24 | Nov-24 | | Dec-24 | Jan-25 | Feb-25 | | Mar-25 | Apr-25 | May-25 | | Jun-25 | Jul-25 | Aug-25 | | Sep-25 |
|---|---|---|---|---|---|---|---|---|---|---|---|---|---|---|---|---|---|
| | | Effluent | Effluent | Effluent (post-TOP) | Effluent | Effluent | Effluent | Effluent (post-TOP) | Effluent | Effluent | Effluent | Effluent (post-TOP) | Effluent | Effluent | Effluent | Effluent (post-TOP) | Effluent |
| Nafion BP 1 | 29311-67-9 | | < 0.19 | | | | < 0.19 | | | | < 0.59 | | | | < 0.19 | | |
| Nafion BP2 | 749836-20-2 | | < 0.19 | | | | < 0.19 | | | | < 0.59 | | | | < 0.19 | | |
| PFecHS | 133201-07-7 | | < 0.25 | | | | < 0.25 | | | | < 0.77 | | | | < 0.25 | | |
| PFHxPA | 40143-76-8 | | < 2.35 | | | | < 2.35 | | | | < 7.38 | | | | < 2.39 | | |
| PFOPA | 40143-78-0 | | < 2.35 | | | | < 2.35 | | | | < 7.38 | | | | 2.74 | | |
| PFDPA | 52299-26-0 | | < 2.35 | | | | < 2.35 | | | | < 7.38 | | | | 3.63 | | |
| 6:2 PAP | 57678-01-0 | | < 2.65 | | | | < 15.20 | | | | < 7.38 | | | | 9.79 | | |
| 8:2 PAP | 57678-03-2 | | < 2.39 | | | | < 2.39 | | | | < 7.50 | | | | 12.9 | | |
| 6:6 PFPi | 40143-77-9 | | < 0.09 | | | | < 0.09 | | | | < 0.30 | | | | < 0.10 | | |
| 6:8 PFPi | 610800-34-5 | | < 0.09 | | | | < 0.09 | | | | < 0.30 | | | | < 0.10 | | |
| 8:8 PFPi | 40143-79-1 | | < 0.09 | | | | < 0.09 | | | | < 0.30 | | | | < 0.10 | | |
| 6:2 diPAP | 57677-95-9 | | 12.2 | | | | 7.08 | | | | 6.36 | | | | 19 | | |
| 8:2 diPAP | 678-41-1 | | < 0.98 | | | | < 0.67 | | | | < 1.48 | | | | < 0.48 | | |
| 4:2 FTOH | 2043-47-2 | | < 132 | | | | < 78.50 | | | | < 181 | | | | < 81.40 | | |
| 6:2 FTOH | 647-42-7 | | < 12.60 | | | | 12.1 | | | | 19.4 | | | | 22.4 | | |
| 7:2 sFTOH | 24015-83-6 | | < 21.20 | | | | < 5.88 | | | | < 3.66 | | | | < 6.00 | | |
| 8:2 FTOH | 678-39-7 | | < 2.53 | | | | < 7.54 | | | | < 4.95 | | | | < 4.09 | | |
| 10:2 FTOH | 865-86-1 | | < 0.89 | | | | < 1.02 | | | | < 1.01 | | | | 1.56 | | |
| 12:2 FTOH | 39239-77-5 | | < 0.89 | | | | < 1.02 | | | | < 1.01 | | | | < 1.01 | | |
| AOF (µg/L) | N/A | | 4.3 | | | | 2.78 | | | | 5.85 | | | | 4.2 A | | |

All values are in units of ng/L. CAS = Chemical Abstracts Service registry number. J = The amount detected is below the Reporting Limit/Limit of Quantitation. K = Peak detected but did not meet quantification criteria. Result reported represents the estimated maximum possible concentration. I = Ion transition ratio is outside of the acceptance criteria. A = Sample analyzed out of hold time. H = Concentration is estimated.

# Table S3-H: PFAS Data - Septic Tank Sampling

| Abbreviation | CAS Number | Site A | | | | | | Site B | | | | | |
|---|---|---|---|---|---|---|---|---|---|---|---|---|---|
| | | Supernatant (ng/L) | | | Septage (ng/g) | | | Supernatant (ng/L) | | | Septage (ng/g) | | |
| | | Tank 1 | Tank 2 | Tank 3 | Tank 1 | Tank 2 | Tank 3 | Tank 1 | Tank 2 | Tank 3 | Tank 1 | Tank 2 | Tank 3 |
| PFPrA | 422-64-0 | | | | | | | | | | | | |
| PFBA | 375-22-4 | 30.1 | 2.7 J | 3.37 J | 3.15 J | < 2.89 | < 2.88 | < 1.68 | < 16.0 | < 1.68 | < 7.27 | < 7.24 | < 16.0 |
| PFPeA | 2706-90-3 | 143 | 3.13 J | 3.75 | 3.02 J | < 1.52 | < 1.52 | 0.674 I J | < 4.19 | 2.3 I J | < 3.83 | < 3.82 | < 4.19 |
| PFHxA | 307-24-4 | 158 | 2.07 | 3.56 | 14.3 | < 1.50 | < 1.50 | < 0.29 | < 2.73 | 2.64 | < 3.78 | < 3.76 | < 2.73 |
| PFHpA | 375-85-9 | 16.3 | 0.524 J F | < 0.336 | < 1.38 | < 1.38 | < 1.37 | 0.436 J | < 2.71 | 0.464 J | < 3.47 | < 3.45 | < 2.71 |
| PFOA | 335-67-1 | 39.8 | 1.22 J | < 0.730 | 9.6 | < 2.87 | < 2.86 | < 1.86 | < 17.8 | 1.91 J | < 7.22 | < 7.19 | < 17.8 |
| PFNA | 375-95-1 | 1.47 J | < 0.508 | < 0.528 | < 1.14 | < 1.14 | < 1.14 | < 0.25 | < 2.42 | 0.878 J | < 2.87 | < 2.85 | < 2.42 |
| PFDA | 335-76-2 | 7.77 | < 0.653 | < 0.680 | 5.1 | < 0.957 | < 0.955 | < 0.45 | < 4.25 | 0.823 J | < 2.41 | < 2.40 | < 4.25 |
| PFUnA | 2058-94-8 | < 0.679 | < 0.701 | < 0.730 | < 1.80 | < 1.80 | < 1.79 | < 0.43 | < 4.13 | < 0.43 | < 4.53 | < 4.51 | < 4.13 |
| PFDoDA | 307-55-1 | < 0.718 | < 0.742 | < 0.772 | 4.8 | 1.32 J | < 1.15 | < 0.23 | < 2.23 | < 0.23 | < 2.89 | < 2.88 | < 2.23 |
| PFTrDA | 72629-94-8 | < 0.585 | < 0.605 | < 0.629 | 1.81 J | < 0.858 | < 0.856 | < 0.27 | < 2.56 | < 0.27 | < 2.16 | < 2.15 | < 2.56 |
| PFTeDA | 376-06-7 | < 0.414 | < 0.427 | < 0.445 | 1.58 J | < 1.08 | < 1.08 | < 0.25 | < 2.39 | < 0.25 | < 2.72 | < 2.71 | < 2.39 |
| PFHxDA | 67905-19-5 | | | | | | | | | | | | |
| PFODA | 16517-11-6 | | | | | | | | | | | | |
| PFMeS | 37181-39-8 | | | | | | | | | | | | |
| PFEtS | 354-88-1 | | | | | | | | | | | | |
| PFPrS | 423-41-6 | | | | | | | | | | | | |
| PFBS | 375-73-5 | 10.4 F | 4.36 | 4.71 | < 1.66 | < 1.66 | < 1.66 | 1.36 J | 11.7 I J | 1.53 | < 4.19 | < 4.17 | < 6.75 |
| PFPeS | 2706-91-4 | < 0.273 | < 0.282 | < 0.294 | < 1.08 | < 1.08 | < 1.08 | < 0.51 | < 4.84 | < 0.51 | < 2.72 | < 2.71 | < 4.84 |
| PFHxS | 355-46-4 | < 0.375 | 0.613 J F | < 0.403 | < 1.75 | < 1.75 | < 1.75 | < 0.53 | < 5.04 | < 0.53 | < 4.41 | < 4.39 | < 5.04 |
| PFHpS | 375-92-8 | < 0.422 | < 0.435 | < 0.453 | < 1.47 | < 1.47 | < 1.46 | < 0.40 | < 3.77 | < 0.40 | < 3.69 | < 3.68 | < 3.77 |
| PFOS | 1763-23-1 | 0.984 J | 4.59 F | < 0.763 | < 2.05 | 2.92 J | < 2.04 | 9.98 I | < 11.7 | 5.16 I | < 5.16 | < 5.14 | < 11.7 |
| PFNS | 68259-12-1 | < 0.484 | < 0.500 | < 0.520 | < 1.56 | < 1.56 | < 1.55 | < 0.63 | < 6.04 | < 0.63 | < 3.92 | < 3.91 | < 6.04 |
| PFDS | 335-77-3 | < 0.359 | < 0.371 | < 0.386 | < 2.17 | < 2.17 | < 2.16 | < 0.59 | < 5.64 | < 0.59 | < 5.46 | < 5.44 | < 5.64 |
| PFDoS | 79780-39-5 | < 0.593 | < 0.613 | < 0.638 | < 1.60 | < 1.60 | < 1.60 | < 0.52 | < 4.97 | < 0.52 | < 4.03 | < 4.02 | < 4.97 |
| 3:3 FTCA | 356-02-5 | 7.14 J | < 2.66 | < 2.77 | < 4.58 | < 4.58 | < 4.57 | < 1.66 | < 15.8 | < 1.65 | < 11.5 | < 11.5 | < 15.8 |
| 5:3 FTCA | 914637-49-3 | 396 | < 9.43 | 17.2 J | 114 | < 28.7 | < 28.6 | < 7.09 | < 67.7 | < 7.09 | < 72.2 | < 71.9 | < 67.7 |
| 7:3 FTCA | 812-70-4 | 117 | < 6.36 | < 6.62 | 86.8 J | < 27.9 | < 27.8 | < 3.76 | < 35.9 | < 3.76 | < 70.2 | < 69.9 | < 35.9 |
| 6:2 FTCA | 53826-12-3 | | | | | | | | | | | | |
| 8:2 FTCA | 27854-31-5 | | | | | | | | | | | | |
| 10:2 FTCA | 53826-13-4 | | | | | | | | | | | | |
| 6:2 FTUCA | 70887-88-6 | | | | | | | | | | | | |
| 8:2 FTUCA | 70887-84-2 | | | | | | | | | | | | |
| 4:2 FTS | 757124-72-4 | < 1.63 | < 1.68 | < 1.75 | < 6.13 | < 6.13 | < 66.11 | < 1.33 | < 12.7 | < 1.33 | < 15.4 | < 15.4 | < 12.7 |
| 6:2 FTS | 27619-97-2 | < 2.11 | < 2.18 | < 2.26 | < 3.17 | < 3.16 | < 3.16 | < 1.28 | < 12.2 | < 1.28 | < 7.97 | < 7.94 | < 12.2 |
| 8:2 FTS | 39108-34-4 | < 2.43 | < 2.51 | < 2.61 | < 7.19 | <7.18 | < 7.17 | < 1.96 | < 18.7 | < 1.96 | < 18.1 | < 18.0 | < 18.7 |
| 10:2 FTS | 120226-60-0 | | | | | | | | | | | | |
| NMeFOSE | 24448-09-7 | < 3.67 | < 3.79 | < 3.94 | < 11.7 | < 11.7 | < 11.7 | 8.34 J | < 26.0 | < 2.72 | < 29.6 | < 29.4 | < 26.0 |
| NEtFOSE | 1691-99-2 | < 1.91 | < 1.98 | < 2.06 | < 10.5 | < 10.5 | < 10.5 | 3.11 J | < 25.5 | < 2.67 | < 26.5 | < 26.4 | < 25.5 |
| PFOSA | 754-91-6 | 0.749 J | < 0.435 | < 0.453 | < 1.51 | < 1.50 | < 1.50 | 0.635 I J | < 3.98 | < 0.42 | < 3.79 | < 3.77 | < 3.98 |
| NMeFOSA | 31506-32-8 | < 0.679 | < 0.701 | < 0.730 | < 1.80 | < 1.80 | < 1.80 | < 1.06 | < 10.1 | < 1.06 | < 4.54 | < 4.52 | < 10.1 |
| NEtFOSA | 4151-50-2 | < 0.718 | < 0.742 | < 0.772 | < 2.85 | < 2.85 | < 2.84 | < 1.02 | < 9.72 | < 1.02 | < 7.17 | < 7.14 | < 9.72 |
| PFBSA | 30334-69-1 | | | | | | | | | | | | |
| PFHxSA | 41997-13-1 | | | | | | | | | | | | |
| PFHpSA | 82765-77-3 | | | | | | | | | | | | |
| PFDSA | 4262-70-8 | | | | | | | | | | | | |
| NMeFBSA | 68298-12-4 | | | | | | | | | | | | |
| NMeFOSAA | 2355-31-9 | < 0.851 | < 0.879 | < 0.914 | < 1.59 | < 1.59 | < 15.8 | 2.37 | < 6.91 | 0.883 J | < 3.99 | < 3.98 | < 6.91 |
| NEtFOSAA | 2991-50-6 | 1.55 J | < 0.871 | < 0.906 | 1.38 J | < 1.22 | < 1.22 | < 0.72 | < 6.89 | < 0.72 | < 3.07 | < 3.06 | < 6.89 |
| NMeFBSAA | 159381-10-9 | | | | | | | | | | | | |
| NMeFHSAA | 715646-50-7 | | | | | | | | | | | | |

# Table S3-H: PFAS Data - Septic Tank Sampling (cont'd)

| Abbreviation | CAS Number | Site A | | | | | | Site B | | | | | |
|---|---|---|---|---|---|---|---|---|---|---|---|---|---|
| | | Supernatant (ng/L) | | | Septage (ng/g) | | | Supernatant (ng/L) | | | Septage (ng/g) | | |
| | | Tank 1 | Tank 2 | Tank 3 | Tank 1 | Tank 2 | Tank 3 | Tank 1 | Tank 2 | Tank 3 | Tank 1 | Tank 2 | Tank 3 |
| PFOSAA | 2806-24-8 | | | | | | | | | | | | |
| HFPO-DA | 13252-13-6 | < 0.874 | < 0.903 | < 0.940 | < 6.71 | < 6.71 | < 6.69 | < 1.78 | < 17.00 | < 1.78 | < 16.9 | < 16.8 | < 17.0 |
| ADONA | 919005-14-4 | < 0.984 | < 1.02 | < 1.06 | < 4.56 | < 4.56 | < 4.55 | < 1.67 | < 15.90 | < 1.67 | < 11.5 | < 11.4 | < 15.9 |
| PFMBA | 863090-89-5 | < 0.414 | < 0.427 | < 0.445 | < 2.03 | < 2.03 | < 2.02 | < 0.47 | < 4.51 | < 0.47 | < 5.11 | < 5.09 | < 4.51 |
| NFDHA | 151772-58-6 | < 1.84 | < 1.90 | < 1.98 | < 4.12 | < 4.12 | < 4.11 | < 1.62 | < 15.50 | < 1.62 | < 10.4 | < 10.3 | < 15.5 |
| PFMPA | 377-73-1 | < 0.445 | < 0.460 | < 0.478 | < 2.15 | < 2.15 | < 2.14 | < 0.72 | < 6.83 | < 0.72 | < 5.41 | < 5.39 | < 6.83 |
| PFMOAA | 674-13-5 | | | | | | | | | | | | |
| PFO3OA | 39492-89-2 | | | | | | | | | | | | |
| PFO4DA | 39492-90-5 | | | | | | | | | | | | |
| PFO5DoDA | 39492-91-6 | | | | | | | | | | | | |
| PFO6TeA | N/A | | | | | | | | | | | | |
| 9Cl-PF3ONS | 756426-58-1 | < 1.29 | < 1.33 | < 1.38 | < 4.92 | < 4.91 | < 4.90 | < 2.01 | < 19.20 | < 2.01 | < 12.4 | < 12.3 | < 19.2 |
| 11Cl-PF3OUdS | 763051-92-9 | < 1.29 | < 1.33 | < 1.38 | < 3.60 | < 3.60 | < 3.59 | < 2.02 | < 19.30 | < 2.02 | < 9.07 | < 9.03 | < 19.30 |
| PFEESA | 113507-82-7 | < 0.343 | < 0.355 | < 0.369 | < 2.27 | < 2.27 | < 2.26 | < 0.39 | < 3.72 | < 0.39 | < 5.71 | < 5.69 | < 3.72 |
| Nafion BP 1 | 29311-67-9 | | | | | | | | | | | | |
| Nafion BP2 | 749836-20-2 | | | | | | | | | | | | |
| PFecHS | 133201-07-7 | | | | | | | | | | | | |
| PFHxPA | 40143-76-8 | | | | | | | | | | | | |
| PFOPA | 40143-78-0 | | | | | | | | | | | | |
| PFDPA | 52299-26-0 | | | | | | | | | | | | |
| 6:2 PAP | 57678-01-0 | | | | | | | | | | | | |
| 8:2 PAP | 57678-03-2 | | | | | | | | | | | | |
| 6:6 PFPi | 40143-77-9 | | | | | | | | | | | | |
| 6:8 PFPi | 610800-34-5 | | | | | | | | | | | | |
| 8:8 PFPi | 40143-79-1 | | | | | | | | | | | | |
| 6:2 diPAP | 57677-95-9 | | | | | | | | | | | | |
| 8:2 diPAP | 678-41-1 | | | | | | | | | | | | |
| 4:2 FTOH | 2043-47-2 | | | | | | | | | | | | |
| 6:2 FTOH | 647-42-7 | | | | | | | | | | | | |
| 7:2 sFTOH | 24015-83-6 | | | | | | | | | | | | |
| 8:2 FTOH | 678-39-7 | | | | | | | | | | | | |
| 10:2 FTOH | 865-86-1 | | | | | | | | | | | | |
| 12:2 FTOH | 39239-77-5 | | | | | | | | | | | | |

CAS = Chemical Abstracts Service registry number. J = The amount detected is below the Reporting Limit/Limit of Quantitation. I = Ion transition ratio is outside of the acceptance criteria. F = The ratio of quantifier ion response to qualifier ion response falls outside of the laboratory criteria. Results are considered to be an estimated maximum concentration.

## Section S4: Site Diagrams

Section S4 consists of aerial diagrams and process flow diagrams for Sites A, B, C, and D.

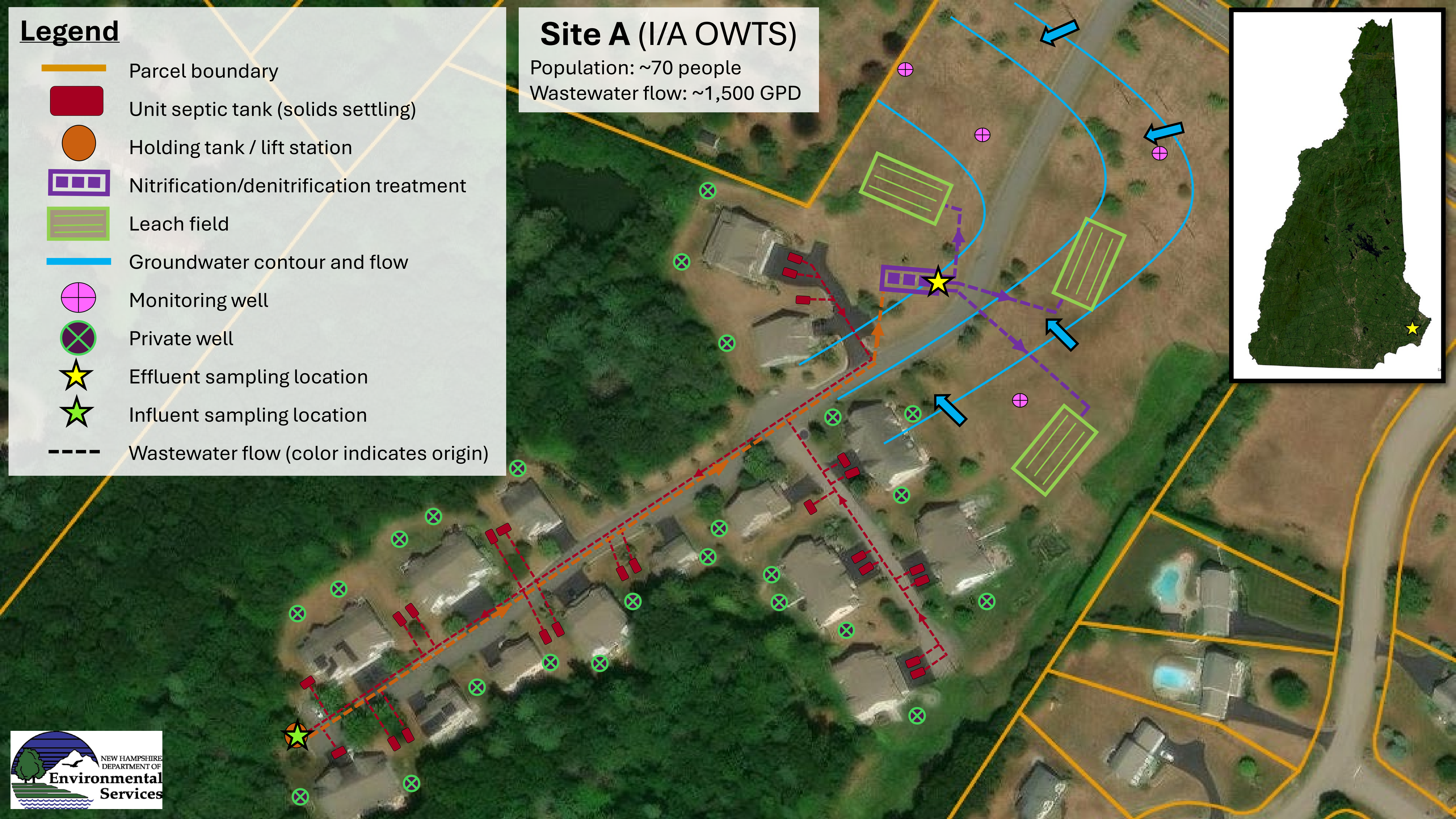

Site A (I/A OWTS)
Population: ~70 people
Wastewater flow: ~1,500 GPD
Legend
Parcel boundary
Unit septic tank (solids settling)
Holding tank / lift station
Nitrification/denitrification treatment
Leach field
Groundwater contour and flow
Monitoring well
Private well
Effluent sampling location
Influent sampling location
Wastewater flow (color indicates origin)
NEW HAMPSHIRE DEPARTMENT OF
Environmental Services

## **Site A** (I/A OWTS) Process Flow Diagram

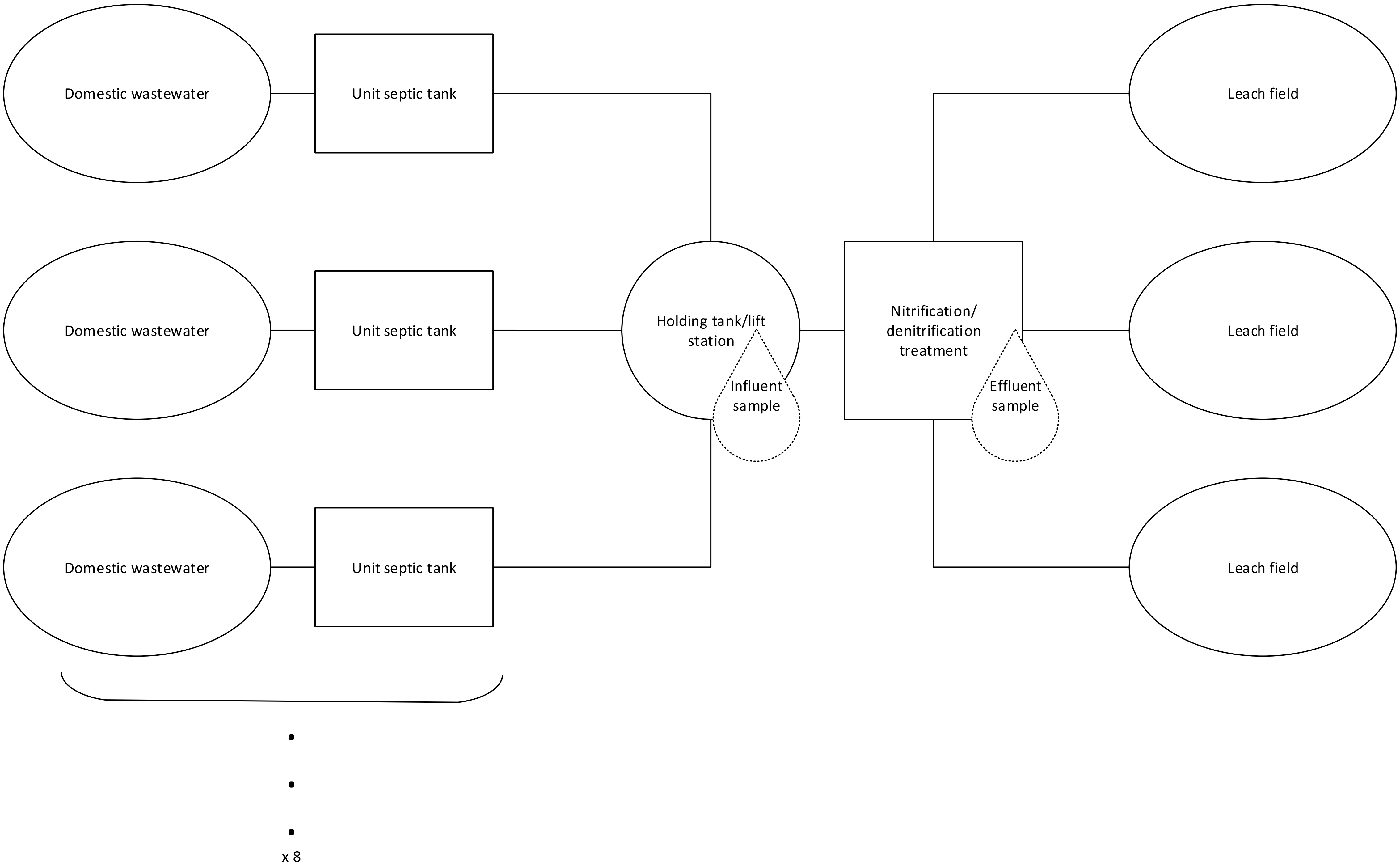

# **Site B** (Conventional)

Population: 60-100 people
Wastewater flow: ~700 GPD

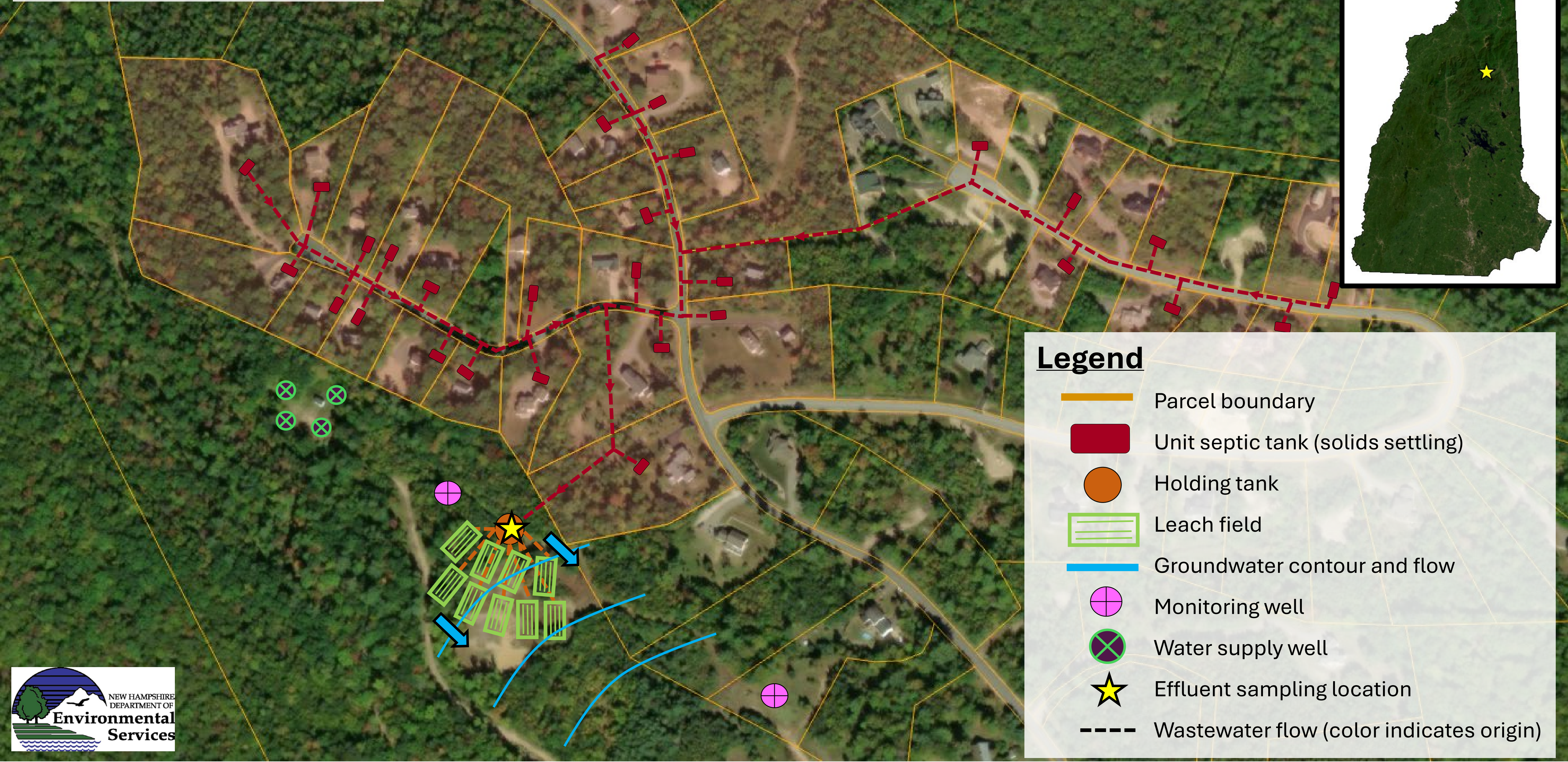

**Site B** (Conventional) Process Flow Diagram

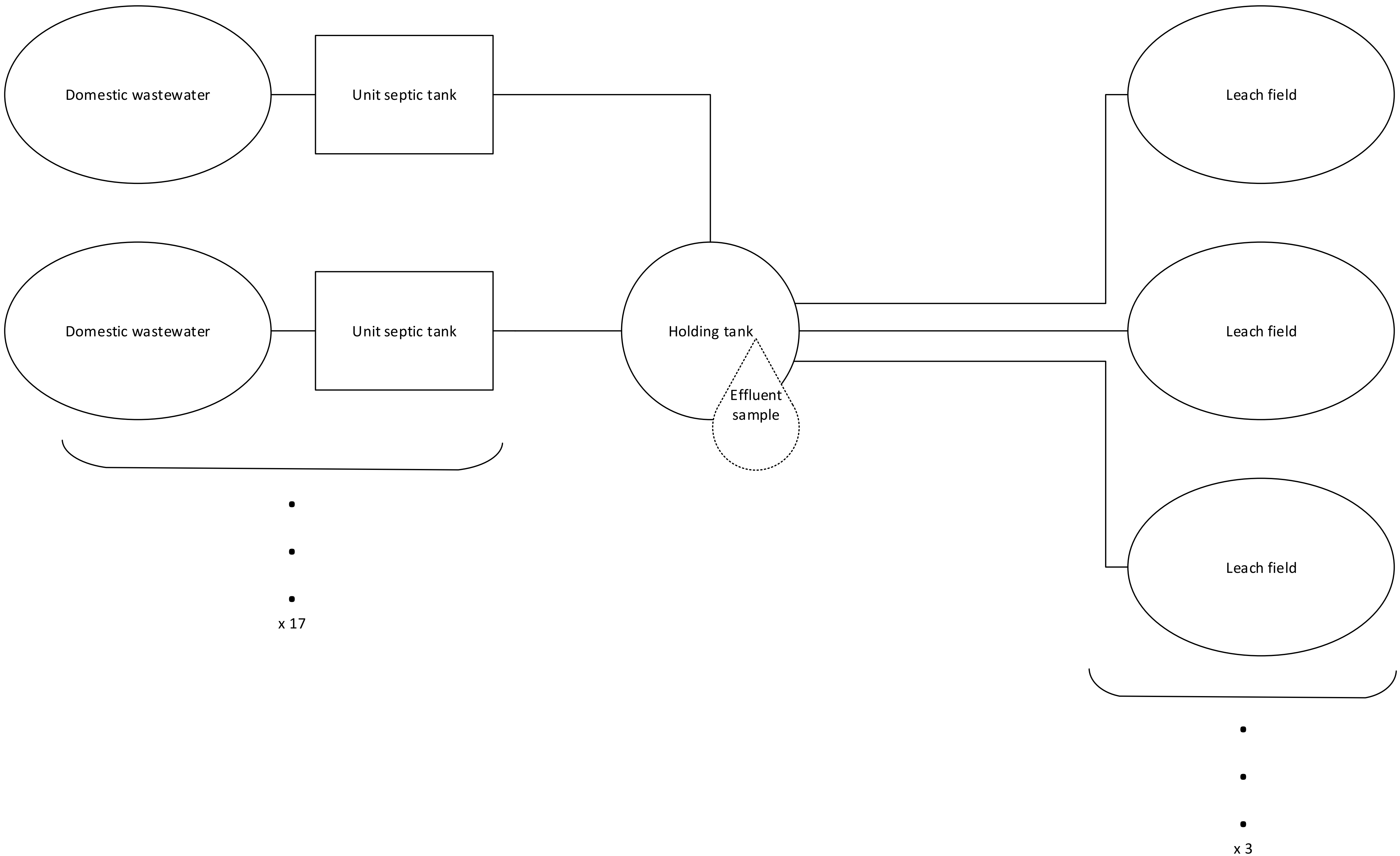

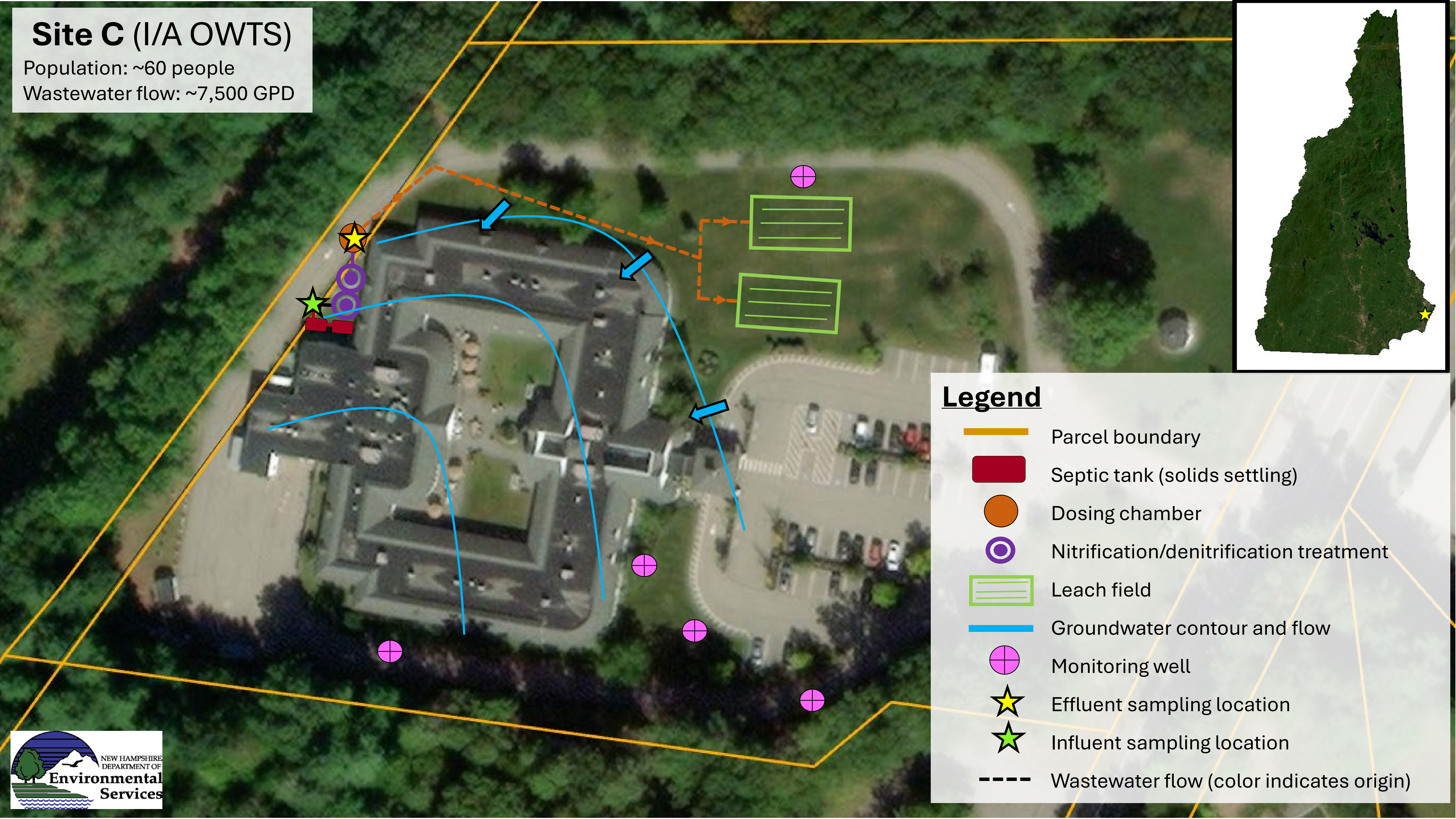
Site C (I/A OWTS)
Population: ~60 people
Wastewater flow: ~7,500 GPD
Legend
Parcel boundary
Septic tank (solids settling)
Dosing chamber
Nitrification/denitrification treatment
Leach field
Groundwater contour and flow
Monitoring well
Effluent sampling location
Influent sampling location
Wastewater flow (color indicates origin)
NEW HAMPSHIRE DEPARTMENT OF Environmental Services

**Site C** (I/A OWTS) Process Flow Diagram

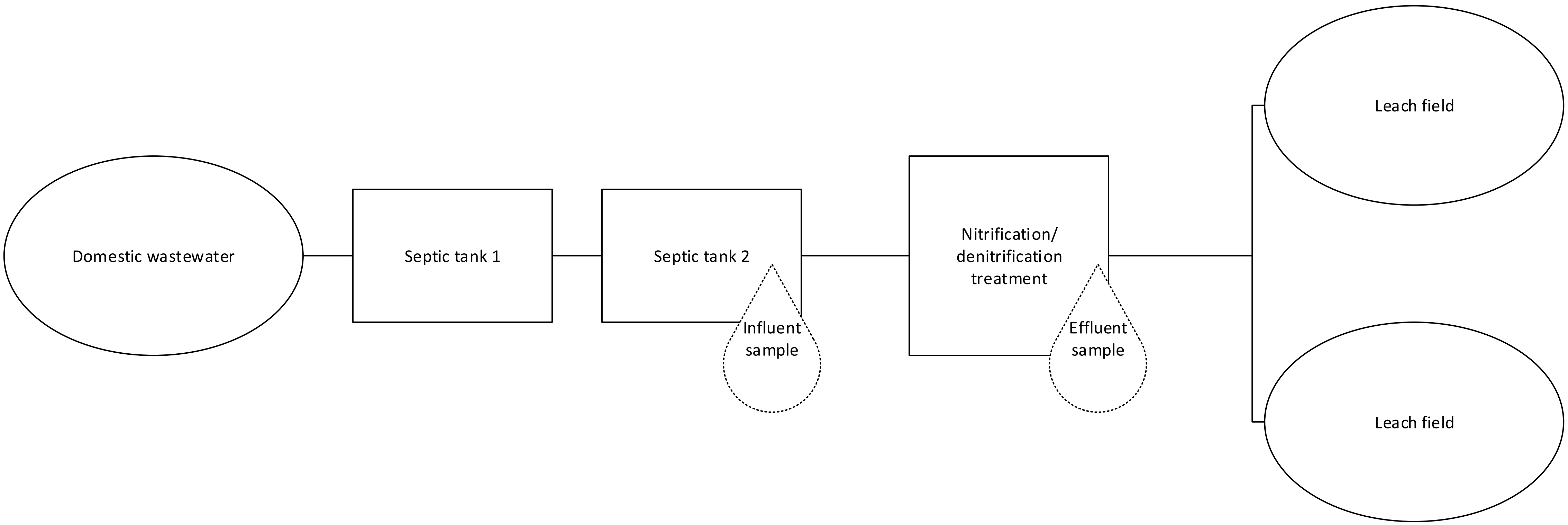

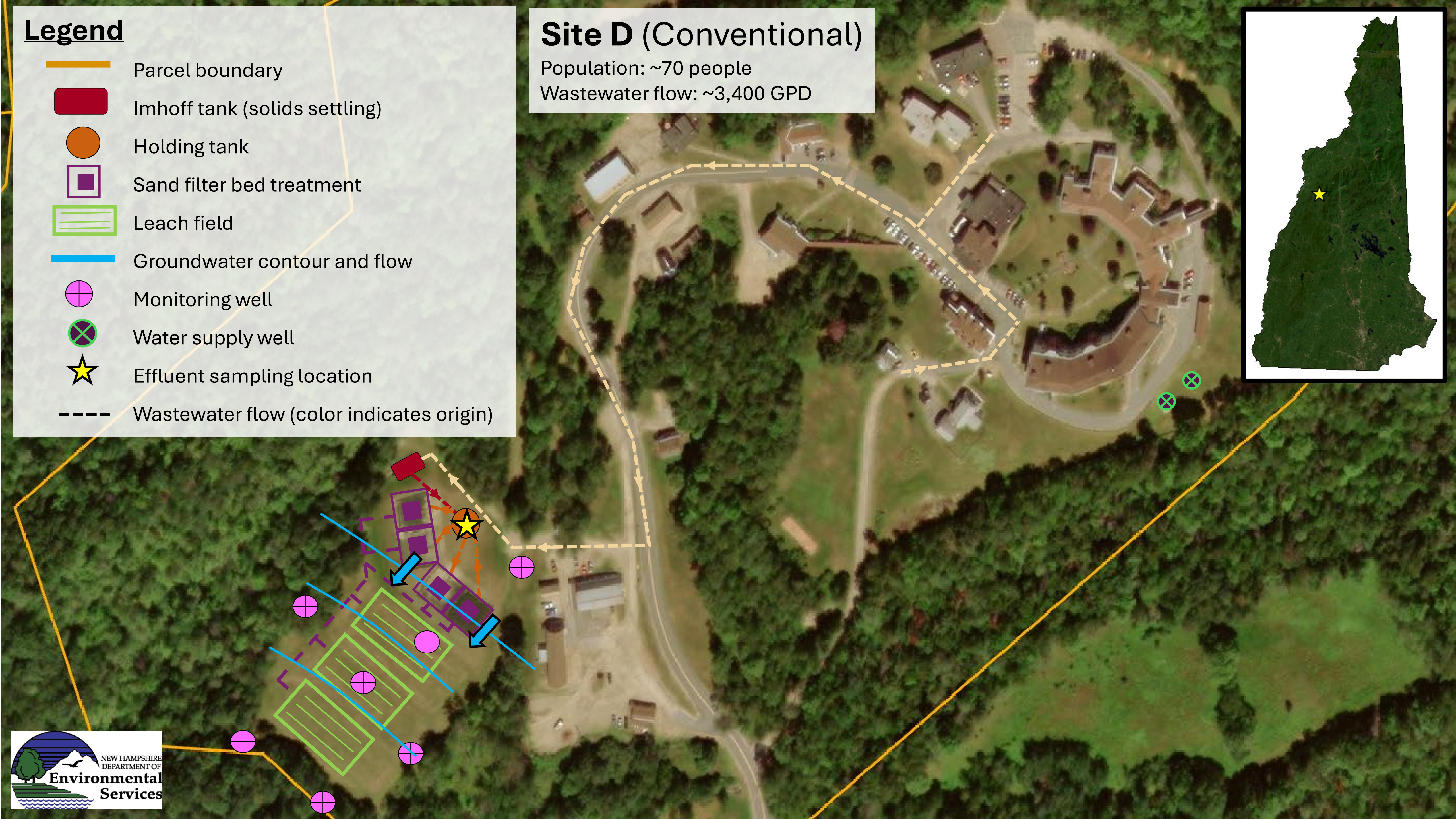
Site D (Conventional)
Population: ~70 people
Wastewater flow: ~3,400 GPD
Legend
Parcel boundary
Imhoff tank (solids settling)
Holding tank
Sand filter bed treatment
Leach field
Groundwater contour and flow
Monitoring well
Water supply well
Effluent sampling location
Wastewater flow (color indicates origin)
NEW HAMPSHIRE DEPARTMENT OF Environmental Services

**Site D** (Conventional) Process Flow Diagram

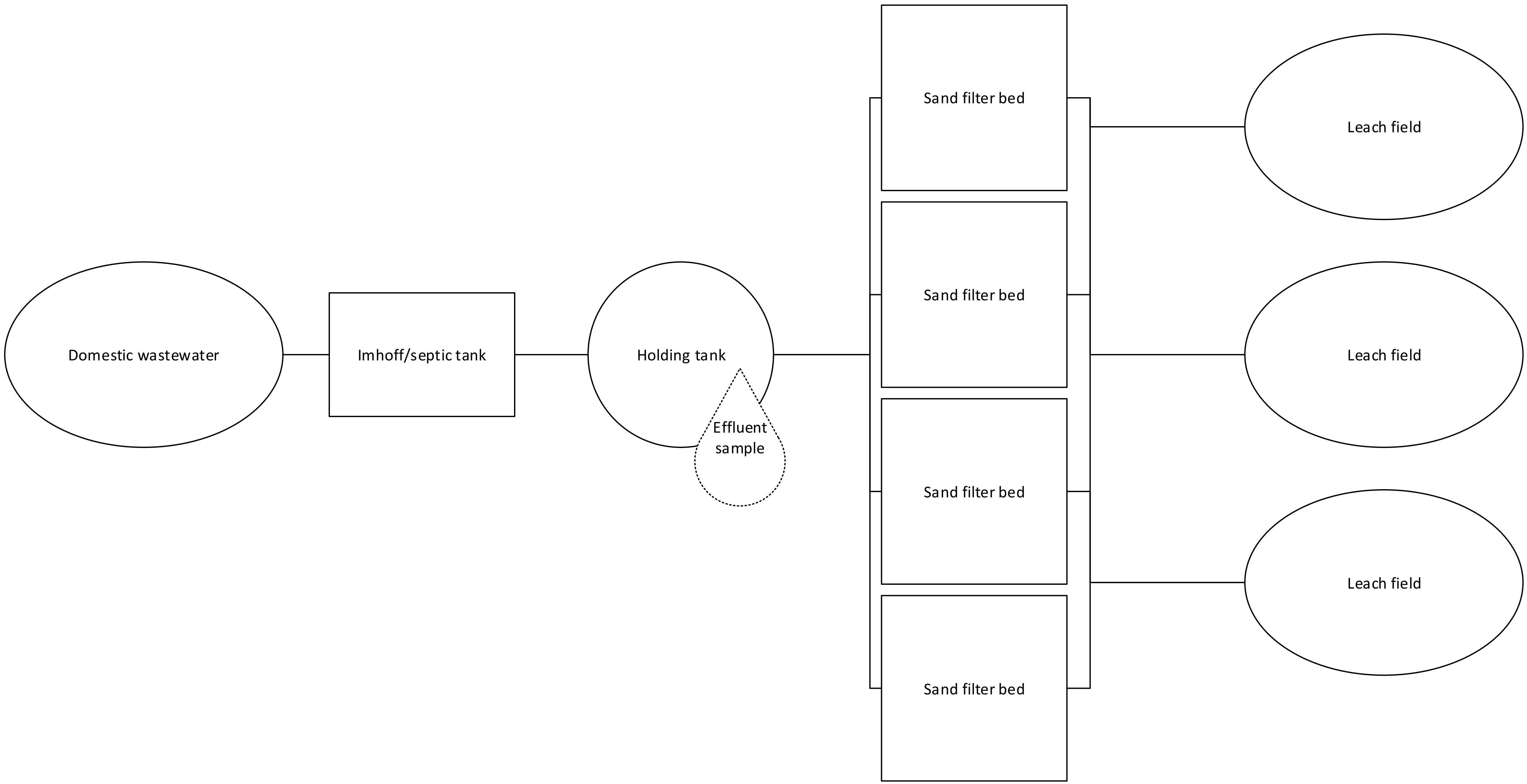

Supplement: Supplementary file 1 [file ew6c00198_si_001.pdf]
